# Noninvasive and Sensitive Biosensor for the Detection of Oral Cancer Prognostic Biomarkers

**DOI:** 10.1002/smll.202504278

**Published:** 2025-07-29

**Authors:** Luciana D. Trino Albano, Daniela C. Granato, Luiz G. S. Albano, Fábio M. S. Patroni, Aline G. Santana, Guilherme A. Câmara, Davi H. S. de Camargo, Ana L. Mores, Thaís B. Brandão, Ana C. Prado‐Ribeiro, Carlos C. B. Bufon, Adriana F. Paes Leme

**Affiliations:** ^1^ Center for Information Technology Renato Archer (CTI Renato Archer) Campinas São Paulo 13069‐901 Brazil; ^2^ Brazilian Biosciences National Laboratory (LNBio) Brazilian Center for Research in Energy and Materials (CNPEM) Campinas São Paulo 13083‐970 Brazil; ^3^ Brazilian Nanotechnology National Laboratory (LNNano) Brazilian Center for Research in Energy and Materials (CNPEM) Campinas São Paulo 13083‐970 Brazil; ^4^ Dental Oncology Service Instituto do Câncer do Estado de São Paulo Faculdade de Medicina da Universidade de São Paulo (ICESP‐FMUSP) São Paulo 01246‐000 Brazil; ^5^ Oral Diagnosis Department Semiology and Oral Pathology Areas Piracicaba Dental School State University of Campinas (UNICAMP) Piracicaba São Paulo 13414‐903 Brazil; ^6^ Physics Department Institute of Geosciences and Exact Sciences São Paulo State University (UNESP) Rio Claro São Paulo 13506‐900 Brazil

**Keywords:** biosensor, machine learning, metal‐organic frameworks, noninvasive analysis, oral cancer

## Abstract

Early detection of oral squamous cell carcinoma (OSCC) significantly enhances treatment outcomes and survival rates, with lymph node metastasis serving as a main prognostic factor. However, current clinical practices rely on TNM classification, including histological confirmation of metastatic disease in lymph nodes, often involving elective neck dissection, a procedure that can cause post‐operative morbidity. Here it is shown that zinc imidazole framework‐8 (ZIF‐8) electrochemical biosensors can effectively distinguish non‐metastatic (N0) from lymph node metastatic (N+) OSCC saliva samples. By monitoring the OSCC biomarkers cystatin B (CSTB), leukotriene A 4 hydrolase (LTA4H), and collagen type VI alpha 1 chain (COL6A1) in human saliva through electrochemical impedance spectroscopy and antigen–antibody immunoreactions, elevated biomarker levels in N0 samples are observed. The biosensor displays high accuracy, specificity, and reproducibility, with limits of detection lower than 0.4 ng mL^−1^. Supervised bioinformatic analysis, using 34 machine learning classifiers, indicates LTA4H as the most accurate biomarker for distinguishing prognostic groups, confirming previous mass spectrometry findings. Notably, the AdaBoost model, integrating the combined detection of biomarkers, achieves a 76% accuracy rate in identifying metastatic saliva samples. This non‐invasive biosensor technology, combined with bioinformatics, presents a sensitive and reliable approach to improve clinical assessments and guiding therapeutic decisions for OSCC patients.

## Graphical abstract

Oral cancer is often diagnosed with the presence of metastasis, which implies a significantly poor prognosis. Saliva‐based liquid biopsy using electrochemical biosensors targeting CSTB, LTA4H, and COL6A1 biomarkers offers high sensitivity and selectivity. In a cohort of 60 patients, machine learning analysis achieves 76% accuracy in detecting lymph node metastasis, enhancing non‐invasive clinical assessments for oral cancer.

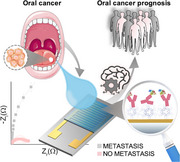

## 1 Introduction

Cancer is a significant cause of morbidity and the second leading cause of death worldwide. A 47% increase in cancer occurrence is estimated by 2040 from 19.3 million cases in 2020.^[^ ^1^ ^]^ Among the types of cancer, more than 389 000 new cases of oral cancer were estimated worldwide in 2022, exceeding 188 000 deaths.^[^ ^2^ ^]^ It is the eighth most common cancer site in men in the US and the third in South Central Asia and Melanesia.^[^ ^1^, ^3^ ^]^ The most prevalent type of oral cancer consists of squamous cell carcinomas, accounting for more than 90% of the cases.^[^ ^4^ ^]^ The occurrence of oral squamous cell carcinoma (OSCC) is attributed to multiple biological and environmental factors involved in the carcinogenic process. However, the majority of OSCC cases are related to exposure to carcinogenic promotors like tobacco and alcohol.^[^ ^5^, ^6^ ^]^ Moreover, these environmental risk factors have a synergetic effect. Alcohol increases the permeability of oral mucosa and might act dissolving tobacco products, consequently leading to an increased carcinogenic effect.^[^ ^7^ ^]^ Historically, oral cancer shows a particularly high death rate because it is usually discovered late in its development. It is often diagnosed with the presence of metastasis, most likely in the lymph nodes, which implies a significantly poor prognosis. Lymph node metastasis is confirmed by histological analysis of the tissue biopsy, often followed by radical neck dissection due to the high incidence (21%) of occult cervical metastases.^[^ ^8^, ^9^ ^]^ This traditional surgical procedure requires extensive resection in the neck area, which, although usually effective, frequently results in post‐operative morbidity, particularly manifesting as shoulder dysfunction.^[^ ^9^ ^]^ Unfortunately, in about 70% of cases, metastasis is not confirmed after the surgery.^[^ ^9^ ^]^ Additionally, the primary tumor in later stages can invade the local tissue, compromising the patient`s recovery. Due to the late diagnosis, the 5‐year survival rate for OSCC has remained almost unchanged over the past few decades, being around 50%.^[^ ^10^ ^]^ Therefore, there is an urgent demand to develop new methodologies to detect OSCC in the early stages for a successful treatment and increased overall survival rate.

A potential approach for early OSCC diagnosis and prognosis prediction is liquid biopsy testing from saliva, serum, and interstitial fluid.^[^ ^11^, ^12^, ^13^ ^]^ These biofluids are a rich source of biomarkers that can be used to verify disease development and therapeutic options. Due to the direct contact with the tumor site and tumoral microenvironment, saliva has emerged as a reliable source of OSCC biomarkers.^[^ ^13^, ^14^, ^15^, ^16^ ^]^ Additionally, it is easily obtainable and provides a non‐invasive monitoring of the disease. Establishing a correlation between biomarker levels in blood and saliva is complex due to physiological differences between these fluids, but some studies indicated that the use of saliva offers greater potential for the discovery of protein biomarkers for OSCC detection,^[^ ^17^ ^]^ particularly low molecular weight proteins.^[^ ^18^ ^]^ Recent progress in OSCC biomarker discovery from saliva samples has enabled the identification of peptides from cystatin B (CSTB), leukotriene A4 hydrolase (LTA4H), and collagen type VI alpha 1 chain (COL6A1) through mass spectrometry‐based proteomics as a robust prognostic signature, able to distinguish patients with and without lymph node metastasis.^[^ ^19^ ^]^ Consequently, monitoring the levels of these potential biomarkers may assist clinicians to better guide treatment strategies for OSCC patients.

Electrochemical biosensors offer substantial advantages compared to traditional biomarker detection methods, such as more accessible protocols and faster response.^[^ ^20^ ^]^ Different types of materials are available for the fabrication of biosensing platforms. Metal‐organic frameworks (MOFs) show a versatile range of combinations between metal clusters and organic ligands that confers their high porosity, large specific surface area, and a variety of surface functional groups for further functionalization. These unique advantages allowed MOFs to be increasingly applied in electronics,^[^ ^21^, ^22^, ^23^, ^24^ ^]^ electrocatalysis,^[^ ^25^ ^]^ energy storage devices,^[^ ^26^, ^27^ ^]^ and electrochemical biosensors.^[^ ^28^, ^29^, ^30^, ^31^ ^]^ Zinc imidazole framework‐8 (ZIF‐8), a MOF constituted by 2‐methylimidazole and zinc ions tetrahedrally arranged, possesses intrinsic specific capacitance that makes it an appropriate candidate for electrochemical sensor devices, particularly as a transducer.^[^ ^32^ ^]^ When ZIF‐8 interacts with the target analyte, a considerable shift in capacitance is observed, which can be described as a function of the impedance response.^[^ ^33^ ^]^

We developed ZIF‐8 electrochemical biosensors to monitor the OSCC signature proteins CSTB, LTA4H, and COL6A1 levels in saliva samples. The detection is based on the antigen‐antibody immunoreaction between each biomarker antibody and the specific analyte present in the biofluid. The biosensors exhibit high sensitivity and selectivity for the three biomarkers analyzed in a cohort of 60 OSCC saliva samples, 30 with (N+) and 30 without (N0) lymph node metastasis. Moreover, the difference in the CSTB, LTA4H, and COL6A1 levels indicates that higher concentrations are observed in N0 saliva samples compared to the N+. These findings agreed with our previous proteomics results,^[^ ^19^ ^]^ suggesting the effectiveness of the present method. Finally, a bioinformatics analysis using 17 distinct machine learning classifiers, both with and without hyperparameter optimization, was conducted to evaluate the independent and combined effects of the biomarkers. This analysis revealed that LTA4H proves to be the most accurate biomarker for differentiating the groups. These findings align with our previous mass spectrometry results, emerging LTA4H as the most reliable protein‐level signature for determining prognosis.^[^ ^19^ ^]^ Furthermore, considering the combined effect of the three biomarkers, the AdaBoost model accurately correlated the levels of potential OSCC biomarkers. This machine‐learning approach demonstrated that the combined effect of the three biomarkers could identify metastatic samples with 76% accuracy in the AUC‐ROC curve, highlighting its potential to improve the assessment of future OSCC cases. While a larger cohort is required for clinical validation, the innovation of this study lies in the application of ZIF‐8 in a previously unexplored clinical challenge, the noninvasive prognosis of oral cancer. By integrating ZIF‐8 with scalable microfabricated interdigitated electrodes (IDEs), demonstrating robust performance in real human saliva samples, and coupling biosensor outputs with machine learning algorithms, this platform presents a sensitive and reliable diagnostic tool. The combination of electrochemical detection with bioinformatics offers a sensitive and reliable tool that could assist clinicians in providing optimal treatment options for oral cancer patients.

## 2 Results and Discussion

### 2.1 Biosensor Complementary Characterization

**Figure 1:**
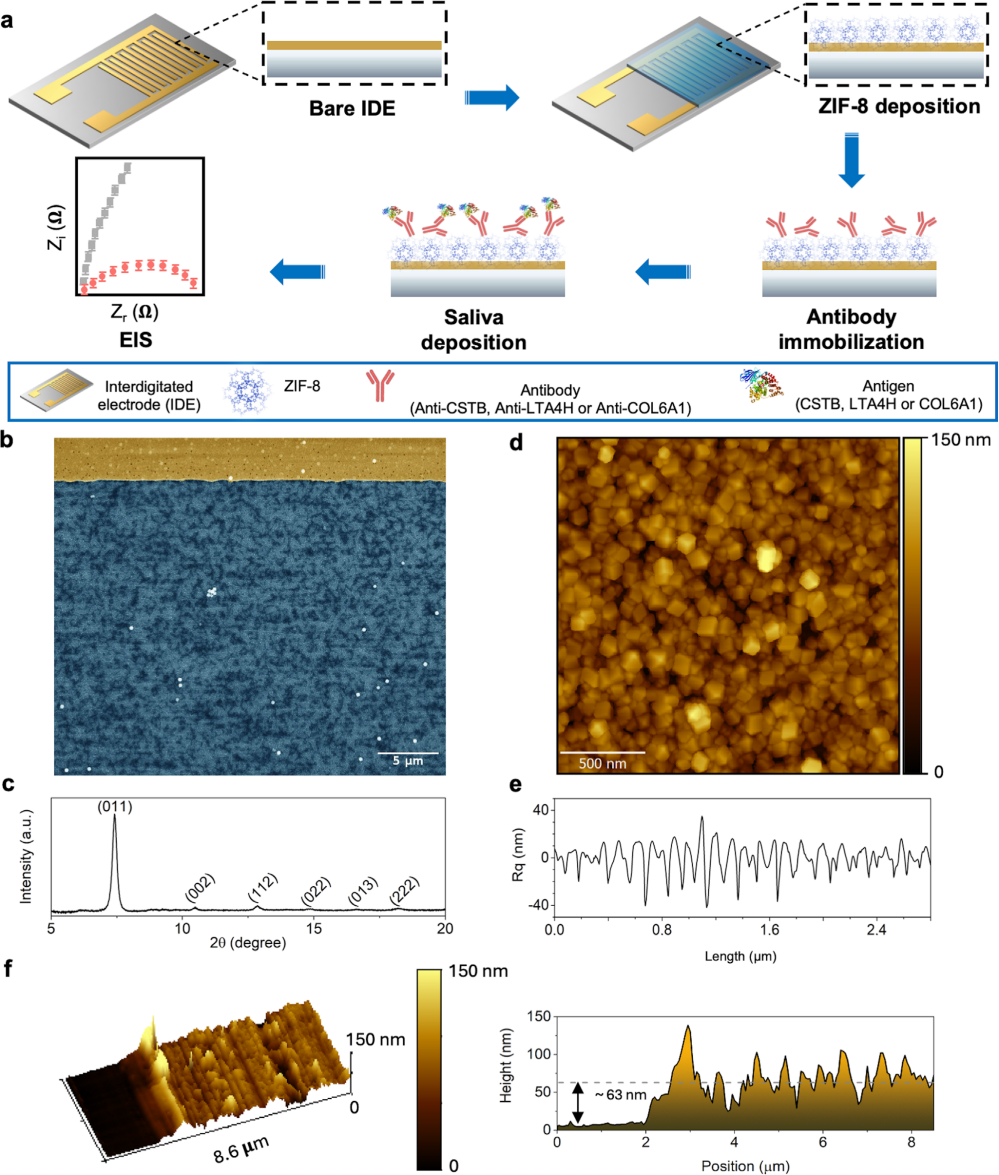
Biosensor development and complementary characterization. a) Schematic illustration of the biosensor fabrication for oral cancer prognostic biomarkers analysis. The IDE was fabricated by a photolithography process followed by ZIF‐8 growth. The respective antibody was immobilized on the IDE surface for specific identification of the biomarkers, followed by electrochemical impedance spectroscopy (EIS) analysis of saliva samples from N0 and N+ patients. b) SEM image showing the ZIF‐8 thin film formed between Au arrays. c) GIXRD pattern with the principal crystallographic planes of ZIF‐8 identified in parentheses. d) AFM image showing e) the homogeneous ZIF‐8 thin film with surface roughness around 11 (± 1) nm and f) thickness of 63 (± 14) nm.

The IDEs electrodes used for OSCC biomarker detection are modified following our previously reported device,^[^ ^28^ ^]^ combining ZIF‐8 as a transducer and specific antibodies to detect the proteins (**Figure** **1**a). The IDEs consist of 120 interlaced fingers, with a total electrode area (*A*) of 4.8 mm^2^, calculated using Equation (1):

(1) $$A=n_{d}L\left(W+2H\right)$$

where *n*_d_ is the number of digit electrode pairs, *L* is the length, *W* is the width, and *H* is the height.^[^ ^34^ ^]^ Additionally, the IDE photolithography fabrication process is illustrated in Figure S1 (Supporting Information). Figure 1b presents a scanning electron microscopy (SEM) image of the IDE after ZIF‐8 growth. The IDE before and after ZIF‐8 growth is represented in Figure S2 (Supporting Information). On the IDE active area, ZIF‐8 is grown in order to connect gold arrays and act as an anchoring site for antibody immobilization. After 6 h of growth, a uniform ZIF‐8 thin film is obtained with particles mostly measuring 159 (± 22) nm. However, a few larger particles of 252 (± 34) nm emerge on the top surface due to the extended exposure time (Figure S2b, Supporting Information), based on the well‐known Ostwald ripening effect.^[^ ^35^ ^]^ This phenomenon involves the dissolution of smaller particles and the regrowth of their constituents onto larger ones, driven by the greater instability of surface molecules. Therefore, larger particles are energetically favored. In the case of ZIF‐8, this process favors the (011) plane, leading to rhombic‐dodecahedral‐shaped particles.^[^ ^35^ ^]^

The crystallinity of ZIF‐8 thin film was analyzed by grazing incidence X‐ray diffraction (GIXRD), Figure 1c. As can be seen, characteristic ZIF‐8 peaks are observed, including the high‐intensity peak at 7.4°, which corresponds to the preferential growth orientation at (011) plane from the rhombic dodecahedral structure, the thermodynamically most stable ZIF‐8 crystalline form. Figure 1d shows the AFM topography image for ZIF‐8 thin film. As can be seen, for the 2 µm × 2 µm area, the pristine ZIF‐8 thin film has a homogeneous surface without significant pinholes, showing a root‐mean‐square (Rq) roughness around 11 (± 1) nm (Figure 1e). Moreover, the thin film thickness is 63 (± 14) nm, Figure 1f.

### 2.2 Antibody Immobilization

**Figure 2:**
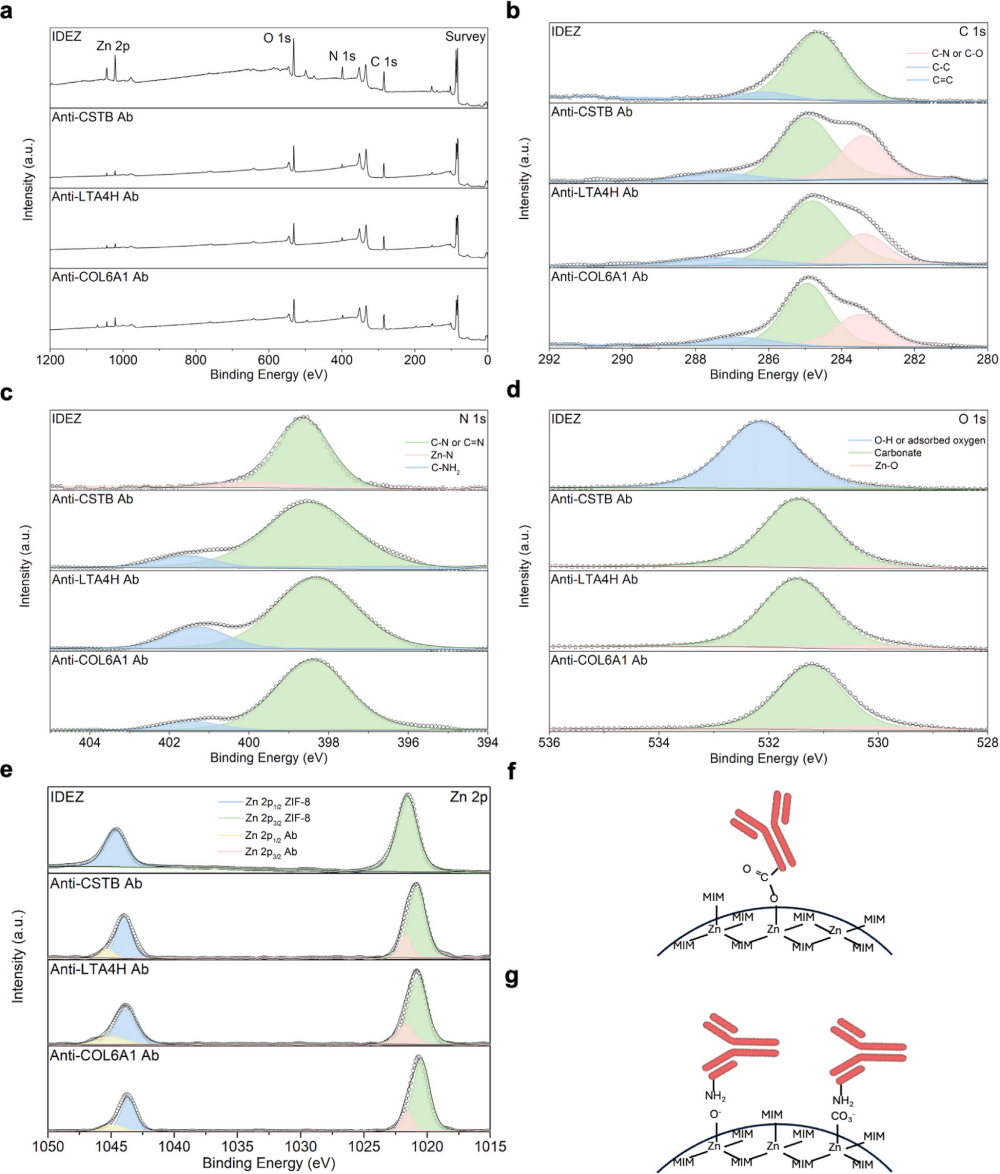
XPS analyses on the surface of the CSTB, LTA4H, and COL6A1 biosensors. To determine the chemical changes in the presence of antibodies, the biosensor modified with only ZIF‐8 was also analyzed.a) Survey spectrum for the analyzed samples, highlighting the main elements present. The high‐resolution spectra of b) C 1s, c) N 1s, d) O 1s, and e) Zn 2p show the type of chemical bonding of the elements. f) Based on XPS data, the antibodies may be immobilized directly through interaction with Zn metal ions, as there are defects on the surface where metal ions are not coordinated with the 2‐methylimidazole (MIM) ligand. g) Another possible mode of antibody adsorption is through hydroxyl and carbonate groups present on the ZIF‐8 surface.

The antibodies from CSTB, LTA4H, and COL6A1 proteins were immobilized upon IDE modified with ZIF‐8. The stability of the ZIF‐8 nanoparticles after incubation with the antibodies is shown in Figure S3 (Supporting Information). To determine the antibody immobilization mode on the biosensor surface, X‐ray photoelectron spectroscopy (XPS) analyses were performed (**Figure** **2**). XPS characterized the thin film composition, monitoring a broad binding energy region (0−1200 eV) and highlighting the presence of Zn 2p, O 1s, N 1s, and C 1s (Figure 2a). Several regions were scanned at higher resolution to obtain information about the chemical interactions in the ZIF‐8 thin films. The high‐resolution C 1s spectrum shows the presence of mainly C─C groups (284.8 eV) and C─N/C─O at 287 eV from carbonates or 2‐methylimidazole (MIM) ligands on the surface, respectively (Figure 2b).^[^ ^36^ ^]^ After antibody immobilization, an increase in the C─N/C─O contribution and an evident peak at 283 eV, relative to the C═C bond, are observed.^[^ ^37^ ^]^ This can be attributed to the polypeptide chains that constitute the antibodies: two identical light chains, each with approximately 220 amino acids (25 kDa), and two identical heavy chains, each typically consisting of around 440 amino acids (50 kDa).^[^ ^38^ ^]^ In Figure 2c, the high‐resolution N 1s spectrum peaks at 398.4 eV, which can be assigned to the C─N/C═N bond at the imidazole ring.^[^ ^39^ ^]^ A small peak at 399.8 eV is also observed for the IDEZ sample and can be related to Zn‐N interactions.^[^ ^40^ ^]^ For the samples with the antibodies immobilized on the ZIF‐8 surface, the most intense N1s peak is related to the presence of C─N/C═N, which can be attributed to the polypeptide chains from the antibodies.

Furthermore, the occurrence of a peak at 401.4 eV can be related to the amino groups (─NH_2_) from the antibodies, highlighting their successful immobilization on the ZIF‐8 surface. The high‐resolution O 1s spectra for the IDEZ sample, Figure 2d, indicate that most of the oxygen is in the form of hydroxyl species or adsorbed oxygen (532.1 eV).^[^ ^41^ ^]^ In addition, a small peak at 530.8 eV is consistent with the presence of carbonates. After the antibody immobilization, the spectra indicated an intense peak at 531.3 eV and a minor contribution at 530.1 eV. The high‐intensity contribution can be related to carbonates that accumulate on surfaces. The less intense peak can be associated with the Zn‐O bond due to uncoordinated zinc ions. The Zn 2p spectrum of the IDEZ sample exhibits a pronounced spin‐orbit splitting (Figure 2e), with two distinct peaks at binding energies of 1021 eV and 1044 eV, corresponding to Zn 2p_3/2_ and Zn 2p_1/2_, respectively (Δ_metal_ = 23 eV).^[^ ^42^ ^]^ These peaks are characteristic of ZIF‐8, indicating tetrahedrally coordinated Zn and zinc ions with low coordination numbers. Following the immobilization of antibodies, a slight shift (1 eV) of Zn 2p spectra level to lower energy is observed.

Two peaks appear at 1022 eV and 1045 eV, attributed to antibodies binding to the open Zn metal sites. Thus, we can infer that the ZIF‐8 surface is rich in uncoordinated Zn ions, which can act as antibody interaction sites (Figure 2f). Another potential mode of interaction could arise from the presence of ─OH ions and carbonates on the ZIF‐8 surface, which may interact with amino groups from the antibodies, as schematically illustrated in Figure 2g.

### 2.3 Biosensor Linearity

Electrochemical impedance spectroscopy (EIS) analyses were performed to obtain the standard curve for each of the biomarkers. Recombinant proteins from the biomarkers were utilized in different concentrations (Table S1, Supporting Information). Before the biosensor analysis, a Western Blot (WB) evaluation was developed to determine if the antibodies could detect the respective recombinant proteins and saliva proteins (Figure S4, Supporting Information). In the Bode plot (Figure S5a—c, Supporting Information), the characteristic negative slope observed for all cases is indicative of capacitive behavior. For the CSTB and LTA4H biomarkers (Figure S5a,b, Supporting Information), after the antibody interacts with the protein, as the concentration of CSTB and LTA4H increases, the impedance decreases in this region between 1 and 0.025 Hz. Through the phase angle (Figure S5d,e, Supporting Information), it is possible to observe the capacitive behavior is preserved from 10^5^ to 10^3^ Hz, whereas ionic charge transport is predominant at lower frequencies. The Nyquist plot indicates a decrease in the diameter of the semicircle with an increase in the concentration of CSTB (Figure S5g, Supporting Information) and LTA4H (Figure S5h, Supporting Information). The semicircle is associated with charge transfer resistance,^[^ ^43^ ^]^ while the straight tail is associated with ion diffusion. Therefore, increased CSTB and LTA4H concentrations indicate lower charge transfer resistance.

On the other hand, for the COL6A1 biomarker, it is possible to observe an increase in impedance directly proportional to the increase in protein concentration (Figure S5c, Supporting Information). Unlike the LTA4H and CSTB biomarkers, the Nyquist plot for COL6A1 (Figure S5i, Supporting Information) shows higher charge transfer resistance with increasing protein concentration. This occurs because collagen is a complex polymer with a molecular weight of approximately 108 kDa, capable of forming a microfibrillar mesh. The higher the concentration of this protein, the larger the polymeric network formed and, consequently, the impedance. Furthermore, it is known that the impedance measured in dry and wet collagen differs significantly. The high conductivity observed in wet collagen thin films is linked to the presence of water molecules, which interact with the hydroxyl groups in collagen.^[^ ^44^, ^45^ ^]^ In contrast, when collagen is in a dry state, its conductivity is much lower, resulting in increased impedance.^[^ ^44^ ^]^

The optimal detection frequency was determined as 0.4 Hz, based on a systematic analysis of the impedance spectra obtained during calibration experiments with recombinant proteins at varying concentrations for each of the three biomarkers (CSTB, LTA4H, and COL6A1). For each target, we performed measurements considering a broad frequency range (10 MHz to 25 mHz) and identified the frequency region where impedance changes were most responsive to concentration variations, thereby defining the linear detection range. As shown in the Bode plots (Figure S5, Supporting Information), each biomarker exhibited a distinct impedance profile: for CSTB (Figure S5a, Supporting Information) and LTA4H (Figure S5b, Supporting Information), impedance values decreased with increasing biomarker concentration, while for COL6A1 (Figure S5c, Supporting Information), impedance increased proportionally with concentration. These variations were particularly noticeable in the low‐frequency range between 1 and 0.025 Hz. Within this region, 0.4 Hz consistently produced pronounced and concentration‐dependent impedance changes across all three biomarkers. To evaluate linearity, we calculated the coefficient of determination (R^2^), with values close to 1 indicating stronger correlation. While lower frequencies (0.5–0.025 Hz) often exhibited *R* ^2^ values close to 1, we selected 0.4 Hz to optimize measurement time, as higher frequencies allow faster data acquisition without compromising sensitivity or accuracy.

### 2.4 Saliva Sample Analysis by EIS

To enable analysis of the target biomarkers, saliva samples from OSCC patients with active lesions were deposited onto the active area of the biosensors in a controlled and reproducible manner, ensuring efficient and specific interaction with the functionalized detection layer.

From the EIS data, the biosensor showed better linearity at 0.4 Hz (Figure S6a—c, Supporting Information), where ionic charge transport is predominant over electronic. Such linearity can be fairly represented considering the capacitance (*C*), which reduces interference from non‐relevant solution resistances and provides a clearer representation of interfacial events that drive the biosensor's response. These values can be obtained from the imaginary impedance (Zi). Since Zi is related to capacitance (*C*),^[^ ^46^ ^]^ it was calculated using Equation (2):

(2) $$C=-\frac{1}{\left(2\pi \mathrm{fZi}\right)}$$

*C* corresponds to capacitance (*F*), *f* to frequency (Hz), and *Z_i_* to imaginary impedance (*Ω*). Figure S6a (Supporting Information) shows the standard curve for the CSTB biomarker, which ranged from 31.25 to 4000 ng mL^−1^ with a coefficient of determination (*R* ^2^) of 0.9939. For the LTA4H biomarker, the linearity range was from 31.25 to 1000 ng mL^−1^ with an R^2^ of 0.9952 (Figure S6b, Supporting Information). The COL6A1 biomarker, in turn, presented a linear range between 0.97 to 250 ng mL^−1^ with an *R* ^2^ of 0.9931 (Figure S6c, Supporting Information). A control test analyzing the impedance without the antibody modification showed that the biosensor could not detect the biomarkers (Figure S6d,e, Supporting Information).

**Figure 3:**
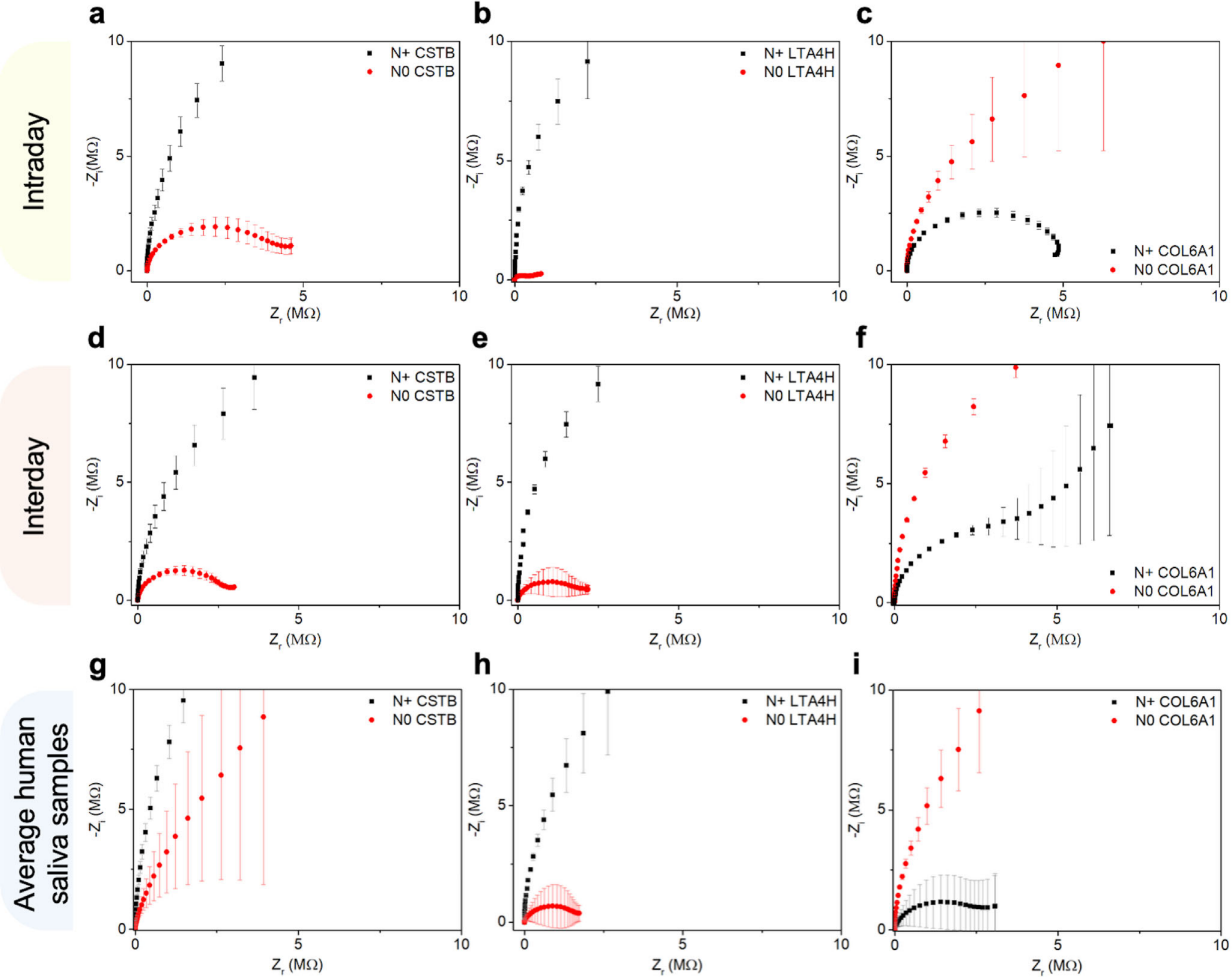
The specificity and reproducibility of the biosensor were evaluated using Nyquist plots for N0 and N+ saliva samples. Despite the complexity of the saliva matrix, the biosensor successfully detected the target biomarkers, demonstrating its specificity. Variability analysis was performed on the same day (intraday) for a) CSTB, b) LTA4H, and c) COL6A biomarkers in N0 and N+ saliva samples. Additionally, interday variability was analyzed for the same biomarkers—d) CSTB, e) LTA4H, and f) COL6A1—across different days. The results indicate that variability in biomarker detection remained consistent, enabling clear differentiation between the N0 and N+ groups in both intraday and interday analyses. All experiments were performed in triplicate. Average impedance values for thirty N0 and N+ samples, represented by Nyquist plot, for g) CSTB, h) LTA4H, and i) COL6A1 showed the separation of distinct prognostic groups.

To verify the specificity and reproducibility of the biosensor, saliva samples from patients with and without lymph node metastasis, referred to as N+ and N0, respectively, were analyzed. Despite the complexity of the saliva matrix, the biosensor successfully detected the target biomarkers, demonstrating its specificity. The reproducibility analysis was conducted for N0 and N+ samples evaluated by EIS on the same day (intraday) or on different days (interday). In **Figure** **3** it can be observed that the variability in the detection of the biomarkers CSTB (Figure 3a,d), LTA4H (Figure 3b,e), and COL6A1 (Figure 3c,f) did not show significant changes, allowing differentiation between the N0 and N+ groups in both situations, mainly through the Nyquist plot. For the CSTB and LTA4H biomarkers, it can be inferred from the Nyquist plots that the N+ samples exhibit behavior like a capacitor. In contrast, the N0 sample shows a smaller semicircle diameter, indicating lower charge transfer resistance than the N+ samples. For the COL6A1 biomarker, the opposite effect is observed, with the N+ sample showing lower charge transfer resistance than the N0 samples.

To ensure the transfer selectivity of the biosensor, or its ability to specifically detect the target biomarker in the presence of many other salivary components, we adopted different strategies. First, antibody specificity was confirmed via Western blot using both recombinant proteins and saliva samples, with clear detection of target bands for CSTB, LTA4H, and COL6A1 (Figure S4, Supporting Information), demonstrating selective antigen recognition. Second, each biosensor was functionalized with a single antibody, and measurements were performed individually for each biomarker, minimizing cross‐binding or signal interference. Furthermore, control devices without antibody functionalization did not produce a significant electrochemical response (Figure S6d,e, Supporting Information), confirming that signal generation depends specifically on antigen‐antibody binding. Besides that, recovery experiments in spiked saliva samples were performed to determine the interference from non‐target salivary components. To determine the recovery rate of the biosensor for CSTB, LTA4H, and COL6A1, 10 ng of the respective recombinant proteins were added to a saliva sample. The recovery rate of the proteins introduced into the saliva matrix was calculated using the capacitance value at 0.4 Hz from the linearity curve equation obtained for each biomarker. **Table** **1** shows the values recovered for the proteins of interest. The recovery rate was above 90%, with a relative standard deviation (RSD) of less than 8.1% for all samples, indicating good specificity of the biosensor for each biomarker.

**Table 1:** Recovery study of the ZIF‐8 biosensor was performed by adding standard solutions of the respective recombinant protein to a saliva sample (All samples were analyzed in triplicates).

Biomarker	Added [ng]	Mean measured [ng]	Mean recovery [%]	RSD [%]
CSTB	10	9.3	93.1	8.1
LTA4H	10	9.0	90.3	2.0
COL6A1	10	10.7	107.7	5.7

For accurate OSCC prognosis monitoring using biosensors, the use of individual saliva samples is essential, as the system is specifically designed for personalized data acquisition and calibrated to detect and quantify unique prognostic biomarkers with potential clinical relevance. In this way, thirty N0 and N+ samples were selected for EIS analysis based on TNM clinical or pathological staging information, which provides data on tumor size and appearance (T), spread to nearby lymph nodes (N), and occurrence of distant metastasis (M). EIS analyses showed that the biosensors were able to detect the presence of biomarkers in biological samples and differentiate them according to prognostic classification between groups of OSCC patients with and without lymph node metastasis. As previously observed, for the CSTB and LTA4H biomarkers, the N+ samples exhibited more capacitive behavior, with a larger semicircle diameter observed in the Nyquist plot (Figure 3g,h). For the N0 samples, the semicircle diameter was smaller, indicating a decrease in charge transfer resistance, which is directly related to the concentration of CSTB and LTA4H in these samples.

The interaction between the antibody and antigen alone can generate changes in the electrical properties of the biosensor. Considering the properties of each protein, CSTB and LTA4H have a higher percentage of ionic and polar amino acids compared to COL6A1. Thus, an increase in CSTB and LTA4H concentration may further contribute to charge transport due to the presence of negatively or positively charged groups (zwitterionic forms) in the protein structure. Thus, a higher concentration of these proteins results in reduced impedance. The change in impedance values due to an increase in ions also explains why this variation is observed at low frequencies (0.4 Hz), which is related to ionic charge transport.

For COL6A1, N0 samples with higher COL6A1 concentration exhibited more capacitive behavior (Figure 3i). This may occur because COL6A1 can form polymeric networks that increase impedance. However, its structure also contains lysine, arginine, aspartic acid, and glutamate amino acids that are charged at neutral pH. Therefore, low concentrations of this protein may contribute to charge transfer due to the presence of these amino acids in their zwitterionic form. However, when collagen concentration increases, the polymeric network formed acts as a barrier to charge transfer, increasing impedance.

Using the linear equations and the capacitance values at 0.4 Hz, it was possible to estimate the concentration range of the biomarkers present in the saliva of N0 and N+ patients. In both cases, the average concentration in N+ samples was lower (Figure S6f–h, Supporting Information) compared to N0 samples and a tendency in the separation of prognostic groups can be observed. It is important to note that the response obtained, with lower concentrations of the prognostic biomarkers in N+ samples, is consistent with previously observed results from target‐based proteomics analyses.^[^ ^19^ ^]^ The Bode and phase angle plots are shown in Figure S7 (Supporting Information).

Additionally, the limit of detection (LOD) and limit of quantification (LOQ) were determined for each biomarker. The LOD and LOQ were calculated using the standard deviation of the intercepts and the average slopes from the analytical calibration curves. Specifically, the LOD was defined as 3.3 times the standard deviation of the regression equation divided by the slope,^[^ ^47^ ^]^ while the LOQ was defined as 10 times the standard deviation divided by the slope.^[^ ^48^ ^]^ The calculated values are presented in **Table** **2**. The LOD values are as low as 0.35, 0.24, and 0.30 ng mL^−1^ for CSTB, LTA4H, and COL6A1, respectively, indicating a good sensitivity of the biosensor. LTA4H quantification achieved the lowest LOD and LOQ values, among the three analyzed biomarkers.

**Table 2:** Detection (LOD) and quantification limits (LOQ) parameters of the ZIF‐8 biosensors (All samples were analyzed in triplicates).

Biomarker	LOD [ng mL^−1^]	LOQ [ng mL^−1^]	SD	*R* ^2^
CSTB	0.3495	1.0590	0.0939	0.9939
LTA4H	0.2419	0.7329	0.1169	0.9952
COL6A1	0.3044	0.9224	0.1358	0.9931

### 2.5 Supervised Machine Learning Analysis

Machine learning (ML) analysis was applied to the biosensor results to achieve differentiation between the sample groups. Four experimental scenarios considering each biomarker individually (LTA4H, COL6A1, and CSTB) as well as their combined effect were analyzed using ML approaches. These analyses identified the presence of some outliers, and their removal was necessary to achieve more robust results (Figures S8–S11 and Figure S6f–h, Supporting Information). We built 17 different supervised ML models, once the application of appropriate ML algorithms improves the accuracy and efficiency of biosensors.^[^ ^49^, ^50^ ^]^ It is important to emphasize that no model can be considered optimal without an intense evaluation. Furthermore, the results were analyzed with and without hyperparameter optimization, leading to a total of 34 supervised classification models, with rankings based on the balanced accuracy value, weighted AUC‐ROC, and weighted F1‐score. Results before and after optimization were considered and the best classifiers were selected for the individual biomarkers, as well as in combination (Figures S12–S15, Supporting Information). Models exhibiting a train‐test performance gap exceeding 0.2—calculated as the arithmetic mean of performance deltas across accuracy, F1‐score, and AUC‐ROC metrics: (Δaccuracy + ΔF1‐score + ΔAUC‐ROC)/3—were excluded from consideration, with selection proceeding to the next‐ranked candidate. This generalization threshold criterion ensures model selection prioritizes robust performance on unseen data over training set optimization, thereby favoring models with superior real‐world applicability and reduced overfitting propensity.

**Figure 4:**
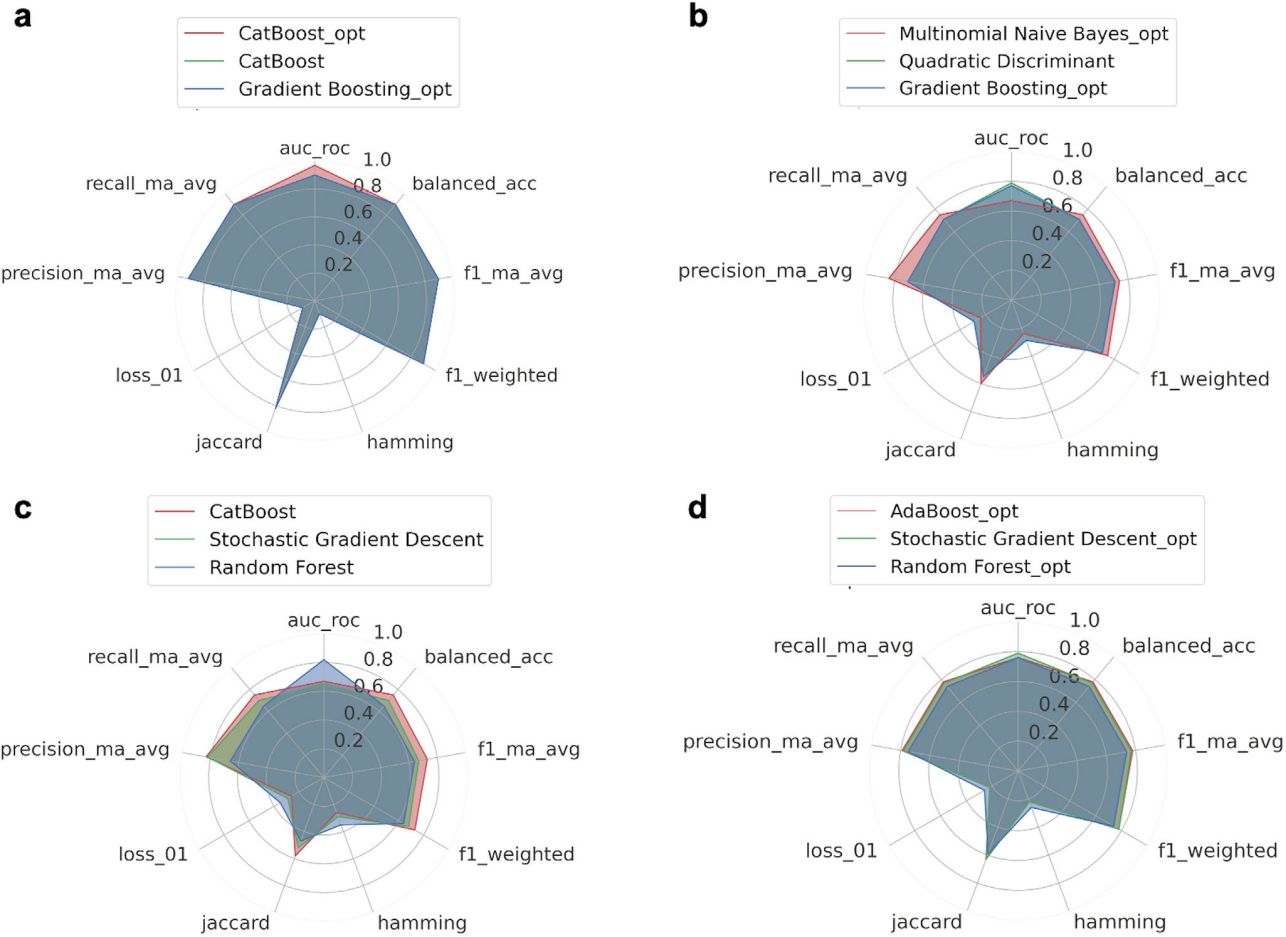
ML supervised models. Radar plot showing the three best classifiers for a) LTA4H, b) COL6A1, c) CSTB, and d) combined analysis, with rankings based on the balanced accuracy value, weighted AUC‐ROC, and weighted F1‐score. ML results indicated that LTA4H alone can better distinguish the prognostic groups with higher accuracy. Considering the combined analysis, a good accuracy was also obtained, emphasizing the significance of this model.

For the LTA4H biomarker, the radar plot indicated that CatBoost (CB) optimized, CB not optimized, and Gradient Boosting (GB) optimized models achieved the highest rankings (**Figure** **4**a). The individual ML analysis of the LTA4H biomarker generated confusion matrices to evaluate true and false positives across all classification models for both the training and testing groups, with and without hyperparameter optimization (Figures S16–S19, Supporting Information). To assess the performance of the best‐performing ML model, a normalized confusion matrix was generated with row‐wise normalization. The three best models achieved a 100% true positive rate for N+ samples and an 80% true positive rate for N0 samples in classifying the LTA4H biomarker, demonstrating excellent classification performance. However, the false positive rate of 20% for N0 samples suggests potential areas for refinement, suggesting that further analysis with a larger dataset could improve model accuracy. One of the approaches considered to evaluate the overall accuracy of the ML model is by examining the area under the curve (AUC) from the receiver operating characteristic (ROC) curve. A higher AUC value reflects a more effective classifier, providing a comprehensive overview of the model's performance. The AUC ranges from 0 to 1, where a value of 0 signifies a completely inaccurate test and a value of 1 indicates a perfectly accurate test. Generally, an AUC of 0.5 indicates that the model's ability to distinguish between positive and negative classes is no better than random guessing. AUC values between 0.7 and 0.8 are considered acceptable, between 0.8 and 0.9 are deemed excellent, and values above 0.9 are regarded as outstanding.^[^ ^51^ ^]^ The AUC values for the top three classifiers (CB tuned, CB, and GB tuned) were 97%, 90%, and 90%, respectively, indicating their ability to accurately distinguish between metastatic and non‐metastatic OSCC patients (Figures S20 and S21, Supporting Information). Although a higher cohort is necessary to validate these findings, these exceptional results suggest that LTA4H could be a highly effective prognostic biomarker for OSCC.

For COL6A1 and CSTB biomarkers individually evaluated, the accuracy was notably lower when compared to LTA4H. For COL6A1, the three best classifiers, shown in the radar plot (Figure 4b), are the optimized models Multinomial Naive Bayes (MNB), Quadratic Discriminant (QD), and GB. On the other hand, the three best classifiers for CSTB are the non‐optimized models CB, Stochastic Gradient Descent (SGD), and Random Forest (RF), Figure 4c. Regarding the confusion matrix analysis for COL6A1 (Figures S22–S25, Supporting Information) the best‐performing classifier, MNB, achieved 50% true positives for N+ and 100% for N0 samples, while QD resulted in 50% for N+ and 66% for N0, and GB reached 75% for N+ and 66% for N0 samples. The AUC‐ROC curve (Figures S26 and S27, Supporting Information) further highlighted MNB as the best classifier, with an overall accuracy of 67%.

For CSTB, regarding the confusion matrix analysis (Figures S28–S31, Supporting Information), CB revealed 50% true positives for N+ and 100% for N0 samples, while SGD indicated 100% true positives for N+ and 40% for N0 samples, and RF presented 50% true positives for N+ and 80% for N0 samples. The AUC‐ROC curve (Figures S32 and S33, Supporting Information) for the CB model was 67%. These lower outcomes suggest that LTA4H is a more reliable and accurate biomarker for OSCC prognosis than CSTB or COL6A1.

Besides the individual analyses, a combined evaluation using data from three biomarkers revealed that the optimized models AdaBoost (AB), SGD, and RF were the three best classifiers (Figure 4d). A confusion matrix was generated to closely examine true and false positives for all detection models in the combined analysis (Figures S34–S37, Supporting Information). The AB model achieved a 68.8% true positive rate for N+ samples and an 86.7% true positive rate for N0 samples, while the SGD model indicated a 75% true positive rate for N+ samples and an 80% true positive rate for N0 samples, and RF resulted in a 68.8% true positive rate for N+ samples and an 80% true positive rate for N0 samples. These models indicated a reasonably effective classification for prognostic groups considering the three biomarkers. However, the false positive rates higher than 30% for N+ samples in AB suggest areas where the model could be further refined. Based on the AUC results (Figures S38 and S39, Supporting Information), the AB model emerged as the top‐performer with an AUC of 0.76, followed closely by the SGD model, which achieved an AUC of 0.79, and RF with an AUC of 0.76. Therefore, this suggests that the AB model shows a 76% probability of accurately distinguishing between metastatic and non‐metastatic OSCC patients. Although an accuracy of 76% indicates the need for further improvement, it is important to highlight that no established biomarker model currently exists for OSCC prognosis, emphasizing the significance of this model. Future studies should focus on increasing both the size and diversity of the sample cohort, incorporating a more representative population that reflects the heterogeneity of oral cancer in terms of tumor stage, lesion site, patient demographics, and comorbidities. Such enhancements are expected to improve the model's accuracy and clinical robustness. The moderate prediction probabilities observed in both LTA4H and combined model scenarios, characterized by well‐calibrated confidence estimates rather than overconfident predictions, indicate that model performance is primarily constrained by sample size limitations rather than overfitting pathologies. This suggests the models have achieved appropriate bias‐variance trade‐offs given the available data constraints. If model performance equals or exceeds clinician prognostic accuracy or established clinical decision‐making frameworks, the approach may demonstrate clinical value through enhanced consistency, reduced processing time, and improved scalability factors that contribute to healthcare delivery optimization beyond pure prognostic accuracy metrics.

As mentioned previously, the individual analysis of LTA4H demonstrated superior classification performance (Figure 4a) compared to both CSTB and COL6A1 individually, as well as to the combined analysis of all three biomarkers. Additionally, the results obtained correlate with our previous mass spectrometry data, as the LTA4H protein proved to be the most accurate signature for determining prognosis at the protein level.^[^ ^19^ ^]^ Thus, EIS analyses optimized through ML approaches indicate the potential application of the electrochemical biosensor for detecting these biomarkers in non‐invasive biological samples, making it a promising and accessible tool for oral cancer prognosis.

### 2.6 Model Interpretability and Explainability

This approach enabled the identification of the most influential predictor attributes in each case. Methods like SHAP and LIME demystify machine learning models by quantifying feature importance in both individual predictions and overall model behavior. Their insights are invaluable for guiding future biosensor optimization—helping researchers prioritize, refine, or eliminate features to enhance performance, interpretability, and trust in biosensor‐driven clinical or environmental applications.

Optimal models from each experimental scenario were subjected to comprehensive interpretability analysis, with quantitative performance metrics detailed in Tables S3–S6 (Supporting Information). Model explainability analyses, including feature importance rankings and local explanation visualizations for the selected algorithms, are presented in Figures S40–S43 (Supporting Information). The best‐performing hyperparameter‐tuned in the combined scenario (Figure S43, Supporting Information)—AdaBoost (mean gap = 0.07)—demonstrated equivalent F1‐scores and balanced accuracy metrics of 0.78. Corresponding AUC‐ROC value was 0.76, indicating some discriminative performance. In Figure S43a (Supporting Information) a SHAP summary plot is used to visualize feature importance and how each feature contributes to the model output. The y‐axis contains all feature names ranked by their average impact on the combined scenario model's prediction. On the other hand, the X‐Axis contains the SHAP values which represent the impact of each feature value on the model output: positive (N+) or negative (N0). Positive values contribute toward the positive class, therefore low “[all] (ng/ml)” values push the prediction toward N+ class, being the contrary also true. Considering the spread wide across the X‐axis of the feature “[all] (ng/ml)” it has the most impact on the model output followed by “COL6A1” and “C1 at 0.4 Hz.” The feature “CSTB” which had the lowest performance among the experimental scenarios also placed in the last position for model impact. Another interesting visualization is the SHAP waterfall plot for a single test N+ sample (Figure S43b, Supporting Information). It explains how each feature contributes to pushing the model prediction from the baseline (average model output) to the final prediction. In the mentioned case, the average prediction across all samples, i.e., the base value is *E*[*f*(*X*)] = 0.44. The feature “[all] (ng/ml)” contributes +0.39 making a strong positive contribution with a low value (–0.176). As expected from the previous finding, it means low values of “[all] (ng/ml)” are associated with a higher probability of the positive class in your model, i.e., N+. Finally, the combination of three features with low values (“[all] (ng/ml),” “logC1,” “C1@0.4Hz”) pushed the prediction to *f*(*x*) = 1 (the final prediction for this sample), strongly supporting the positive class. Other features had no effect on this sample. The LIME method was also employed to address the interpretation of the combined scenario selected model. The first two parts of Figure S43c (Supporting Information) represent prediction probabilities for a single N0 and N+ sample, respectively, in addition to the sample values for each feature. The last part of the Figure S43c (Supporting Information) contains two LIME barplots. In these plots, each feature in the y‐axis is ranked by their impact predicting the sample outcome, either positive (green) increasing the prediction probability for class N+/N0 or negative (red) decreasing the probability for class N+/N0. In addition to feature importance, each feature condition reflects a binned range in which the value for this sample falls into. The left one explains a single prediction for class N+ and the right one for class N0. In the left panel, the feature “[all] (ng/ml)” with values ≤ ‐0.17 had a strong positive impact on class N+ supporting it. However, the binary feature “COL6A1” ≤ 0.00 had a negative impact, slightly pushing prediction away from class N+. The final interpretation from this LIME plot confirms what SHAP suggested, in which low values of “[all] (ng/ml)” are the main driver of this sample being classified as positive, while COL6A1 is the only feature opposing the decision, but weakly. The local explanation for class N0 (right panel) demonstrates analogous interpretability patterns, with the key distinction being the positive‐valued concentration ranges bounded by defined lower and upper thresholds for the “[all] (ng/ml)” measurements. Feature importance rankings and directional impacts remain consistent across both class predictions, with variability restricted to the specific concentration value ranges corresponding to each diagnostic category. This interpretability assessment identifies the most discriminative biomarkers within the feature space and establishes their relative predictive utility for the diagnostic task. The feature importance analysis provides actionable insights for biosensor design optimization by quantifying the contribution of individual protein targets to classification accuracy. These findings can inform future sensor development strategies through prioritization of high‐impact biomarkers, potentially enabling sensor miniaturization and cost reduction while maintaining prognostic performance. The integration of interpretability metrics with performance evaluation creates a comprehensive analytical framework that bridges machine learning model behavior with practical biosensor engineering considerations, facilitating data‐driven optimization of future prognostic and diagnostic platforms.

### 2.7 Perspectives and Potential Challenges for Point‐of‐Care Implementation

One of the key advantages of the proposed electrochemical biosensor platform is its potential for low‐cost testing, particularly when compared to conventional methods such as ELISA or mass spectrometry analysis, which require expensive reagents, instrumentation, and specialized personnel. Once optimized for point‐of‐care (PoC) use, the biosensor could be fabricated using scalable and cost‐effective processes, with each test requiring only small volumes of reagents and sample. Additionally, the use of EIS allows for label‐free detection, further reducing operational costs.

While not yet ready for PoC implementation, the technology demonstrates a critical proof of concept for noninvasive, multi‐biomarker prognostic evaluation, paving the way for future clinical translation. In this way, to translate the proposed biosensor into a clinically relevant PoC platform, some key considerations must be addressed. Currently, the biosensor relies on a benchtop impedance analyzer that performs a broad frequency sweep to identify the optimal detection frequency. For PoC applications, this approach would need to be simplified. Specifically, a portable system should be designed to perform impedance measurements at a fixed, optimized frequency range (e.g., 0.4 Hz), as determined from our calibration studies. This would enable the design of a compact, low‐power electronic readout system, reducing complexity and response time. Regarding the use of saliva, additional studies should be conducted to evaluate the biosensor's performance using native (uncentrifuged) saliva. This would allow assessment of whether cellular debris or other particulates present in raw samples interfere with the biosensor response, once it could lead to electrode fouling or non‐specific signal shifts. It is also important to note that although the biosensors were developed to detect multiple biomarkers, it is not a multiplexed detection. In this way, implementing multiplexed detection would enable simultaneous biomarker readouts. However, this would lead to a potential challenge in signal deconvolution strategies, increasing complexity in both hardware and signal processing. Further, incorporating real‐time data acquisition, noise filtering, and classification (e.g., ML model) requires embedded computation or wireless transfer to a mobile device/cloud. In summary, while the current biosensor provides a strong foundation for prognostic evaluation in OSCC, the path to a fully integrated PoC system involves coordinated advancements in electronics, sample preparation, and data science.

## 3 Conclusion

In summary, a sensitive electrochemical biosensor was successfully developed to detect and quantify CSTB, LTA4H, and COL6A1 biomarkers in noninvasive saliva samples from OSCC patients, with and without lymph node metastasis. The detection was based on the antigen‐antibody immunoreaction between each biomarker antibody and the specific analyte present in the biofluid. When the transducer interacts with the target analyte, a shift in capacitance was observed, indicating that N0 samples showed a higher concentration of the biomarkers when compared to N+ samples. Furthermore, the inter‐ and intraday analysis as well as the recovery evaluation of the biosensor indicated good accuracy, specificity, and reproducibility, with LOD values as low as 0.35, 0.24, and 0.30 ng mL^−1^ for CSTB, LTA4H and COL6A1, respectively. Supervised machine learning analysis for the individual and combined biomarkers indicated that LTA4H can differentiate the prognostic groups with higher accuracy. Furthermore, the combined effect of the biomarkers indicated 76% accuracy in the AUC‐ROC curve for the AdaBoost model. While LTA4H alone yielded the best classification results, the combined effect of multiple biomarkers also demonstrated strong accuracy. Although not yet ready for point‐of‐care implementation, once some key considerations must be addressed—such as fixed‐frequency impedance readout, native saliva testing, multiplexing capabilities, and integrated data processing—no established biomarker model for OSCC prognosis currently exists, highlighting the relevance of this approach. Expanding the sample cohort's diversity and size in future studies could further validate and enhance the model's accuracy, offering a more robust and reliable tool for clinical application. Consequently, EIS analyses combined with ML approaches suggest that this biosensor could be highly effective in detecting OSCC biomarkers in non‐invasive biological samples, making it a promising tool for real‐time monitoring of OSCC progression, potentially improving therapeutic decisions and patient outcomes.

## 4 Experimental Section

### Chemicals and Reagents

All chemicals were analytical grade obtained from Sigma‐Aldrich or Merck unless otherwise noted. Kanamycin Sulfate (C_18_H_38_N_4_O_15_S, 95–100%) and ethylenedinitrilotetraacetic acid disodium salt dihydrate (EDTA, 0.5 m pH 8) were purchased from Gibco (Waltham, MA, USA). Imidazole (C_3_H_4_N_2_, 99%) was purchased from Oakwood Chemical (West Columbia, SC, USA). Sodium hydroxide (NaOH, 97%) was purchased from Synth (Diadema, SP, Brazil). Isopropyl‐β‐d‐thiogalactopyranoside (IPTG, 99%) was purchased from Promega (Madison, WI, USA). Sulfuric acid (H_2_SO_4_, 95–98%) was purchased from J. T. Baker (Phillipsburg, NJ, USA). Tris(hydroxymethyl)‐aminomethane (Tris, NH_2_C(CH_2_OH)_3_, 99.8%) was purchased from Affymetrix (Santa Clara, CA, USA). The cocktail Protease Inhibitor was purchased by Roche (Basel, Switzerland). The human recombinant proteins LTA4H and COL6A1 were purchased from R&D Systems (Minneapolis, MN, USA) and Novus Biologicals (Centennial, CO, USA), respectively. The LTA4H (HPA008399), CSTB (HPA017380), and COL6A1 (HPA019142) antibodies were purchased from Sigma‐Aldrich. The HiSeq 2500 platform was purchased from Illumina (San Diego, CA, USA). Vibracell VCX 500 apparatus was obtained from Sonics & Materials, Inc., Newtown, CT, USA. HisTrap chelating column was purchased from GE Healthcare (Chicago, IL, USA).

### Amplification and Cloning of Recombinant Human CSTB Gene

The purified recombinant human CSTB protein (UniProt P04080, M_1_‐F_98_) was obtained through amplification and cloning procedures. The CSTB gene was amplified by polymerase chain reaction (PCR) using a cDNA library derived from HEK‐293 cells as a template. The following oligonucleotide primers were employed: forward 5′ CGCGGATCCGCGATGATGTGCGGGGCGCCCTCCG 3′ and reverse 5′ CCGCTCGAGCGGTCAGAAATAGGTCAGCTCATC. The recombinant construct was designed to express CSTB fused to an N‐terminal polyhistidine tag (CSTB‐His). The PCR product was subcloned into the bacterial expression vector pET28a(+) using BamHI and XhoI restriction enzymes. The efficacy of the amplification and cloning procedures was evaluated by agarose gel electrophoresis. The integrity and fidelity of the nucleotide sequence of the pET28a+/CSTB‐His construct were confirmed by next‐generation sequencing using the HiSeq 2500 platform.

### Expression and Purification of Recombinant CSTB Protein

The CSTB‐His construct was expressed in *Escherichia coli Origami 2 (DE3)* and the recombinant protein was purified by affinity chromatography using Ni Sepharose resin. Chemically competent *E. coli Origami 2 (DE3)* cells were transformed with the pET28a(+) expression vector containing the CSTB‐His construct, as detailed by Calvete et al.^[^ ^52^ ^]^ Transformants were cultured in Luria‐Bertani (LB) medium supplemented with kanamycin (1% w/v) at 37 °C until the optical density at 600 nm (OD600) reached 0.6–0.8. Protein expression was induced by adding isopropyl β‐D‐1‐thiogalactopyranoside (IPTG) to a final concentration of 0.5 mm, followed by incubation at 37 °C for 2 h with orbital shaking at 200 rpm.

Post‐expression, bacterial cells from 1 L of culture were harvested by centrifugation and resuspended in 20 mL of buffer A (50 mm Tris/HCl pH 7.5, 150 mm NaCl, 2 mm CaCl_2_, 20 mm imidazole) supplemented with 1 mm phenylmethylsulfonyl fluoride (PMSF). Cell lysis was performed by sonication using a Vibracell VCX 500 apparatus.

The purification procedure was adapted from Trino et al.^[^ ^28^ ^]^ The bacterial lysate was centrifuged at 14 000 *g* for 30 min at 4 °C. The resulting supernatant was applied to a 1 mL HisTrap Chelating column pre‐loaded with nickel and equilibrated with buffer A. Protein elution was achieved using a linear imidazole gradient (0.02–0.5 m) at a flow rate of 1 mL min^−1^.

Eluted fractions were analyzed by 15% sodium dodecyl sulfate‐polyacrylamide gel electrophoresis (SDS‐PAGE) under denaturing conditions. Fractions containing the protein of interest were pooled and subjected to buffer exchange into PBS pH 7.2 using an Amicon device with a 3 kDa molecular weight cut‐off. The concentration of the purified protein was determined spectrophotometrically using a NanoDrop 2000/2000c instrument (Thermo Scientific, Waltham, MA, USA).

### Fabrication of Interdigitated Electrodes (IDEs) Modified with ZIF‐8

The IDEs fabrication was performed as previously reported.^[^ ^28^ ^]^ Briefly, photolithography was used to create the desired pattern to deposit Cr/Au (20/20 nm) upon the silicon wafers (100). Each IDE has 12 mm × 7 mm in size, 60 electrode arrays confined to an active area of ≈15 mm^2^, a channel length of 10 µm, and a total channel width‐length (W/L) ratio of 50 000. After cleaning, the IDEs were hydroxylated and immediately immersed in a fresh mixture of Zn(NO_3_)_2_ (25 mm) and 2‐methylimidazole (2‐MeIm, 50 mm) for 6 h under stirring and at room temperature. Finally, the ZIF‐8 thin films were washed with methanol, dried with N_2_ flow, and stored under vacuum.

### Antibody Deposition

The functionalization of the modified IDEs was performed using the antibodies from CSTB, LTA4H, or COL6A1 (1:500 in 20 µL) for 2 hours under 37 °C. Then, after a washing process to eliminate the weakly bound or unbound antibodies, the IDEs were immediately used to deposit either the recombinant protein or the saliva from OSCC patients. For that, 20 µL of saliva or different recombinant protein concentrations were left on the functionalized electrodes for 30 minutes, followed by a washing step with water and drying with N_2_ flow.

### Sample Characterization

The ZIF‐8 thin films were morphologically characterized using field emission scanning electron microscopy (FEG‐SEM, Inspect F50 from FEI). The crystalline structure of the ZIF‐8 thin films was determined via X‐ray synchrotron radiation at the XRD2 beamline at the Brazilian Synchrotron Light Laboratory (LNLS), with an incident wavelength (*λ*) of 1.54979 Å. Diffractograms were collected in the range of 5° to 20° (2θ), with a step of 0.02°, time per step of 50.0 s°^−1^, and incident angle of 0.20° using a Cu‐anode (*λ* = 0.154 nm). Atomic force microscopy (AFM, Park NX10 with an NX‐AFM controller) operated in peak force tapping mode using a Silicon tip from Nano World to analyze the surface roughness and film thickness of the modified IDEs, utilizing Gwyddion software. The AFM tips have a nominal resonant frequency of 75 kHz and a force constant of 2.8 N m^−1^. X‐ray photoelectron spectroscopy (XPS) was acquired using a Thermo Scientific K𝛼 micro‐focused monochromatized source with a resolution of 0.1 eV, pass energy of 50 eV, spot size of 300 µm, and 20 scans. The survey spectra were collected over an energy range of 0–1200 eV. Data analysis was conducted using CasaXPS software (Casa Software, Ltd.). All peak positions were calibrated relative to the binding energy of adventitious C 1s at 284.8 eV.

### Human Saliva Collection and Preparation

This study was approved by the Ethics Review Board of the Cancer Institute of São Paulo (ICESP), São Paulo, SP, Brazil, and Plataforma Brasil through protocol CAAE 83729224.0.0000.5418. All participants provided written informed consent. The study was conducted following the Declaration of Helsinki and was performed following the Strengthening the Reporting of Observational Studies in Epidemiology (STROBE) statement.^[^ ^53^ ^]^ The procedures used for saliva sampling and annotation were performed following the guidelines and experimental protocols approved by the ethics committee.^[^ ^54^ ^]^ The inclusion criteria considered participants who had an active lesion (pre‐surgical) in the oral cavity at the time of saliva collection. Additionally, sex distribution was not considered a selection factor, as this type of cancer is more prevalent in men. Patients who had received prior treatment at other centers were excluded. Detailed patient information is provided in Table S2 (Supporting Information). Therefore, saliva samples were voluntarily obtained from OSCC patients without lymph node metastasis (designated as N0, *n* = 30) and patients with lymph node metastasis (designated as N+, *n* = 30). The saliva collection was performed in individuals who had not eaten for at least 1 h. First, they rinsed their mouths with 5 mL of drinking water, and saliva was subsequently harvested without stimulation into a glass receptacle. The saliva samples were aliquoted into 15 mL tubes and frozen at −80 °C for long‐term storage until use. Before use, the saliva samples were centrifuged at 1500 × *g* for 5 min at 4 °C to pellet the debris.^[^ ^54^ ^]^

### Coomassie Blue Staining and Western Blot Analysis

Commercial recombinant proteins LTA4H and COL6A1 (10 µg each), along with the cloned protein CSTB (10 µg), were subjected to 10–15% SDS‐PAGE gel electrophoresis and stained with Coomassie Blue to detect protein bands (Figure S4a, Supporting Information). Saliva samples (20 µg of total protein) underwent the same procedure (Figure S4b, Supporting Information). The Western blot analysis was performed as previously reported.^[^ ^19^ ^]^ The primary antibodies used were anti‐CSTB (1:500), anti‐LTA4H (1:500), and anti‐COL6A1 (1:500). Western blots were conducted using 10 µg of recombinant proteins and 20 µg of total protein from saliva. Following electrophoresis, proteins were transferred onto nitrocellulose membranes, blocked with milk powder, and incubated with secondary antibodies. Detection was achieved using chemiluminescence with the ECL kit (Amersham Biosciences). Uncropped scans of all blots are shown in Figure S4c,d (Supporting Information).

### EIS Measurement

The EIS measurements were immediately conducted after the biological fluid deposition using the impedance analyzer Solartron model SI 1260 A (Solartron Analytical, Farnborough, Hampshire, UK), in the frequency range from 1 MHz to 25 mHz (10 points per decade, 100 mV oscillation amplitude, and 0 V offset voltage). All the measurements were carried out in the same conditions. To determine the calibration curve for each potential biomarker, curves were obtained in triplicates. The specificity and reproducibility of the IDEs were evaluated by conducting EIS measurements of the samples intra‐ and inter‐days, in triplicates. Moreover, to access the biosensor selectivity, known concentrations of recombinant protein were spiked on a saliva sample that was previously analyzed by EIS. In this way, it was possible to obtain the recovery rate for each biosensor and determine its selectivity.

### Supervised Classification Analysis

The 17 supervised classification models utilized in this analysis were sourced from four Python packages (v3.8.16): The four Python packages used were Scikit‐Learn (v1.2.2),^[^ ^55^ ^]^ CatBoost (v1.1.1),^[^ ^56^ ^]^ LightGBM (v3.3.5),^[^ ^57^ ^]^ and XGBoost (v1.7.4).^[^ ^58^ ^]^ The methodologies delineated subsequently are similarly deployed in four experimental scenarios: CSTB, LTA4H, COL6A1 and a combination thereof. Extreme outlier values in each column exceeding threefold the higher or lower fence had their lines removed from the dataset. The higher and lower fences in an IQR (interquartile range) are values calculated to identify outliers in a dataset. The higher fence is calculated as Q3 + 1.5IQR, and the lower fence is calculated as Q1 – 1.5IQR, where Q1 is the first quartile and Q3 is the third quartile. Then, the data were divided into training and test sets in a 4:1 ratio, respectively, to maintain the proportions of the two classes. To visualize and test the predictive potential of each of the predictive attributes, the distribution of the attributes and a less complex model such as logistic regression were fitted with the individualized data for each attribute (column).

This stage is crucial for substantiating the necessity of employing machine learning for this analysis and underscoring the significance of utilizing multiple predictor attributes. The data were normalized using the Scikit‐Learn method called RobustScaler (scaler fitted in the training set and transformed in both training and testing sets), which scales the attribute features using statistics that are robust to outliers. Due to the generation of negative values following the standardization process, the Scikit‐Learn method, MinMaxScaler, was employed as a secondary normalization option. This was because models such as multinomial Naive Bayes are unable to accept negative values, and the multilayer perceptron is known to perform better on data within the range of zero to one. Therefore, the multilayer perceptron was evaluated with data that had been normalized using both RobustScaler (MLP) and MinMaxScaler (MLP2). Subsequently, the learning models were evaluated with and without hyperparameter optimization. Hyperparameters are settings in a machine learning model that are defined before training and directly influence the model's capacity to learn and generalize the data. Figures S40–S43 (Supporting Information) show how features impact the machine learning model's prediction.

Consequently, a total of 34 supervised classification models were evaluated, with rankings based on the balanced accuracy value, weighted AUC‐ROC, and weighted F1‐score. This was due to the imbalance between the classes (N0 and N+). To ensure methodological consistency and eliminate optimization bias, a standardized hyperparameter tuning protocol was implemented across all experimental conditions. This optimization framework employed fivefold stratified cross‐validation within an 80‐20 train‐test data partition, with reproducibility ensured through controlled random state initialization. Hyperparameter search and model training were conducted exclusively on the training partition, with the test set reserved for final performance evaluation to provide unbiased generalization estimates. The consistency of the optimization strategy across all model configurations enables fair performance comparisons while accounting for algorithm‐specific hyperparameter sensitivity patterns. To ensure a fair comparison across all models, the same tuning procedure for all 17 models was used, employing 5‐fold stratified cross‐validation on the training set for hyperparameter optimization. The optimal model for each of the four experimental scenarios (merged, individualized CSTB, LTA4H and COL6A1) was exported for interpretability and explainability analyses and Figures S40– S43 (Supporting Information) show how features impact the machine learning model's prediction. LIME (local interpretable model‐agnostic explanations)^[^ ^59^ ^]^ and SHAP (Shapley additive explanations)^[^ ^60^ ^]^ are two of the most widely used techniques for interpreting complex machine learning models, particularly in high‐stakes fields like biosensing and clinical diagnostics. This strategy was employed to enhance the reliability of the predictions and the local and global performance of the selected models.

### Statistical Analysis

Prior to statistical analysis, the data were preprocessed to improve robustness and minimize bias. Extreme outliers were identified using the interquartile range (IQR) method, with a conservative threshold of three times the upper and lower fences, and subsequently removed. This cohort is composed of 60 patients collected by the collaborators from ICESP. The calculated sample size was based on achieving a confidence level of at least 85% with a maximum margin of error of 10%,^[^ ^61^ ^]^ considering a population size of 389485, corresponding to the number of oral cancer cases reported worldwide in 2022, as published by GLOBOCAN.^[^ ^2^ ^]^ Although the minimum required sample size was 52 to achieve this confidence, all 60 available samples were included to enhance statistical power. This also allowed for the exclusion of a few samples identified as outliers during data preprocessing. Samples were analyzed in triplicates and the results are presented as mean ± standard deviation (SD), unless stated otherwise.

## Conflict of Interest

The authors declare no conflict of interest.

## Author Contributions

A.F.P.L. initiated the concept and directed the project. A.F.P.L. and C.C.B.B. supervised the work. L.D.T.A. led the experiments and collected the overall data. D.C.G. contributed to antibody immobilization and saliva processing. L.G.S.A. contributed to the biosensor characterization and validation. A.G.S. and G.A.C. contributed to cloning, expression and purification of CSTB protein. D.H.S.C. and L.G.S.A. contributed to the interdigitated electrode fabrication. F.M.S.P. contributed to the supervised machine learning analysis. A.L.M., A.C.P.R., and T.B.B. contributed to patient selection and sample collection. All authors contributed the data analysis and provided feedback on the manuscript.

## Supporting information



Supporting Information

## Data Availability

The data that support the findings of this study are available from the corresponding author upon reasonable request

## References

[1] H. Sung , J. Ferlay , R. L. Siegel , M. Laversanne , I. Soerjomataram , A. Jemal , F. Bray , Ca‐Cancer J. Clin. 2021, 71, 209 . 33538338 10.3322/caac.21660

[2] F. Bray , M. Laversanne , H. Sung , J. Ferlay , R. L. Siegel , I. Soerjomataram , A. Jemal , Ca‐Cancer J. Clin. 2024, 74, 229. 38572751 10.3322/caac.21834

[3] R. L. Siegel , K. D. Miller , H. E. Fuchs , A. Jemal , Ca‐Cancer J. Clin. 2022, 72, 7. 35020204 10.3322/caac.21708

[4] A. C. Chi , T. A. Day , B. W. Neville , Ca‐Cancer J. Clin. 2015, 65, 401. 26215712 10.3322/caac.21293

[5] M. Gormley , T. Dudding , E. Sanderson , R. M. Martin , S. Thomas , J. Tyrrell , A. R. Ness , P. Brennan , M. Munafò , M. Pring , S. Boccia , A. F. Olshan , B. Diergaarde , R. J. Hung , G. Liu , G. D Smith , R. C. Richmond , Nat. Commun. 2020, 11, 6071. 33247085 10.1038/s41467-020-19822-6 PMC7695733

[6] K. D. Hunter , E. K. Parkinson , P. R. Harrison , Nat. Rev. Cancer 2005, 5, 127. 15685196 10.1038/nrc1549

[7] F. W. Mello , G. Melo , J. J. Pasetto , C. A. B. Silva , S. Warnakulasuriya , E. R. C. Rivero , Clin. Oral Invest. 2019, 23, 2849. 10.1007/s00784-019-02958-1 31111280

[8] Z. Jiang , C. Wu , S. Hu , N. Liao , Y. Huang , H. Ding , R. Li , Y. Li , Int. J. Oral Sci. 2021, 13, 13. 33795644 10.1038/s41368-021-00117-5 PMC8016921

[9] A. K. D'Cruz , R. Vaish , N. Kapre , M. Dandekar , S. Gupta , R. Hawaldar , J. P. Agarwal , G. Pantvaidya , D. Chaukar , A. Deshmukh , S. Kane , S. Arya , S. Ghosh‐Laskar , P. Chaturvedi , P. Pai , S. Nair , D. Nair , R. Badwe , N. Engl. J. Med. 2015, 373, 521. 26027881 10.1056/NEJMoa1506007

[10] A. Capote , V. Escorial , M. F. Muñoz‐Guerra , F. J. Rodríguez‐Campo , C. Gamallo , L. Naval , Head Neck 2007, 29, 3. 17103411 10.1002/hed.20482

[11] R. Zhou , X. Tang , Y. Wang , Nat. Rev. Cancer 2024, 24, 850. 39433978 10.1038/s41568-024-00754-y

[12] V. Garzarelli , F. Ferrara , E. Primiceri , M. S. Chiriacò , MethodsX 2022, 9, 101759. 35774416 10.1016/j.mex.2022.101759 PMC9237943

[13] J. D. O. Sá , L. D. Trino , A. K. Oliveira , A. F. B. Lopes , D. C. Granato , A. G. C. Normando , E. S. Santos , L. X. Neves , C. M. Carnielli , A. F. Paes Leme , Expert Rev. Proteomics 2021, 18, 261. 33945368 10.1080/14789450.2021.1924685

[14] R. M. Nagler , Oral Oncol. 2009, 45, 1006. 19828359 10.1016/j.oraloncology.2009.07.005

[15] D. C. Granato , C. M. Carnielli , L. D. Trino , A. F. Busso‐Lopes , G. A. Câmara , A. G. C. Normando , H. V. R. Filho , R. R. Domingues , S. Yokoo , B. A. Pauletti , F. M. Patroni , A. R. Santos‐Silva , M. A. Lopes , T. B. Brandão , A. C. Prado‐Ribeiro , P. S. Lopes‐de Oliveira , G. P. Telles , A. F. Paes Leme , J. Proteome Res. 2024, 23, 2148. 38785273 10.1021/acs.jproteome.4c00093 PMC11166140

[16] A. F. Busso‐Lopes , L. X. Neves , G. A. Câmara , D. C. Granato , M. A. M. Pretti , H. Heberle , F. M. S. Patroni , J. Sá , S. Yokoo , C. Rivera , R. R. Domingues , A. G. C. Normando , T. De Rossi , B. P. Mello , N. A. L. Galdino , B. A. Pauletti , P. A. Lacerda , A. A. N. Rodrigues , A. L. M. Casarim , R. A. de Lima‐Souza , I. I. Damas , F. V. Mariano , K. J. Gollob , T. S. Medina , N. K. Cervigne , A. C. Prado‐Ribeiro , T. B. Brandão , L. L. Villa , M. Uno , M. Boroni , et al., Nat. Commun. 2022, 13, 6725. 36344512 10.1038/s41467-022-34407-1 PMC9640649

[17] L.‐M. Chi , Y.‐C. Hsiao , K.‐Y. Chien , S.‐F. Chen , Y.‐N. Chuang , S.‐Y. Lin , W.‐S. Wang , I. Y.‐F. Chang , C. Yang , L. J. Chu , W.‐F. Chiang , C.‐Y. Chien , Y.‐S. Chang , K.‐P. Chang , J.‐S. Yu , J. Proteomics 2020, 211, 103571. 31689561 10.1016/j.jprot.2019.103571

[18] W. Yan , R. Apweiler , B. M. Balgley , P. Boontheung , J. L. Bundy , B. J. Cargile , S. Cole , X. Fang , M. Gonzalez‐Begne , T. J. Griffin , F. Hagen , S. Hu , L. E. Wolinsky , C. S. Lee , D. Malamud , J. E. Melvin , R. Menon , M. Mueller , R. Qiao , N. L. Rhodus , J. R. Sevinsky , D. States , J. L. Stephenson , S. Than , J. R. Yates , W. Yu , H. Xie , Y. Xie , G. S. Omenn , J. A. Loo , et al., Proteomics: Clin. Appl. 2009, 3, 116. 19898684 10.1002/prca.200800140 PMC2773554

[19] C. M. Carnielli , C. C. S. Macedo , T. De Rossi , D. C. Granato , C. Rivera , R. R. Domingues , B. A. Pauletti , S. Yokoo , H. Heberle , A. F. Busso‐Lopes , N. K. Cervigne , I. Sawazaki‐Calone , G. V. Meirelles , F. A. Marchi , G. P. Telles , R. Minghim , A. C. P. Ribeiro , T. B. Brandão , G. de Castro , W. A. González‐Arriagada , A. Gomes , F. Penteado , A. R. Santos‐Silva , M. A. Lopes , P. C. Rodrigues , E. Sundquist , T. Salo , S. D. da Silva , M. A. Alaoui‐Jamali , E. Graner , et al., Nat. Commun. 2018, 9, 3598. 30185791 10.1038/s41467-018-05696-2 PMC6125363

[20] R. Goldoni , A. Scolaro , E. Boccalari , C. Dolci , A. Scarano , F. Inchingolo , P. Ravazzani , P. Muti , G. Tartaglia , Biosensors 2021, 11, 396. 34677352 10.3390/bios11100396 PMC8533918

[21] R. M. L. da Silva , L. G. S. Albano , T. P. Vello , W. W. R. de Araújo , D. H. S. de Camargo , L. D. Palermo , C. C. Corrêa , C. Wöll , C. C. B. Bufon , Adv. Electron. Mater. 2022, 8, 2200175.

[22] L. G. S. Albano , D. H. S. de Camargo , G. R. Schleder , S. G. Deeke , T. P. Vello , L. D. Palermo , C. C. Corrêa , A. Fazzio , C. Wöll , C. C. B. Bufon , Small 2021, 17, 2101475. 10.1002/smll.202101475 34288416

[23] L. G. S. Albano , T. P. Vello , D. H. S. de Camargo , R. M. L. da Silva , A. C. M. Padilha , A. Fazzio , C. C. B. Bufon , Nano Lett. 2020, 20, 1080. 31917590 10.1021/acs.nanolett.9b04355

[24] T. P. Vello , L. G. S. Albano , T. C. dos Santos , J. C. Colletti , C. V. Santos Batista , V. F. C. Leme , T. C. dos Santos , M. P. D. C. Miguel , D. H. S. de Camargo , C. C. Bof Bufon , Small 2024, 20, 2305501. 10.1002/smll.202305501 37752688

[25] A. Radwan , H. Jin , D. He , S. Mu , Nano‐Micro Lett. 2021, 13, 132. 10.1007/s40820-021-00656-w PMC8169752 34138365

[26] Y. Peng , J. Xu , J. Xu , J. Ma , Y. Bai , S. Cao , S. Zhang , H. Pang , Adv. Colloid Interface Sci. 2022, 307, 102732. 35870249 10.1016/j.cis.2022.102732

[27] Y. Li , Y. Xu , W. Yang , W. Shen , H. Xue , H. Pang , Small 2018, 14, 1704435. 10.1002/smll.201704435 29750438

[28] L. D. Trino , L. G. S. Albano , D. C. Granato , A. G. Santana , D. H. S. de Camargo , C. C. Correa , C. C. Bof Bufon , A. F. Paes Leme , Chem. Mater. 2021, 33, 1293.

[29] H. Yang , J. Wang , C. Yang , X. Zhao , S. Xie , Z. Ge , J. Electrochem. Soc. 2018, 165, H247.

[30] Q. Zhang , L. Zhang , H. Dai , Z. Li , Y. Fu , Y. Li , J. Electroanal. Chem. 2018, 823, 40.

[31] Z. Liu , Y. Liu , in Metal−Organic Frameworks for Environmental Sensing, American Chemical Society, Washington 2021, pp. 1–31.

[32] K. S. Park , Z. Ni , A. P. Côté , J. Y. Choi , R. Huang , F. J. Uribe‐Romo , H. K. Chae , M. O'Keeffe , O. M. Yaghi , Proc. Natl. Acad. Sci. USA 2006, 103, 10186. 16798880 10.1073/pnas.0602439103 PMC1502432

[33] A. Paul , I. K. Banga , S. Muthukumar , S. Prasad , ACS Omega 2022, 7, 26993. 35967010 10.1021/acsomega.2c00737 PMC9366767

[34] A. Dizon , M. E. Orazem , Electrochim. Acta 2019, 327, 135000.

[35] J. A. Allegretto , D. Onna , S. A. Bilmes , O. Azzaroni , M. Rafti , Chem. Mater. 2024, 36, 5814. 38883435 10.1021/acs.chemmater.4c01069 PMC11171283

[36] F. Tian , A. M. Cerro , A. M. Mosier , H. K. Wayment‐Steele , R. S. Shine , A. Park , E. R. Webster , L. E. Johnson , M. S. Johal , L. Benz , J. Phys. Chem. C 2014, 118, 14449.

[37] K. Radhakrishnan , P. Panneerselvam , M. Marieeswaran , Anal. Methods 2019, 11, 490.

[38] B. Alberts , A. Johnson , J. Lewis , M. Raff , K. Roberts , P. Walter , Molecular Biology of the Cell, 4th ed., Garland Science, New York 2002,

[39] Y. Miao , D. T. Lee , M. D. de Mello , M. K. Abdel‐Rahman , P. Corkery , J. A. Boscoboinik , D. H. Fairbrother , M. Tsapatsis , Chem. Commun. 2021, 57, 5250. 10.1039/d1cc00252j 33904549

[40] J. F. Moulder , Handbook of X‐Ray Photoelectron Spectroscopy: A Reference Book of Standard Spectra for Identification and Interpretation of XPS Data, Physical Electronics Division, Perkin‐Elmer Corporation, Waltham, MA 1992.

[41] C. Qiu , F. Cai , Y. Wang , Y. Liu , Q. Wang , C. Zhao , J. Colloid Interface Sci. 2020, 565, 351. 31981844 10.1016/j.jcis.2019.12.070

[42] M. Chin , C. Cisneros , S. M. Araiza , K. M. Vargas , K. M. Ishihara , F. Tian , RSC Adv. 2018, 8, 26987. 30174827 10.1039/c8ra03459a PMC6088371

[43] M. Yan , J. Ye , Q. Zhu , L. Zhu , J. Huang , X. Yang , Anal. Chem. 2019, 91, 10156. 31283192 10.1021/acs.analchem.9b02169

[44] M. Ashoorirad , A. Fallah , M. Saviz , J. Mol. Liq. 2020, 320, 114488.

[45] M. Ashoorirad , M. Saviz , A. Fallah , J. Mol. Liq. 2020, 300, 112344.

[46] M. Itagaki , S. Suzuki , I. Shitanda , K. Watanabe , Electrochemistry 2007, 75, 649.

[47] M. Bolognesi , M. Prosa , M. Toerker , L. L Sanchez , M. Wieczorek , C. Giacomelli , E. Benvenuti , P. Pellacani , A. Elferink , A. Morschhauser , L. Sola , F. Damin , M. Chiari , M. Whatton , E. Haenni , D. Kallweit , F. Marabelli , J. Peters , S. Toffanin , Adv. Mater. 2023, 35, 2208719. 10.1002/adma.202208719 36932736

[48] T. M. B. F. Oliveira , M. Fátima Barroso , S. Morais , P. de Lima‐Neto , A. N. Correia , M. B. P. P. Oliveira , C. Delerue‐Matos , Talanta 2013, 106, 137. 23598106 10.1016/j.talanta.2012.12.017

[49] K. Zhang , J. Wang , T. Liu , Y. Luo , X. J. Loh , X. Chen , Adv. Healthcare Mater. 2021, 10, 2100734. 10.1002/adhm.202100734 34165240

[50] G. F. Giordano , L. F. Ferreira , Í. R. S. Bezerra , J. A. Barbosa , J. N. Y. Costa , G. J. C. Pimentel , R. S. Lima , Anal. Bioanal. Chem. 2023, 415, 3683. 36637495 10.1007/s00216-023-04514-z PMC9838410

[51] J. N. Mandrekar , J. Thorac. Oncol. 2010, 5, 1315. 20736804 10.1097/JTO.0b013e3181ec173d

[52] C. L. Calvete , M. M. Caseiro , C. B. de Souza , UNILUS Ensino e Pesquisa 2015, 12, 41.

[53] E. von Elm , D. G. Altman , M. Egger , S. J. Pocock , P. C. Gøtzsche , J. P. Vandenbroucke , BMJ 2007, 335, 806. 17947786 10.1136/bmj.39335.541782.AD PMC2034723

[54] F. V. Winck , A. C. Prado Ribeiro , R. R Domingues , L. Y. Ling , D. M. Riaño‐Pachón , C. Rivera , T. B. Brandão , A. F. Gouvea , A. R. Santos‐Silva , R. D. Coletta , A. F. Paes Leme , Sci. Rep. 2015, 5, 16305. 26538482 10.1038/srep16305 PMC4633731

[55] F. Pedregosa , G. Varoquaux , A. Gramfort , V. Michel , B. Thirion , O. Grisel , M. Blondel , P. Prettenhofer , R. Weiss , V. Dubourg , J. Vanderplas , A. Passos , D. Cournapeau , M. Brucher , M. Perrot , É. Duchesnay , J. Mach. Learn. Res. 2011, 12, 2825.

[56] L. Prokhorenkova , G. Gusev , A. Vorobev , A. V. Dorogush , A. Gulin , in Proc. of the 32nd Int. Conf. on Neural Information Processing Systems, (Eds.: S. Bengio , H. M. Wallach , H. Larochelle , K. Grauman , N. Cesa‐Bianchi ), Curran Associates Inc., Red Hook, NY, USA, 2018, pp. 6639–6649.

[57] G. Ke , Q. Meng , T. Finley , T. Wang , W. Chen , W. Ma , Q. Ye , T.‐Y. Liu , in Proceedings of the 31st International Conference on Neural Information Processing Systems, (Eds.: U. V. Luxburg , I. Guyon , S. Bengio , H. Wallach , R. Fergus ), Curran Associates, Inc., Red Hook, NY, USA 2017, pp. 3149–3157.

[58] T. Chen , C. Guestrin , in Proc. of the 22nd ACM SIGKDD Int. Conf. on Knowledge Discovery and Data Mining, Association For Computing Machinery, (Eds.: B. Krishnapuram , M. Shah , A. Smola , C. Aggarwal , D. Shen , R. Rastogi ), New York, NY, USA, 2016, pp. 785‐794.

[59] M. T. Ribeiro , S. Singh , C. Guestrin , in KDD '16: Proc. of the 22nd ACM SIGKDD Int. Conf. on Knowledge Discovery and Data Mining, (Eds.: B. Krishnapuram , M. Shah , A. Smola , C. Aggarwal , D. Shen , R. Rastogi ), Association For Computing Machinery, New York, NY 2016, pp. 1135–1144 10.48550/arXiv.1602.04938.

[60] S. M. Lundberg , S.‐I. Lee , in Proceedings of the 31st International Conference on Neural Information Processing Systems, (Eds.: U. V. Luxburg , I. Guyon , S. Bengio , H. Wallach , R. Fergus ), Curran Associates Inc., Red Hook, NY, USA 2017, pp. 4768–4777, 10.48550/arXiv.1705.07874.

[61] C. Metcalfe , Stat. Med. 2001, 20, 324.

