# Supplementary material for: Noninvasive and Sensitive Biosensor for the Detection of Oral Cancer Prognostic Biomarkers
Source: Small. 2025 Jul 29;21(47):e04278. doi: 10.1002/smll.202504278 (PMC12658947; doi:10.1002/smll.202504278)
Supplement: Supplementary file 1 — Supporting Information [file SMLL-21-e04278-s001.pdf]

NANO · MICRO
small

Supporting Information

for *Small*, DOI 10.1002/smll.202504278

Noninvasive and Sensitive Biosensor for the Detection of Oral Cancer Prognostic Biomarkers

Luciana D. Trino Albano, Daniela C. Granato, Luiz G. S. Albano, Fábio M. S. Patroni, Aline G. Santana, Guilherme A. Câmara, Davi H. S. de Camargo, Ana L. Mores, Thaís B. Brandão, Ana C. Prado-Ribeiro, Carlos C. B. Bufon* and Adriana F. Paes Leme**

Noninvasive and Sensitive Biosensor for the Detection of Oral Cancer Prognostic Biomarkers

Luciana D. Trino Albano, Daniela C. Granato, Luiz G. S. Albano, Fabio M. S. Patroni, Aline G. Santana, Guilherme A. Câmara, Davi H. S. de Camargo, Ana L. Mores, Thaís B. Brandão, Ana C. Prado-Ribeiro, Carlos C. B. Bufon*, Adriana F. Paes Leme**

Luciana D. Trino Albano
Center for Information Technology Renato Archer (CTI Renato Archer), 13069-901, Campinas, São Paulo, Brazil.
E-mail: luciana.albano@cti.gov.br
Luciana D. Trino Albano, Daniela C. Granato, Luiz G. S. Albano, Fabio M. S. Patroni, Aline G. Santana, Guilherme A. Câmara, Adriana F. Paes Leme
Brazilian Biosciences National Laboratory (LNBio), Brazilian Center for Research in Energy and Materials (CNPEM), 13083-970, Campinas, São Paulo, Brazil.
E-mail: adriana.paesleme@lnbio.cnpem.br
Luiz G. S. Albano, Davi H. S. de Camargo
Brazilian Nanotechnology National Laboratory (LNNano), Brazilian Center for Research in Energy and Materials (CNPEM), 13083-970, Campinas, São Paulo, Brazil.
Ana C. Prado-Ribeiro, Thais B. Brandão
Dental Oncology Service, Instituto do Câncer do Estado de São Paulo, Faculdade de Medicina da Universidade de São Paulo (ICESP-FMUSP), São Paulo, 01246-000, Brazil.
Ana L. Mores, Ana C. Prado-Ribeiro, Thais B. Brandão
Oral Diagnosis Department, Semiology and Oral Pathology Areas, Piracicaba Dental School, State University of Campinas (UNICAMP), Piracicaba, São Paulo, 13414-903, Brazil.
Carlos C. B. Bufon
Physics Department, Institute of Geosciences and Exact Sciences, São Paulo State University (Unesp), 13506-900, Rio Claro, São Paulo, Brazil.
E-mail: cesar.bof@unesp.br

Table of Content

Figure S1. Schematic illustration of the IDE photolithography fabrication process. 4

Figure S2. IDE characterization. 5

Table S1. Standard curve of the biomarkers. 6

Figure S3. Stability of the ZIF-8 nanoparticles after incubation with PBS. 7

Figure S4. Validation of the immunoreaction between the antibodies and recombinant proteins used in the biosensor. 8

Figure S5. EIS analysis. 9

Figure S6. Linear detection region for the biomarkers by capacitance, control test, and biomarkers concentration in saliva samples. 10

Figure S7. EIS impedance values for 30 N0 and 30 N+ saliva samples. 11

Figure S8. Machine learning (ML) approaches were applied to identify the most effective classifiers for the LTA4H biomarker. 12

Figure S9. Machine learning (ML) approaches were applied to identify the most effective classifiers for the COL6A1 biomarker. 13

Figure S10. Machine learning (ML) approaches were applied to identify the most effective classifiers for the CSTB biomarker. 14

Figure S11. Machine learning (ML) approaches were applied to identify the most effective classifiers, combining LTA4H, CSTB, and COL6A1 biomarkers. 15

Figure S12. Classification of ML supervised models, with (first panel) and without (second panel) hyperparameter optimization, for LTA4H. 16

Figure S13. Classification of ML supervised models, with (first panel) and without (second panel) hyperparameter optimization, for COL6A1. 17

Figure S14. Classification of ML supervised models, with (first panel) and without (second panel) hyperparameter optimization, for CSTB. 18

Figure S15. Classification of ML supervised models, with (first panel) and without (second panel) hyperparameter optimization, for the combined analysis. 19

Figure S16. No optimized ML approaches considering the individual effect of the LTA4H biomarker for the training group. 20

Figure S17. No optimized ML approaches considering the individual effect of the LTA4H biomarker for the test group. 21

Figure S18. Optimized ML approaches considering the individual effect of LTA4H biomarker for the training group. 22

Figure S19. Optimized ML approaches considering the individual effect of LTA4H biomarker for the test group. 23

Figure S20. AUC-ROC curves for the non-optimized ML approaches for LTA4H. 24

Figure S21. AUC-ROC curves for the optimized ML approaches for LTA4H. 25

Figure S22. No optimized ML approaches considering the individual effect of COL6A1 biomarker for the training group. 26

Figure S23. No optimized ML approaches considering the individual effect of the COL6A1 biomarker for the test group. 27

Figure S24. Optimized ML approaches considering the individual effect of COL6A1 biomarker for the training group. 28

Figure S25. Optimized ML approaches considering the individual effect of COL6A1 biomarker for the test group. 29

Figure S26. AUC-ROC curves for the non-optimized ML approaches for COL6A1. 30

Figure S27. AUC-ROC curves for the optimized ML approaches for COL6A1. 31

Figure S28. No optimized ML approaches considering the individual effect of CSTB biomarker for the training group. 32

Figure S29. No optimized ML approaches considering the individual effect of CSTB biomarker for the test group. 33

Figure S30. Optimized ML approaches considering the individual effect of CSTB biomarker for the training group. 34

Figure S31. Optimized ML approaches considering the individual effect of CSTB biomarker for the test group. 35

Figure S32. AUC-ROC curves for the non-optimized ML approaches for CSTB. 36

Figure S33. AUC-ROC curves for the optimized ML approaches for CSTB. 37

Figure S34. No optimized ML approaches considering the combined effect of the biomarkers for the training group. 38

Figure S35. No optimized ML approaches considering the combined effect of the biomarkers for the test group. 39

Figure S36. Optimized ML approaches considering the combined effect of the biomarkers for the training group. 40

Figure S37. Optimized ML approaches considering the combined effect of the biomarkers for the test group. 41

Figure S38. AUC-ROC curves for the non-optimized ML approaches for all the biomarkers. 42

Figure S39. AUC-ROC curves for the optimized ML approaches for all the biomarkers. 43

Table S2. Clinical and demographic data of OSCC patient samples included in the analysis. 44

Table S3. Quantitative performance metrics for the individual effect of LTA4H. 45

Table S4. Quantitative performance metrics for the individual effect of COL6A1. 46

Table S5. Quantitative performance metrics for the individual effect of CSTB. 47

Table S6. Quantitative performance metrics for the combined effect of LTA4H, COL6A1, and CSTB. 48

Figure S40. Interpretability of ML models considering the individual effect of LTA4H. 49

Figure S41. Interpretability of ML models considering the individual effect of COL6A1. 50

Figure S42. Interpretability of ML models considering the individual effect of CSTB. 51

Figure S43. Interpretability of ML models considering the combined effect of LTA4H, COL6A1, and CSTB biomarkers. 52

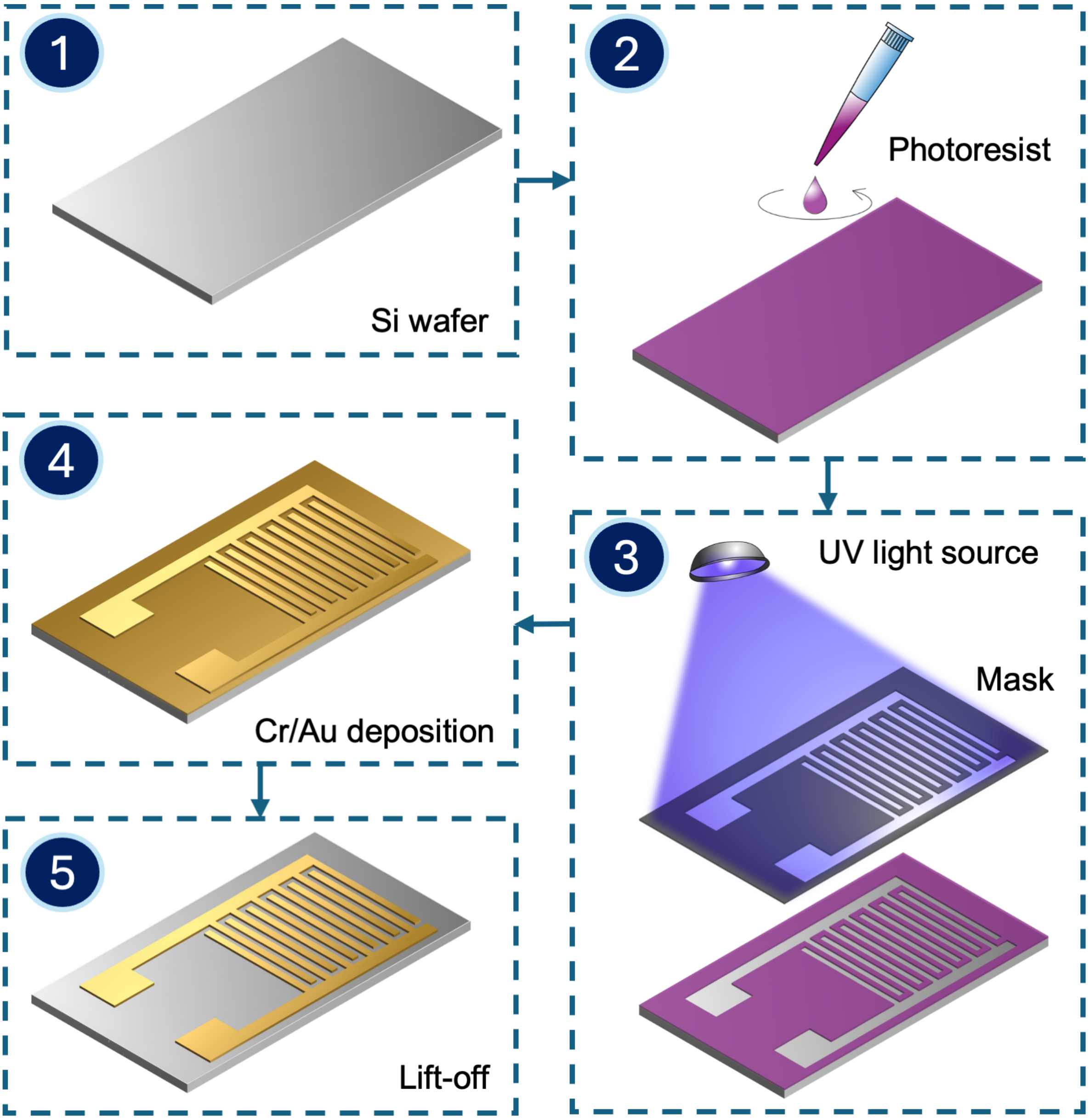

Figure S1. Schematic illustration of the IDE photolithography fabrication process. First, thin silicon wafers are used as substrates and dry-cleaned with plasma to ensure a clean and highly adhesive surface, ready to receive the photoresist. The second step consists of the deposition of a uniform AZ 5214E photoresist layer using the spin-coating. The substrate rotates rapidly, spreading the photoresist solution, followed by solvent evaporation to form a uniform layer. In the third step, the photoresist is exposed to UV light through a patterned mask, enhancing its solubility during the subsequent development step. The next step consists of the deposition of Cr/Au (20/20 nm) layers using e-beam evaporation at a rate of 1 Å/s. The chromium layer enhances adhesion for the subsequent gold layer. In the final step, known as the lift-off process, the resist is removed using acetone, leaving the metal deposited only in the patterned areas directly on the substrate.

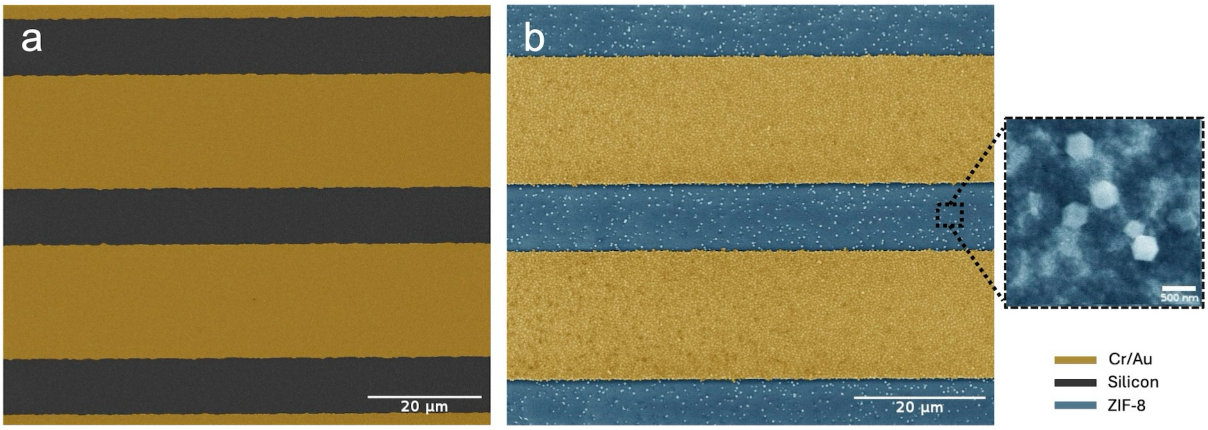

Figure S2. IDE characterization. a, SEM images of the IDE before and (**b**) after ZIF-8 growth. The thin film is achieved after 6 hours of ZIF-8 growth, where ZIF-8 particles can be seen connecting the Au arrays. The inset reveals ZIF-8 particles of two distinct sizes: 159 (± 22) nm and 252 (± 34) nm. The larger particles are more prominent on the top surface due to the extended exposure time.

Table S1. Standard curve of the biomarkers. Capacitance (*C*) values were calculated for the different concentrations of the biomarkers CSTB, LTA4H, and COL6A1.

Biomarker	Concentration [ng/mL]	*C* [F]	Standard Deviation
CSTB	31.25	9.44E-11	7.08E-12
	62.50	1.37E-10	4.65E-11
	125.00	2.51E-10	1.92E-11
	500.00	7.80E-10	7.48E-11
	1000.00	1.41E-09	1.41E-10
	4000.00	6.28E-09	2.52E-10
LTA4H	31.25	1.06E-10	4.20E-11
	62.50	2.04E-10	2.00E-11
	125.00	7.35E-10	1.40E-10
	500.00	5.14E-09	1.40E-09
	1000.00	1.83E-08	3.60E-09
COL6A1	0.97	3.43E-07	8.66E-08
	3.90	3.60E-08	7.05E-09
	15.60	9.80E-09	1.54E-09
	62.50	5.10E-10	1.89E-10
	250.00	1.08E-10	5.74E-12

All experiments were performed in triplicate.

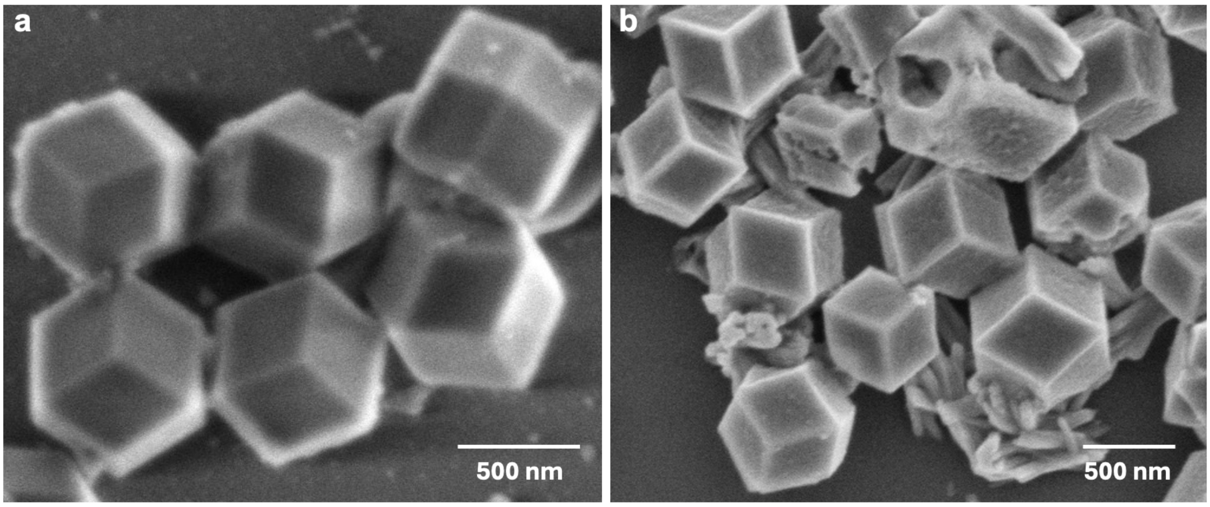

Figure S3. Stability of the ZIF-8 nanoparticles after incubation with PBS. a, ZIF-8 nanoparticles after 30 minutes and **b,** after 120 minutes of incubation in PBS. The short-term stability of ZIF-8 was accessed by incubating the biosensor in PBS in different time points and observing the ZIF-8 crystals by scanning electron microscopy (SEM). These intervals were selected to reflect the actual durations of antibody immobilization (120 minutes) and saliva exposure (30 minutes) in our experimental protocol. Although some degradation sub-product particles are observed, especially after 120 minutes of incubation, most of the ZIF-8 nanoparticles did not suffer substantially from etching, showing the same rhombic dodecahedron morphology. This analysis confirmed the material's integrity under conditions relevant to biosensor fabrication and use.

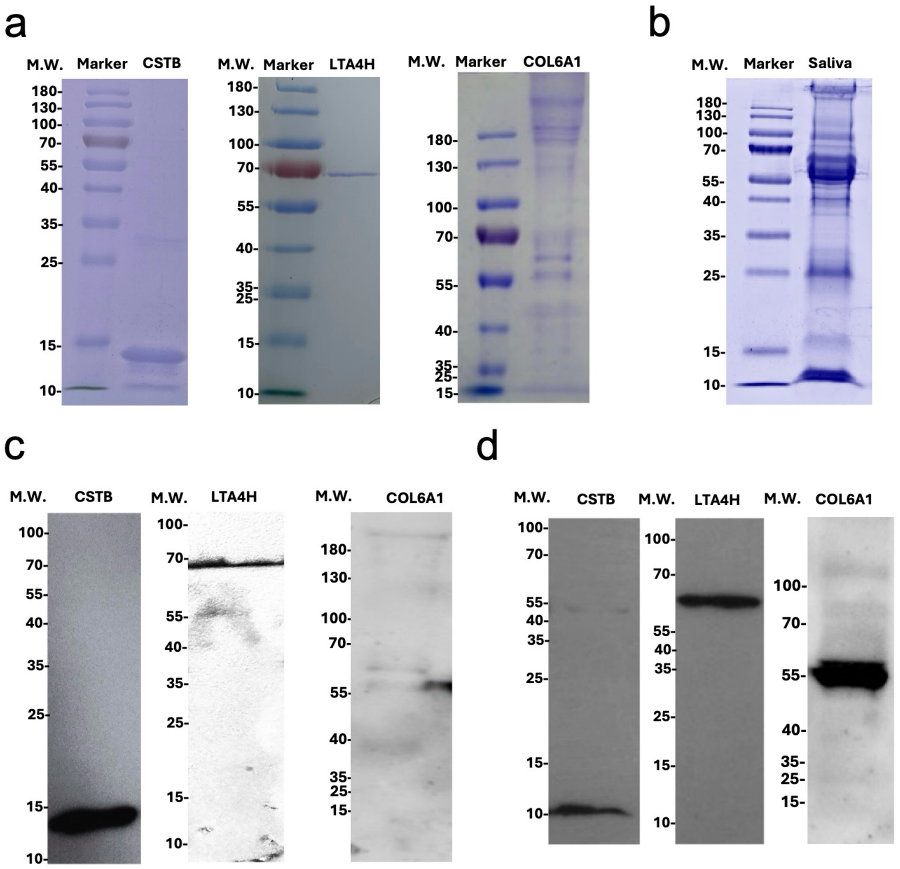

Figure S4. Validation of the immunoreaction between the antibodies and recombinant proteins used in the biosensor. a, SDS-PAGE polyacrylamide gel (10%) electrophoresis confirmed the presence of the recombinant proteins at 11.1 kDa (CSTB), 69.3 kDa (LTA4H), and 108.5 kDa (COL6A1). **b,** SDS-PAGE analysis of a saliva sample revealed various protein bands, including those corresponding to the molecular weights of the targeted biomarkers. **c,** Western blot analysis demonstrated the detection of the recombinant proteins CSTB, LTA4H, and COL6A1 by the antibodies used in the biosensor. **d,** Western blot analysis of the saliva sample confirmed that the antibodies for CSTB, LTA4H, and COL6A1 effectively recognize the respective biomarkers in saliva, validating their use in the biosensor. For clarification, according to the commercial supplier, in Western blot analysis the reduced and boiled recombinant COL6A1 protein (NBP1-97270) displays a prominent band around 55 kDa, and the same is observed for saliva.

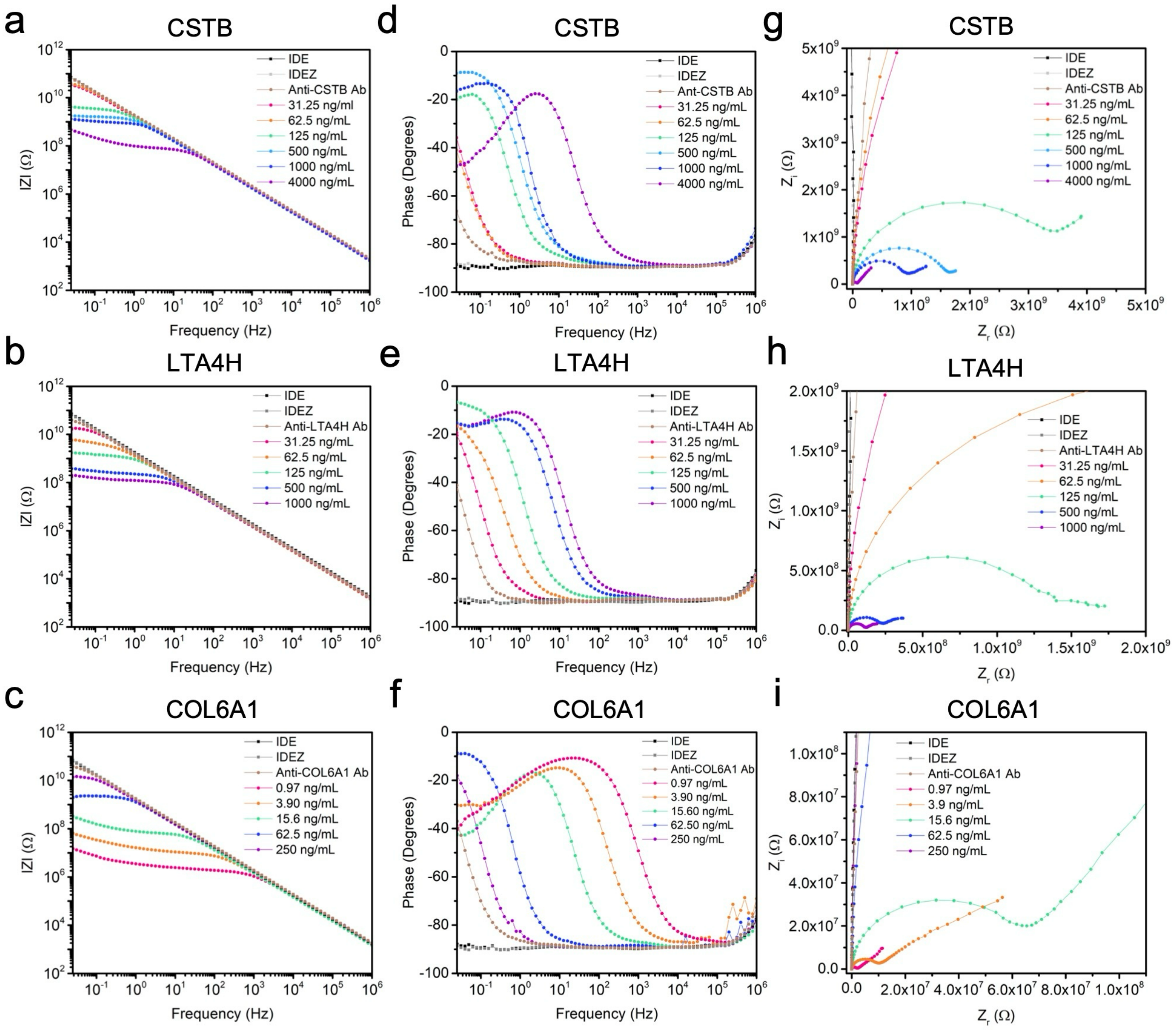

Figure S5. EIS analysis. a-c, Bode, (**d-f**) Phase angle, and (**g-i**) Nyquist plots for establishing the standard curves of CSTB, LTA4H, and COL6A1 biomarkers. Pristine IDE, the IDE after modification with ZIF-8 (IDEZ), and after the CSTB (Anti-CSTB Ab), LTA4H (Anti-LTA4H Ab), and COL6A1 (Anti-COL6A1 Ab) antibody deposition, showed a capacitive behavior. For CSTB and LTA4H, as the concentration of these proteins increases, the impedance decreases between 1 and 0.025 Hz. The Nyquist plot shows a reduction in the semicircle's diameter, indicating lower charge transfer resistance. In contrast, increasing COL6A1 concentration leads to an increase in impedance. The Nyquist plot shows a larger semicircle, indicating higher charge transfer resistance. This is attributed to COL6A1's ability to form a complex polymeric network as its concentration increases, leading to greater impedance. The experiments were performed in three technical replicates.

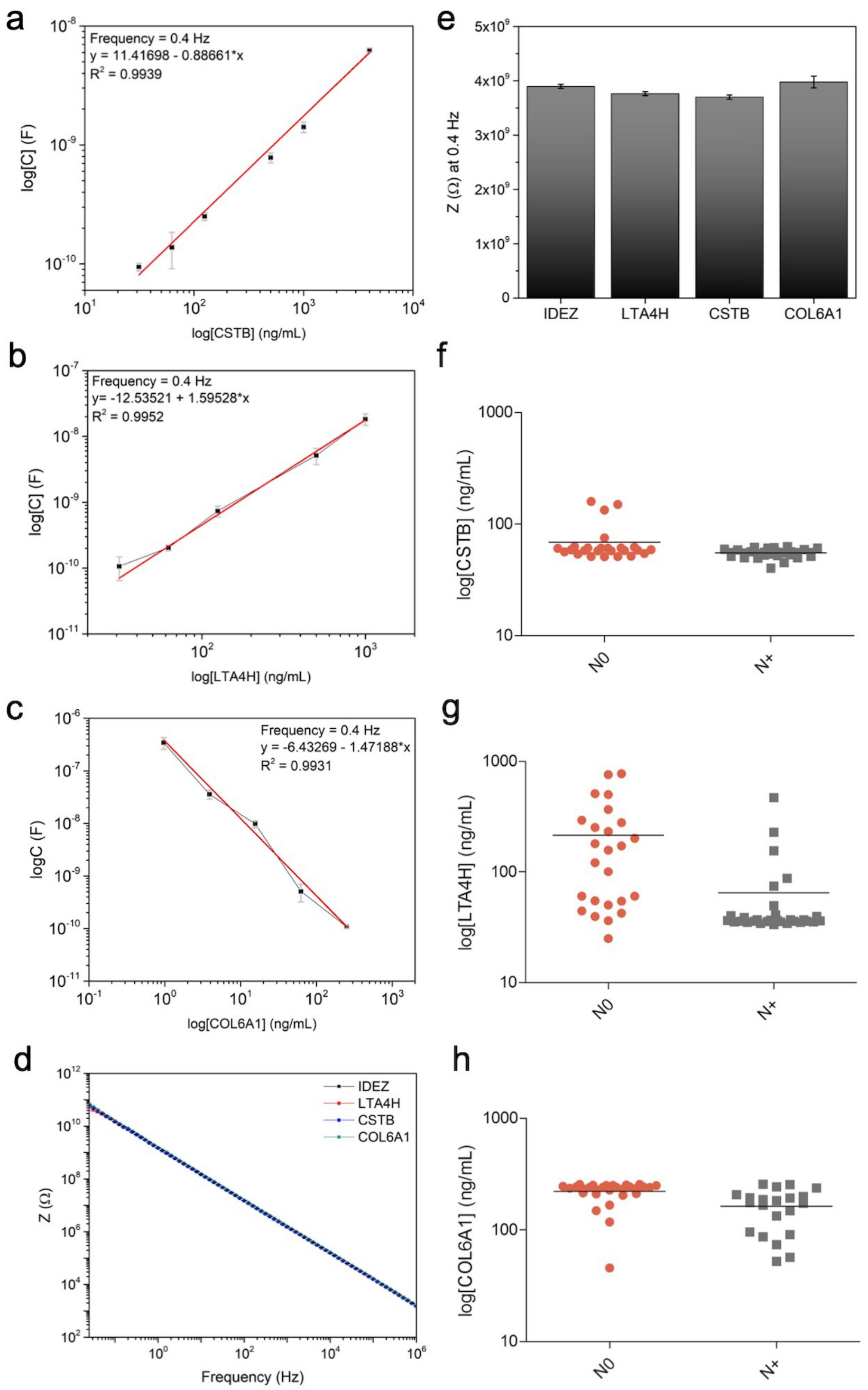

Figure S6. Linear detection region for the biomarkers by capacitance, control test, and biomarkers concentration in saliva samples. The linear curve for (**a**) CSTB and (**b**) LTA4H indicated a direct relationship with an increase in the biomarker concentration. **c,** For COL6A1 the opposite behavior was observed. **d,** The average Bode plot for the control test demonstrates that without antibody modification, the biosensor is unable to detect the biomarkers. **e,** Specifically, at the impedance value of 0.4 Hz, which falls within the biosensor's linear detection range, no response is observed in the absence of the antibody. In the control test, 100 ng/mL of each recombinant protein corresponding to the three biomarkers was used. The concentration of the biomarkers (**f**) CSTB, (**g**) LTA4H, and (**h**) COL6A1 in saliva samples from N0 and N+ OSCC patients, where a separation of prognostic groups can be observed. The linear curves and control experiments were performed in triplicate.

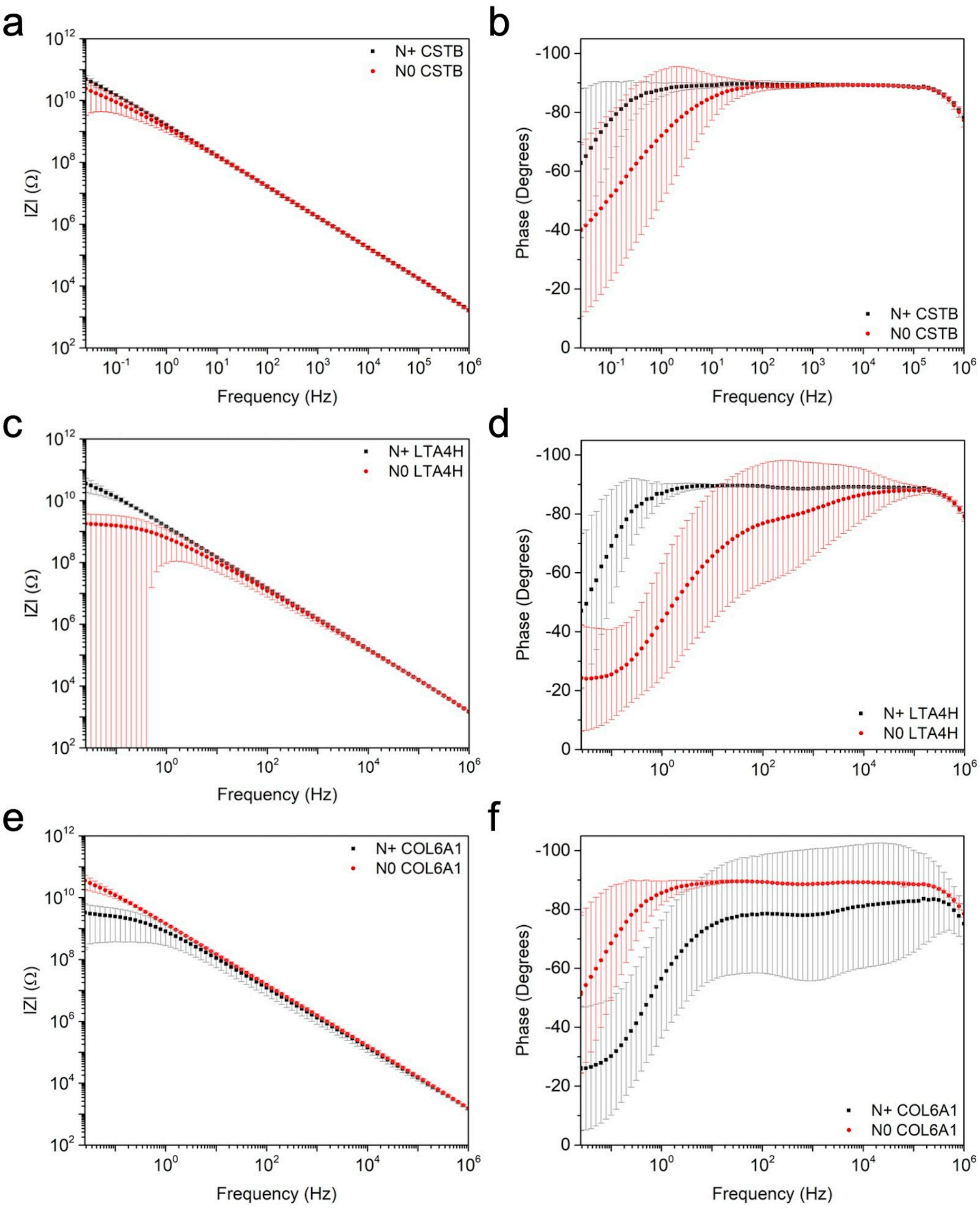

Figure S7. EIS impedance values for 30 N0 and 30 N+ saliva samples. For the CSTB biomarker, the (**a**) Bode and (**b**) phase angle plots reveal that differences between the samples are more pronounced in terms of capacitance. Similarly, for LTA4H, the (**c**) Bode and (**d**) phase angle plots also demonstrate that capacitance provides a clearer distinction between the groups. This same trend is observed in the (**e**) Bode and (**f**) phase angle plots for COL6A1.

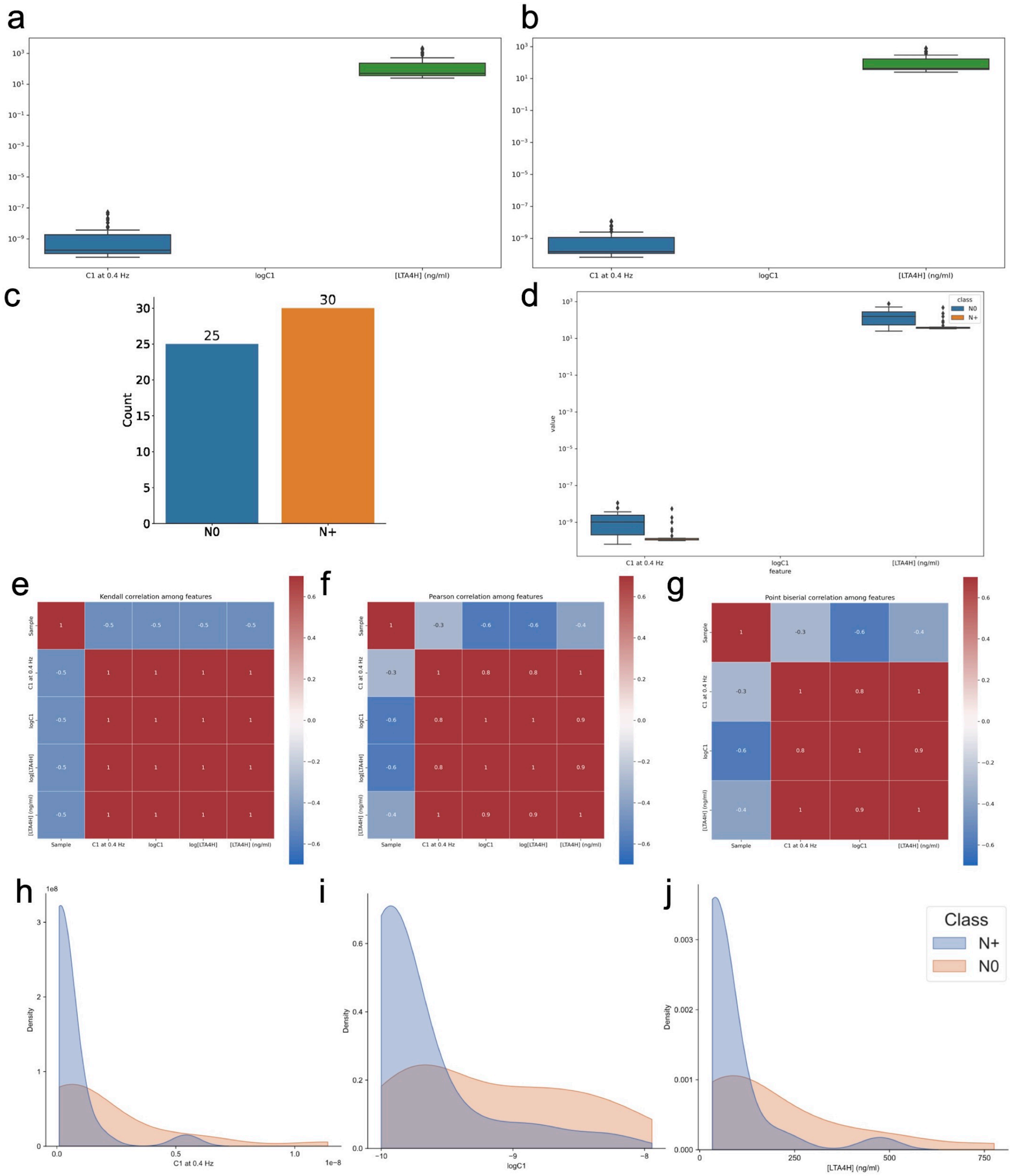

Figure S8. Machine learning (ML) approaches were applied to identify the most effective classifiers for the LTA4H biomarker. Initially, (**a**) outliers were detected and subsequently (**b**) removed to ensure more reliable results. **c,** Number of samples in each prognostic group after outlier removal. **d,** Samples concerning capacitance values, the logarithmic scale of capacitance, and LTA4H concentration (ng/mL). Several correlation analyses were performed, including (**e**) Kendall, (**f**) Pearson, and (**g**) Point-biserial, which revealed that no single variable could be used without compromising the overall correlation strength. From the (**h**) capacitance, the (**i**) logarithmic capacitance scale, and the (**j**) LTA4H concentration values, it is evident that result overlap makes it difficult to distinguish the prognostic groups using only one variable.

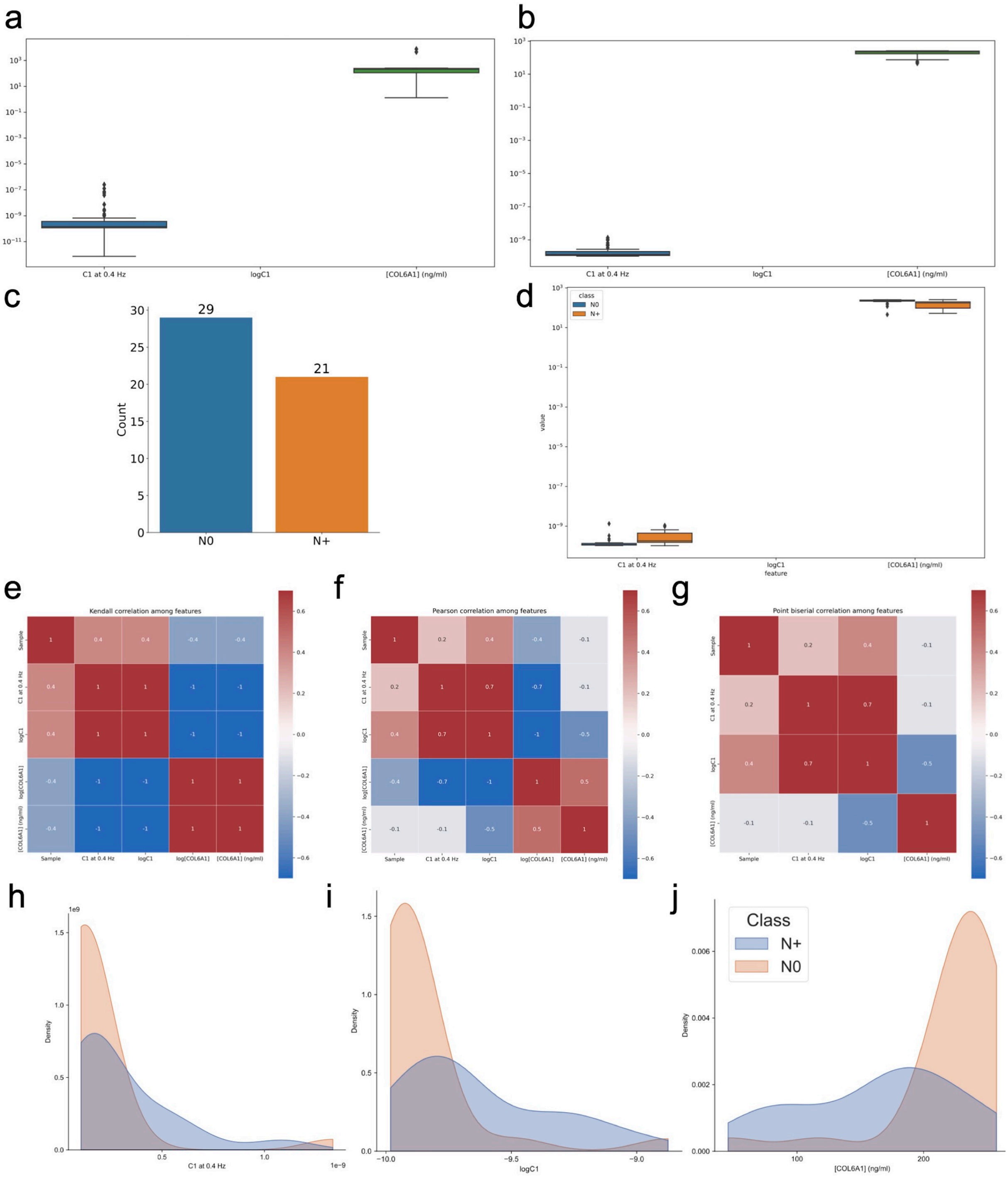

Figure S9. Machine learning (ML) approaches were applied to identify the most effective classifiers for the COL6A1 biomarker. Initially, (**a**) outliers were detected and subsequently (**b**) removed to ensure more reliable results. **c,** Number of samples in each prognostic group after outlier removal. **d,** Samples concerning capacitance values, the logarithmic scale of capacitance, and COL6A1 concentration (ng/mL). Several correlation analyses were performed, including (**e**) Kendall, (**f**) Pearson, and (**g**) Point-biserial, which revealed that no single variable could be used without compromising the overall correlation strength. From the (**h**) capacitance, the (**i**) logarithmic capacitance scale, and the (**j**) COL6A1 concentration values, it is evident that result overlap makes it difficult to distinguish the prognostic groups using only one variable.

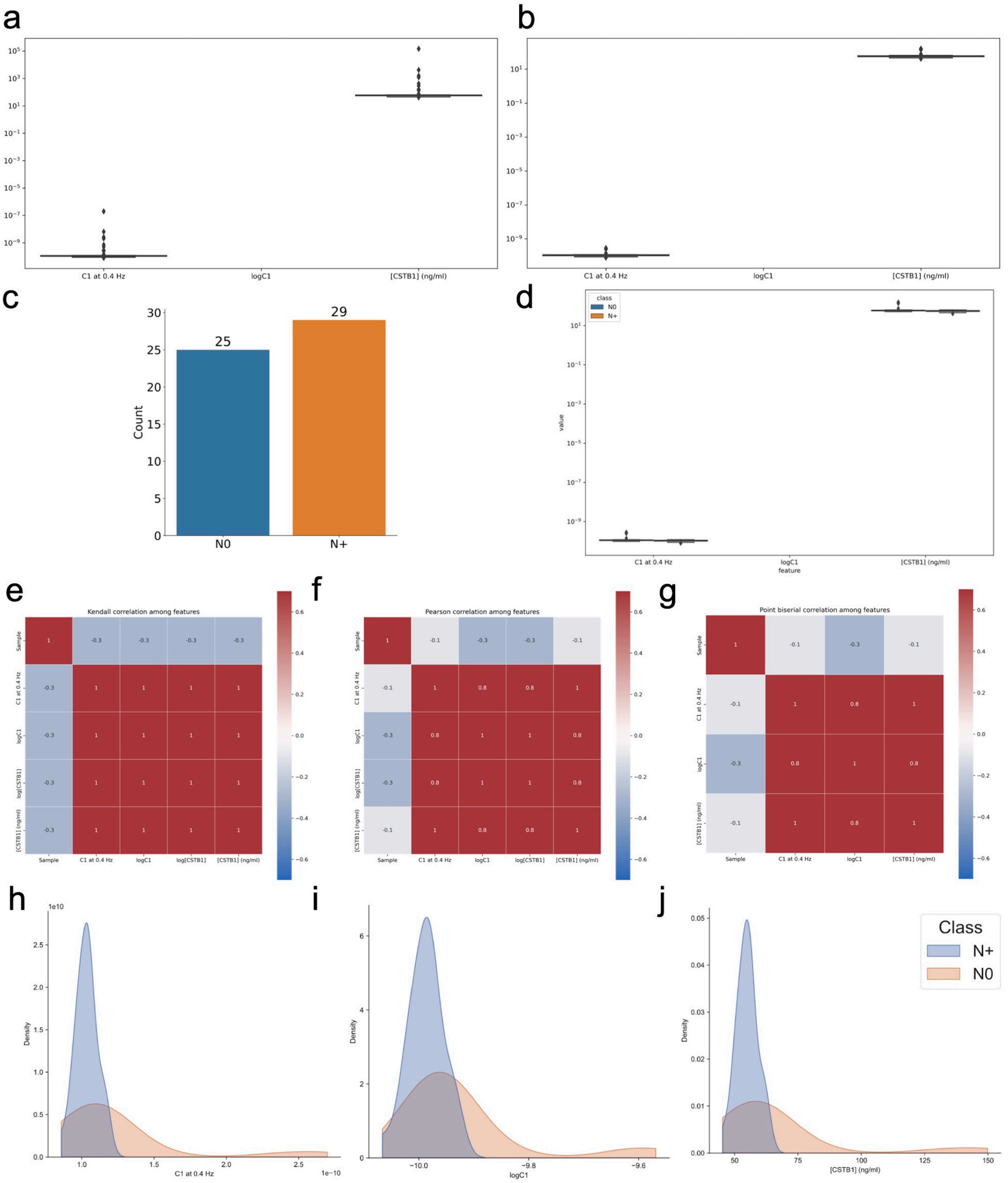

Figure S10. Machine learning (ML) approaches were applied to identify the most effective classifiers for the CSTB biomarker. Initially, (**a**) outliers were detected and subsequently (**b**) removed to ensure more reliable results. **c,** Number of samples in each prognostic group after outlier removal. **d,** Samples concerning capacitance values, the logarithmic scale of capacitance, and CSTB concentration (ng/mL). Several correlation analyses were performed, including (**e**) Kendall, (**f**) Pearson, and (**g**) Point-biserial, which revealed that no single variable could be used without compromising the overall correlation strength. From the (**h**) capacitance, the (**i**) logarithmic capacitance scale, and the (**j**) CSTB concentration values, it is evident that result overlap makes it difficult to distinguish the prognostic groups using only one variable.

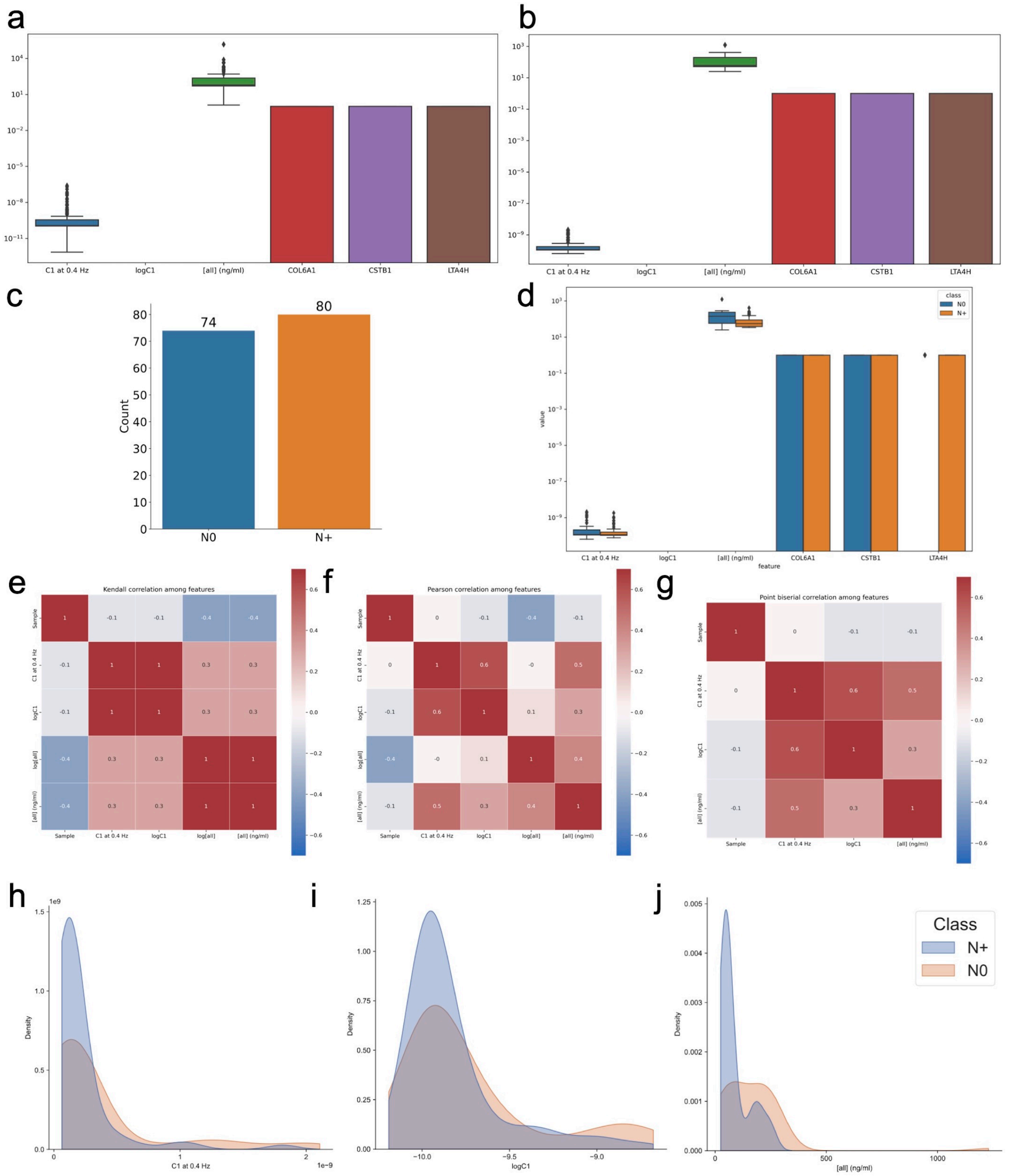

Figure S11. Machine learning (ML) approaches were applied to identify the most effective classifiers, combining LTA4H, CSTB, and COL6A1 biomarkers. Initially, (**a**) outliers were detected and subsequently (**b**) removed to ensure more reliable results. **c,** Number of samples in each prognostic group after outlier removal. **d,** Samples concerning capacitance values, the logarithmic scale of capacitance, and the biomarker`s concentration (ng/mL). Several correlation analyses were performed, including (**e**) Kendall, (**f**) Pearson, and (**g**) Point-biserial, which revealed that no single variable could be used without compromising the overall correlation strength. From the (**h**) capacitance, the (**i**) logarithmic capacitance scale, and the (**j**) biomarkers concentration values, it is evident that result overlap makes it difficult to distinguish the prognostic groups using only one variable.

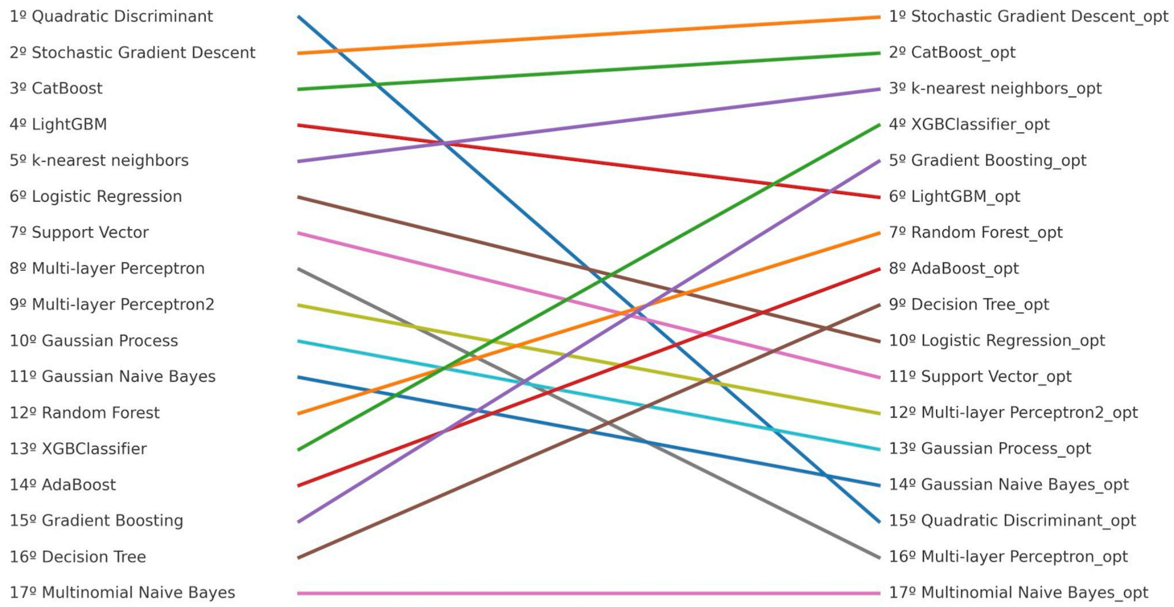

Figure S12. Classification of ML supervised models, with (first panel) and without (second panel) hyperparameter optimization, for LTA4H.

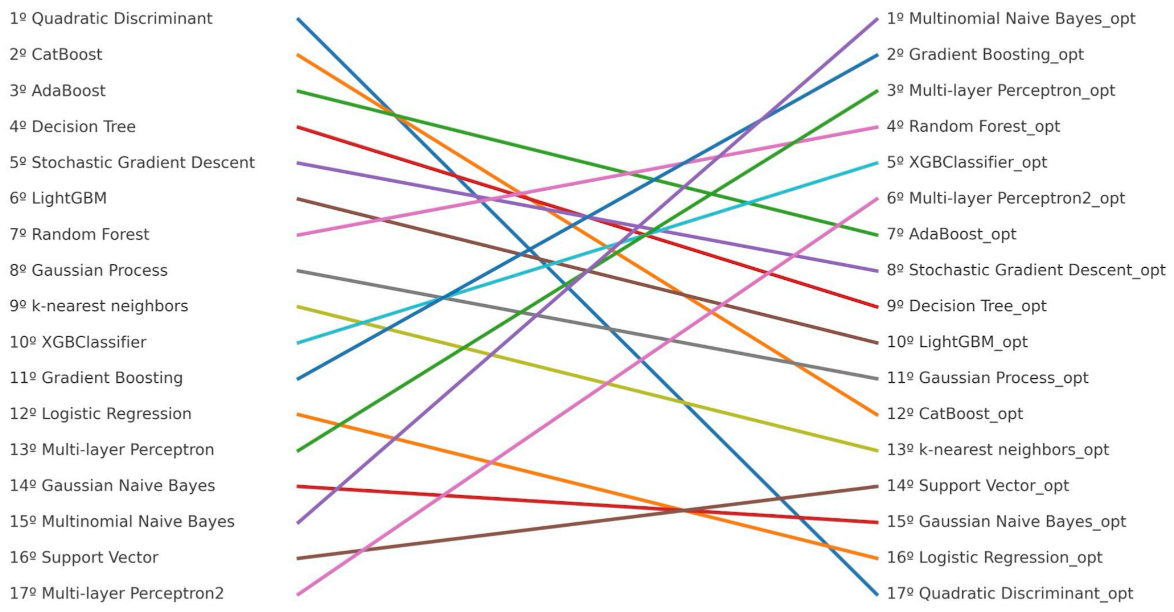

Figure S13. Classification of ML supervised models, with (first panel) and without (second panel) hyperparameter optimization, for COL6A1.

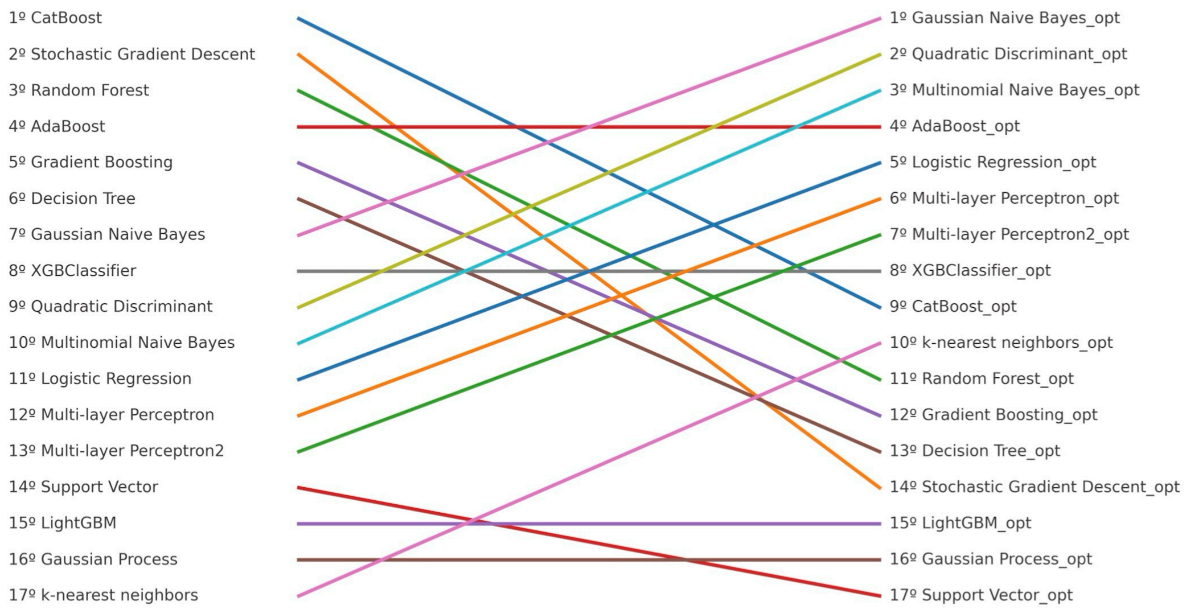

Figure S14. Classification of ML supervised models, with (first panel) and without (second panel) hyperparameter optimization, for CSTB.

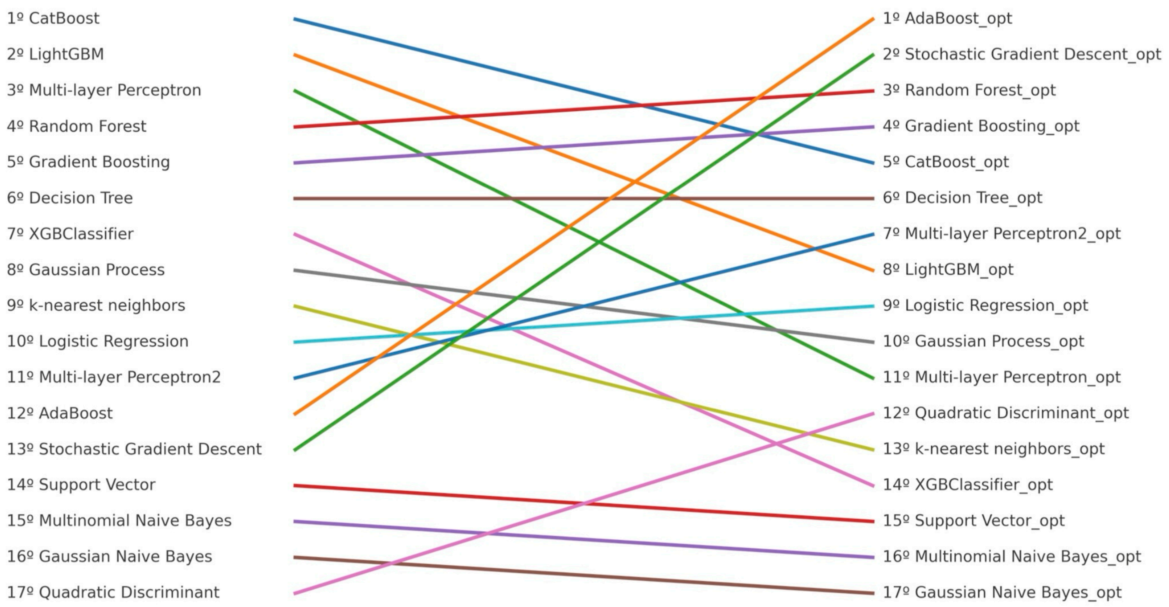

Figure S15. Classification of ML supervised models, with (first panel) and without (second panel) hyperparameter optimization, for the combined analysis.

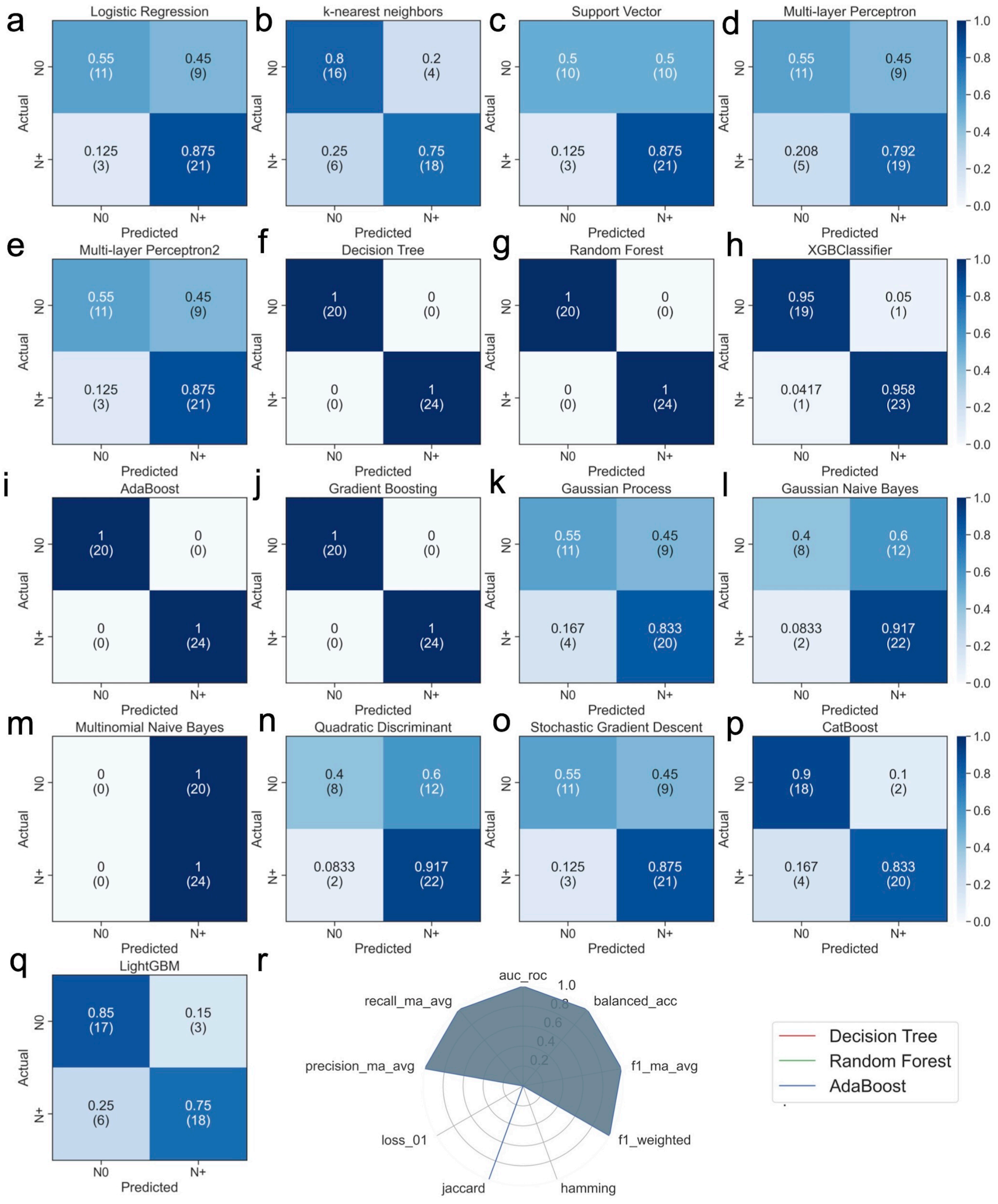

Figure S16. No optimized ML approaches considering the individual effect of the LTA4H biomarker for the training group. a-q, The confusion matrix was generated to closely examine true and false positives for all detection models. **r,** The best three classifiers are shown in the radar plot.

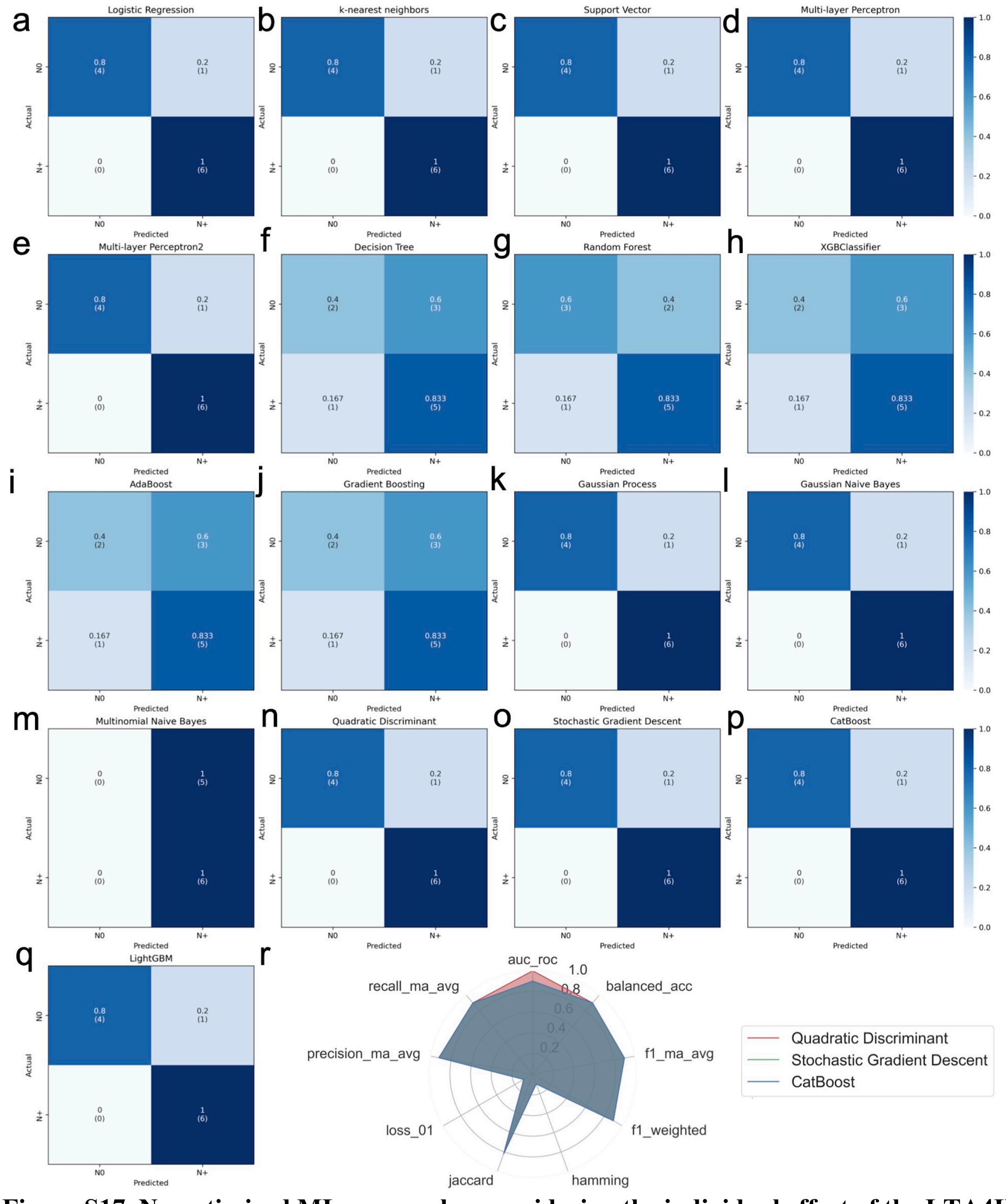

Figure S17. No optimized ML approaches considering the individual effect of the LTA4H biomarker for the test group. a-q, The confusion matrix was generated to closely examine true and false positives for all detection models. **r,** The best three classifiers are shown in the radar plot.

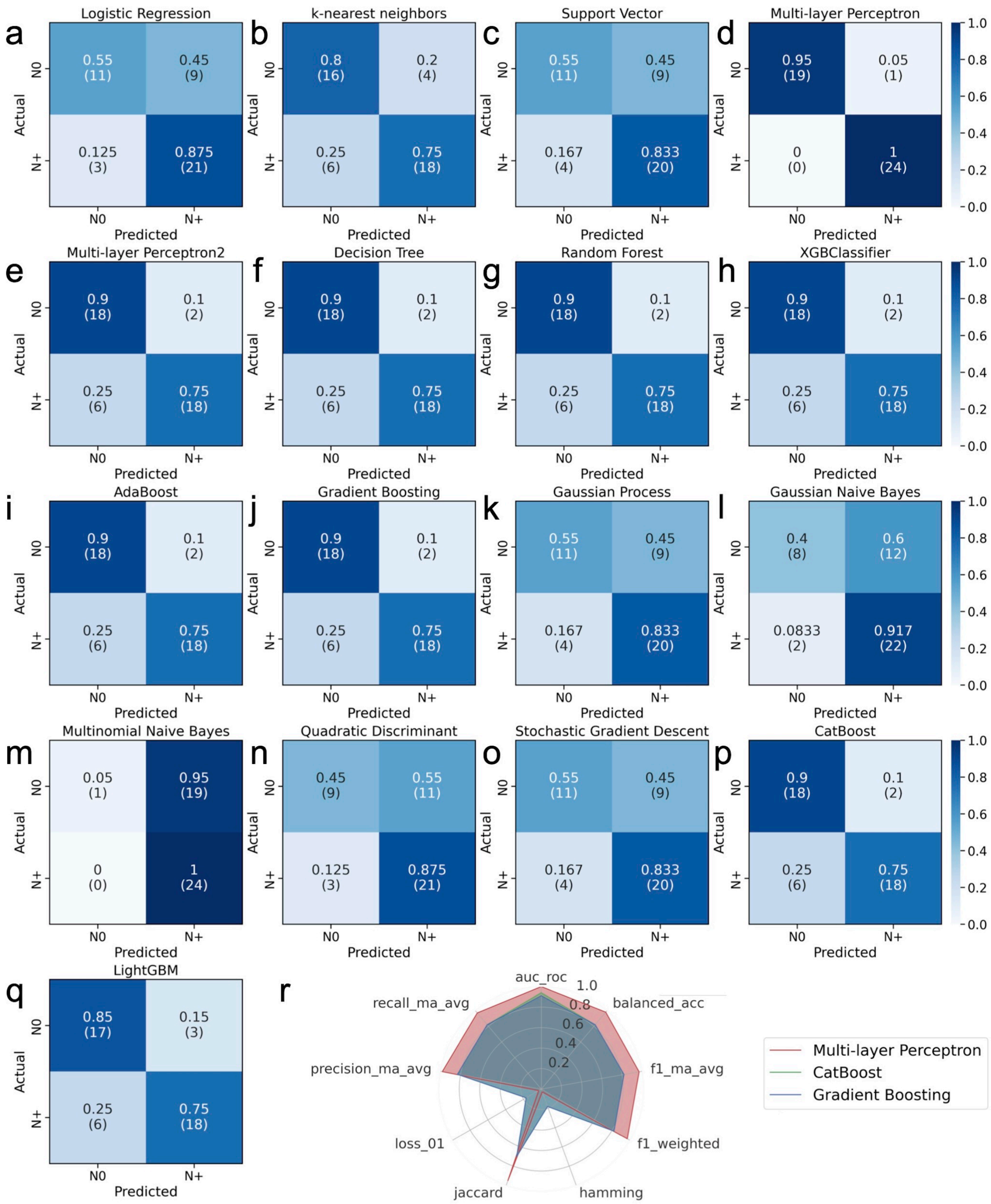

Figure S18. Optimized ML approaches considering the individual effect of LTA4H biomarker for the training group. a-q, The confusion matrix was generated to closely examine true and false positives for all detection models. **r,** The best three classifiers are shown in the radar plot.

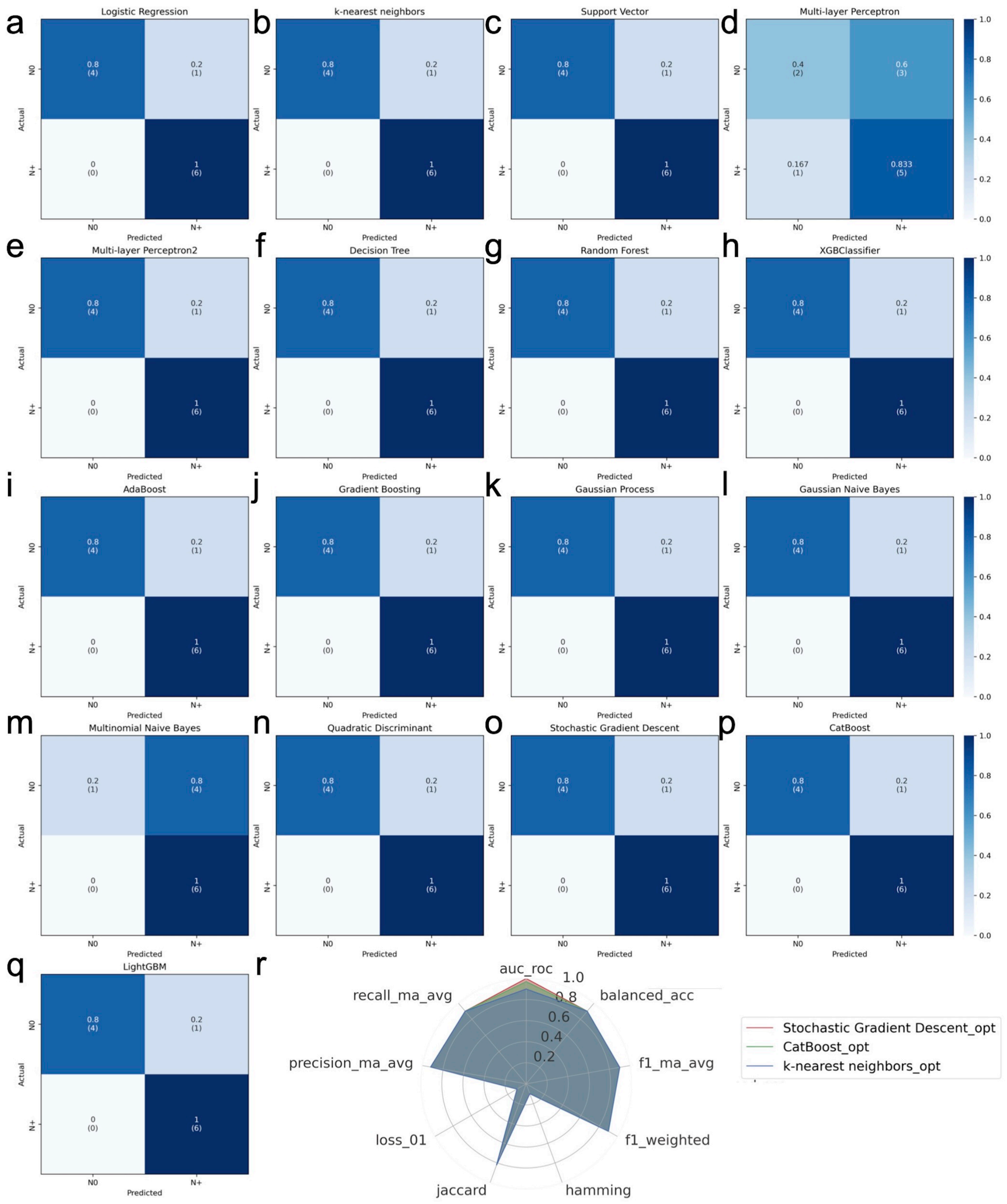

Figure S19. Optimized ML approaches considering the individual effect of LTA4H biomarker for the test group. a-q, The confusion matrix was generated to closely examine true and false positives for all detection models. **r,** The best three classifiers are shown in the radar plot.

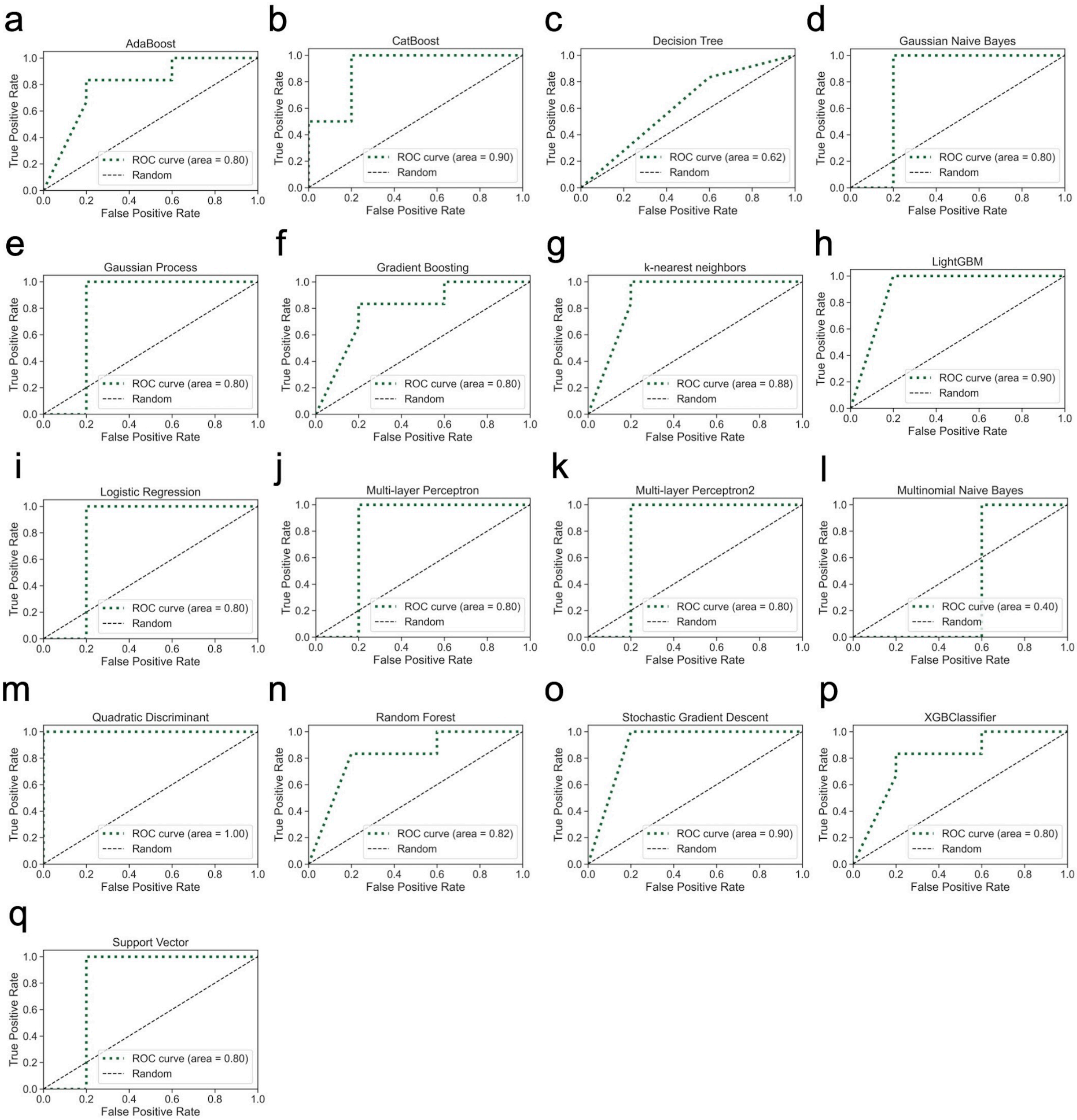

Figure S20. AUC-ROC curves for the non-optimized ML approaches for LTA4H. a-q, AUC-ROC curves considering the individual effect of LTA4H biomarker. The (**m**) QD model was the best classifier for LTA4H.

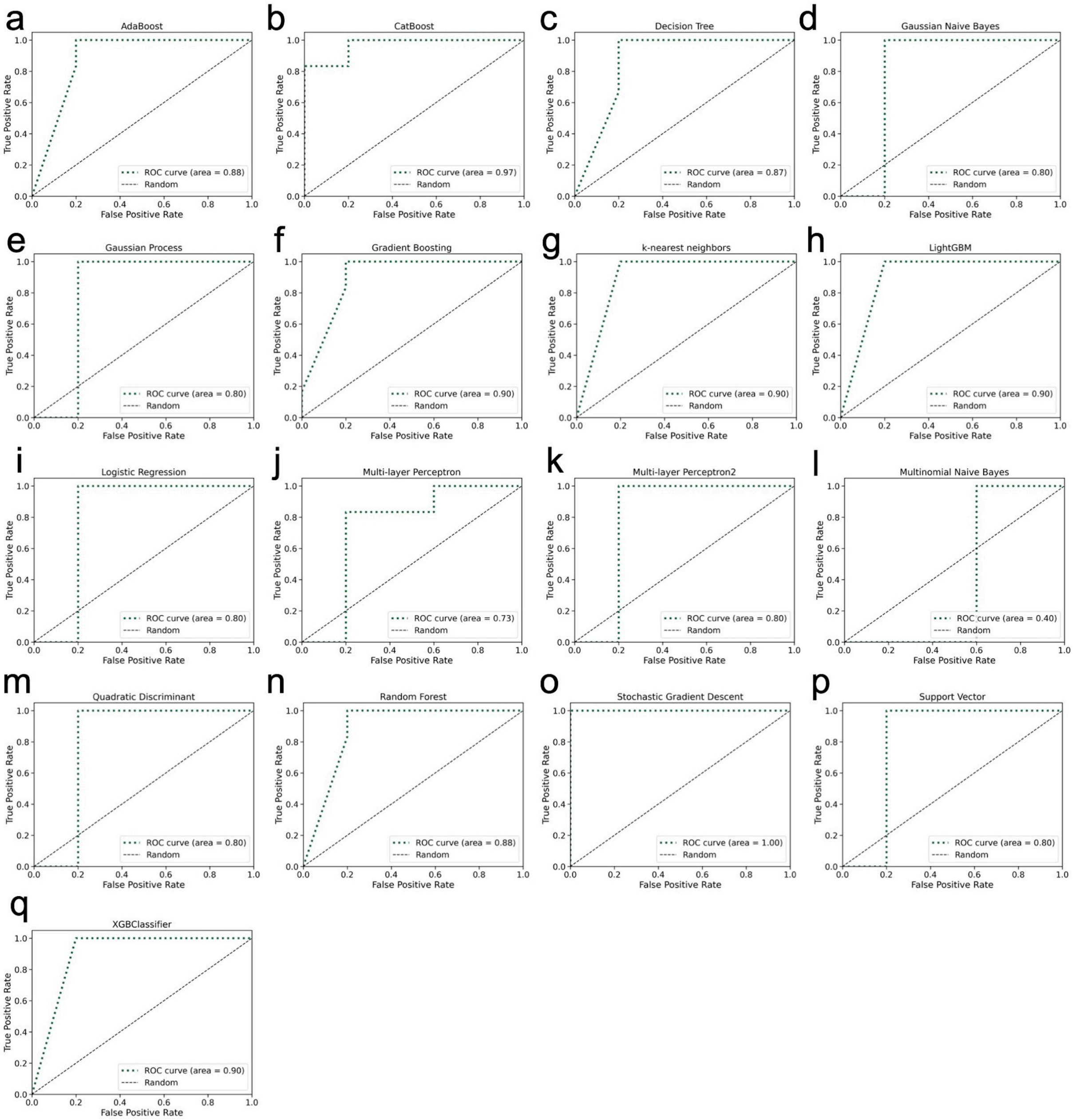

Figure S21. AUC-ROC curves for the optimized ML approaches for LTA4H. a-q, AUC-ROC curves considering the individual effect of LTA4H biomarker. **b,** The CB and (**o**) SGD models are in the top three classifiers for LTA4H.

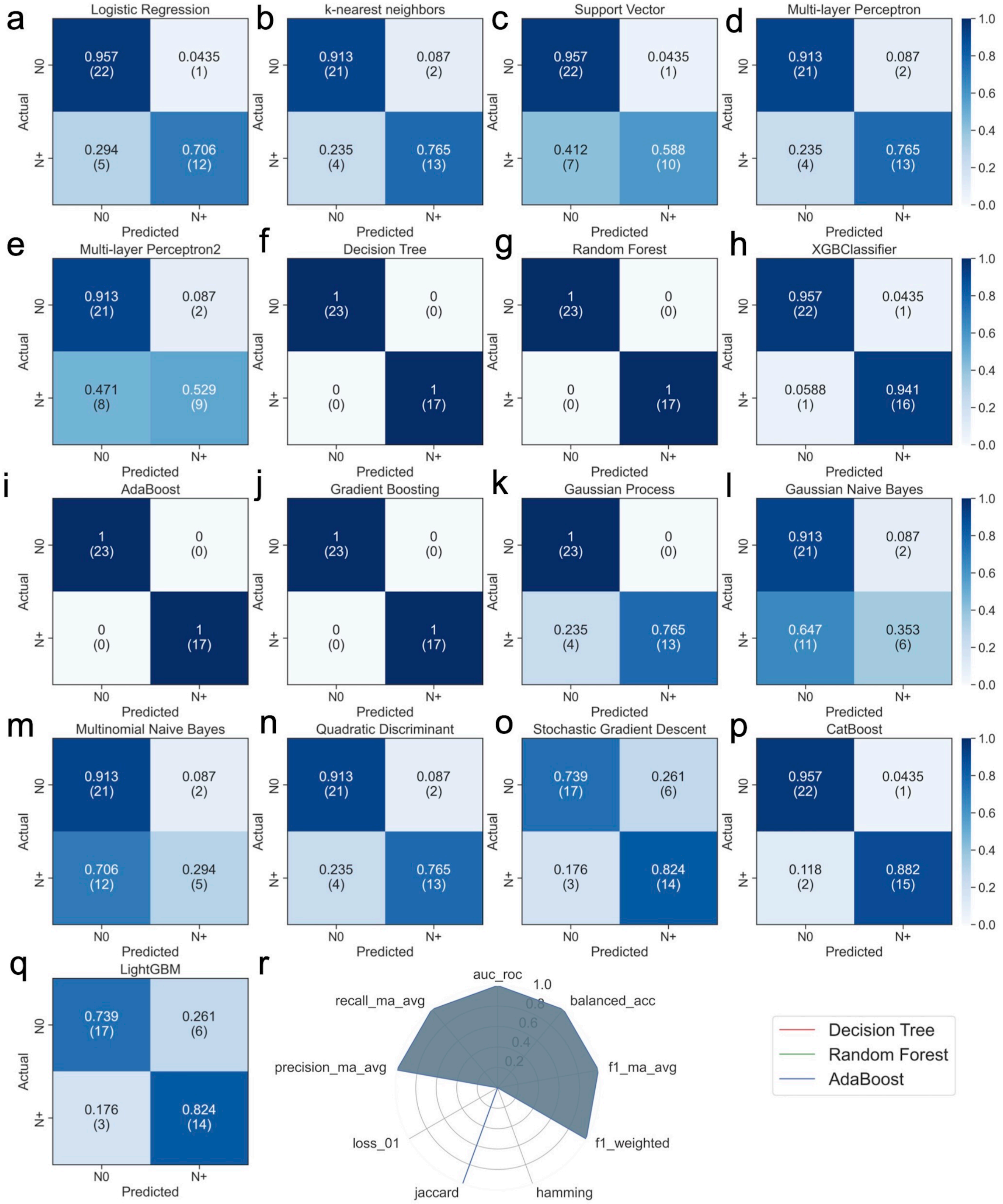

Figure S22. No optimized ML approaches considering the individual effect of COL6A1 biomarker for the training group. a-q, The confusion matrix was generated to closely examine true and false positives for all detection models. **r,** The best three classifiers are shown in the radar plot (R).

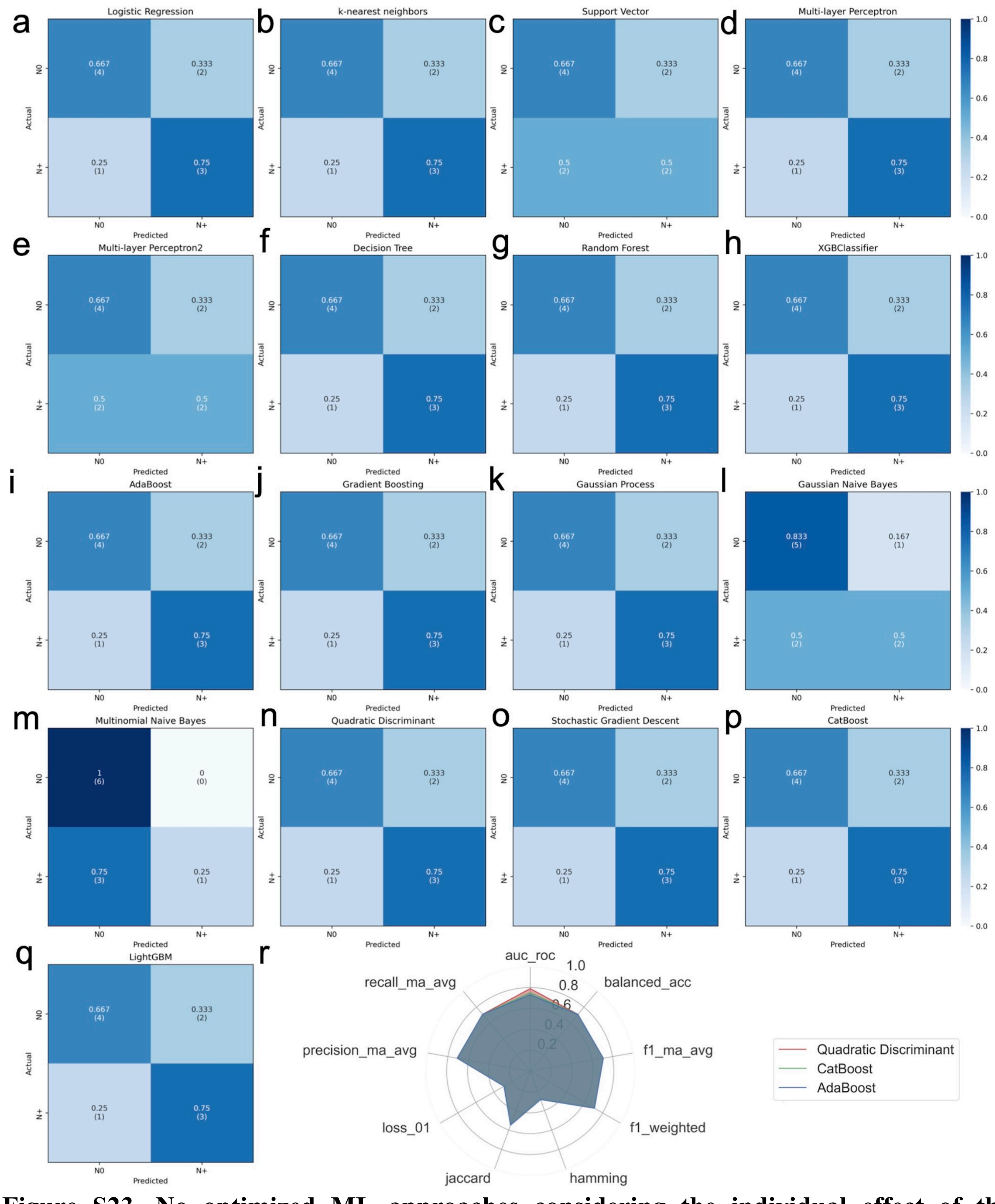

Figure S23. No optimized ML approaches considering the individual effect of the COL6A1 biomarker for the test group. a-q, The confusion matrix was generated to closely examine true and false positives for all detection models. **r,** The best three classifiers are shown in the radar plot.

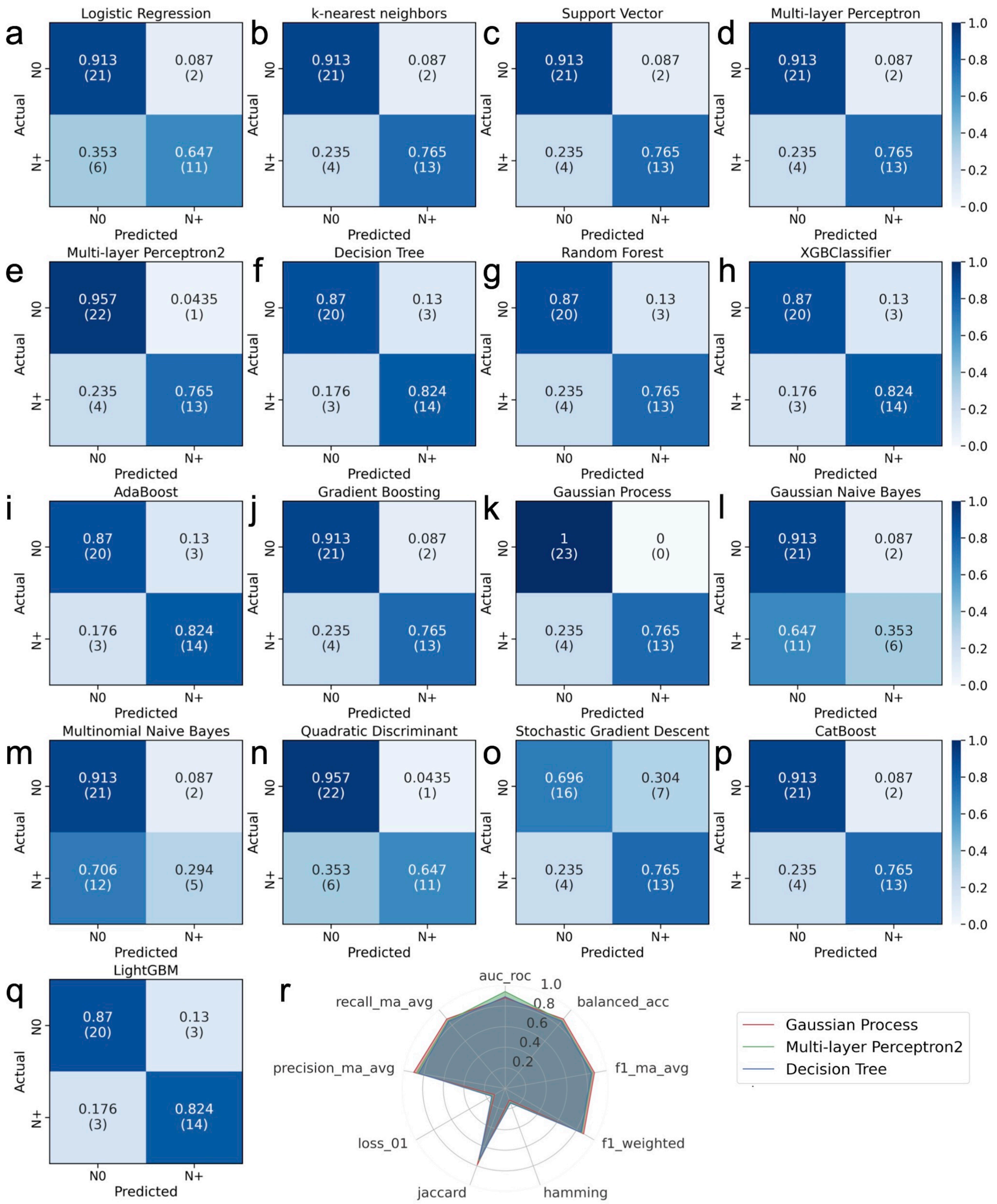

Figure S24. Optimized ML approaches considering the individual effect of COL6A1 biomarker for the training group. a-q, The confusion matrix was generated to closely examine true and false positives for all detection models. **r,** The best three classifiers are shown in the radar plot.

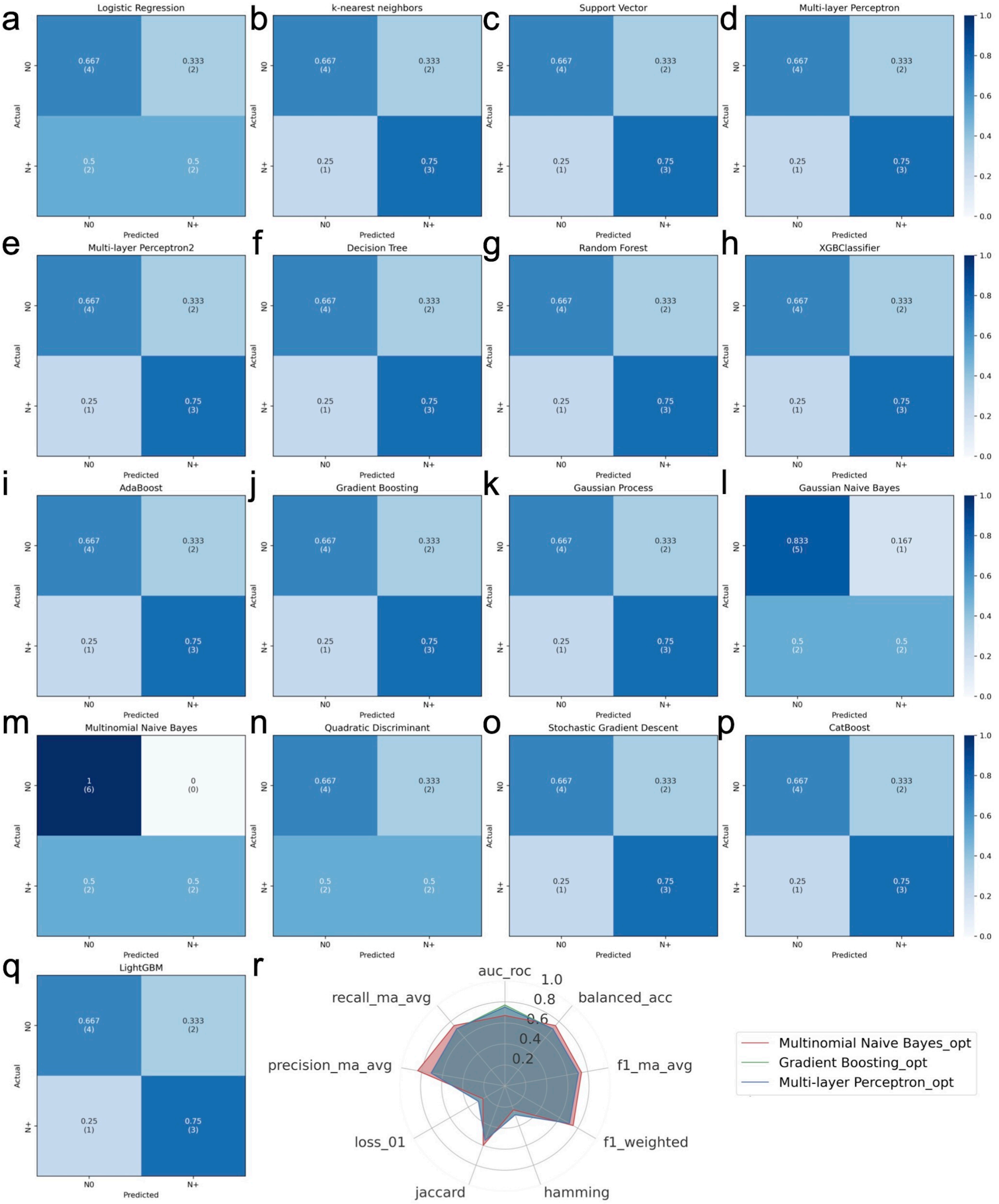

Figure S25. Optimized ML approaches considering the individual effect of COL6A1 biomarker for the test group. a-q, The confusion matrix was generated to closely examine true and false positives for all detection models. **r,** The best three classifiers are shown in the radar plot.

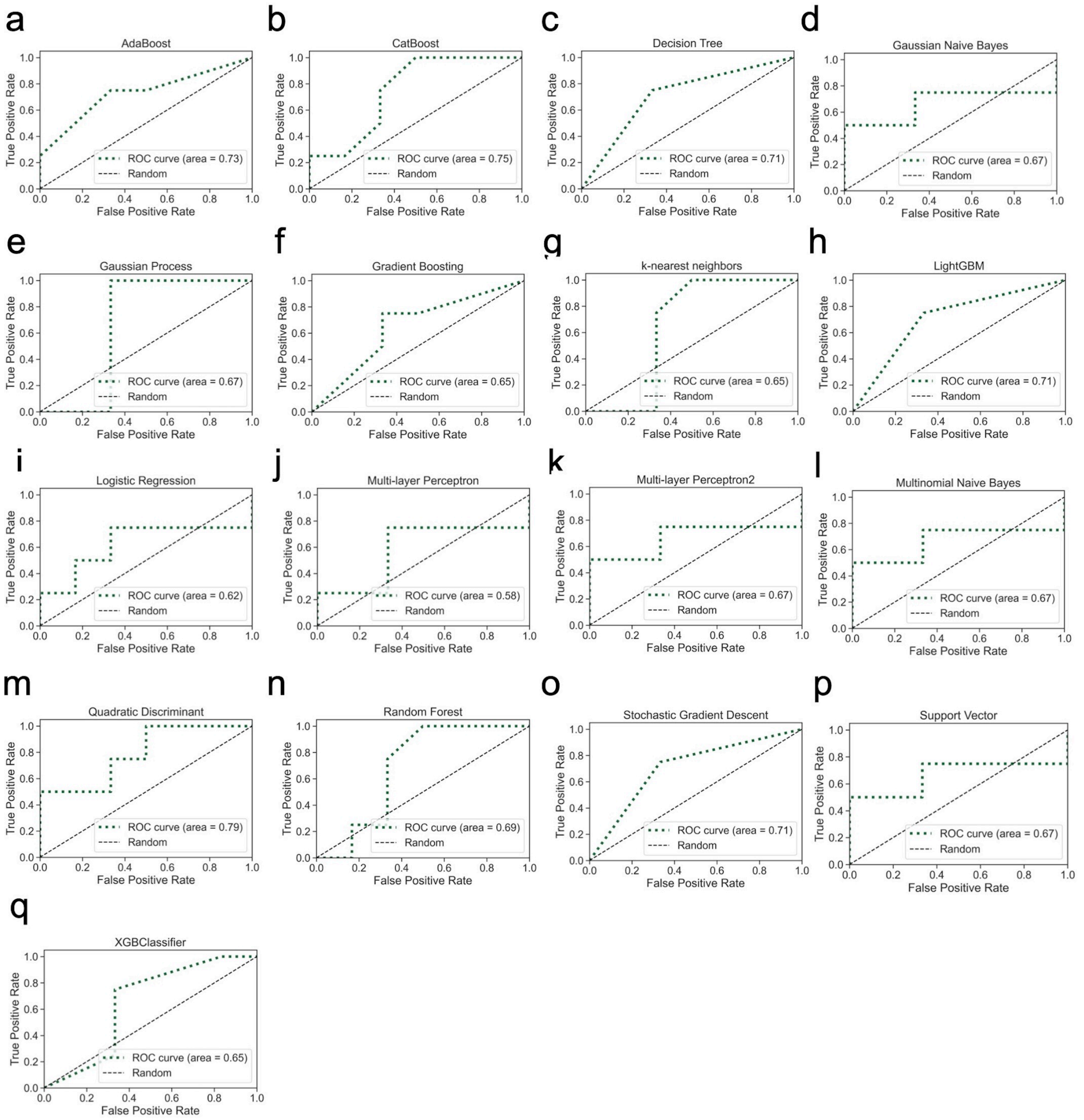

Figure S26. AUC-ROC curves for the non-optimized ML approaches for COL6A1. a-q, AUC-ROC curves considering the individual effect of COL6A1 biomarker.

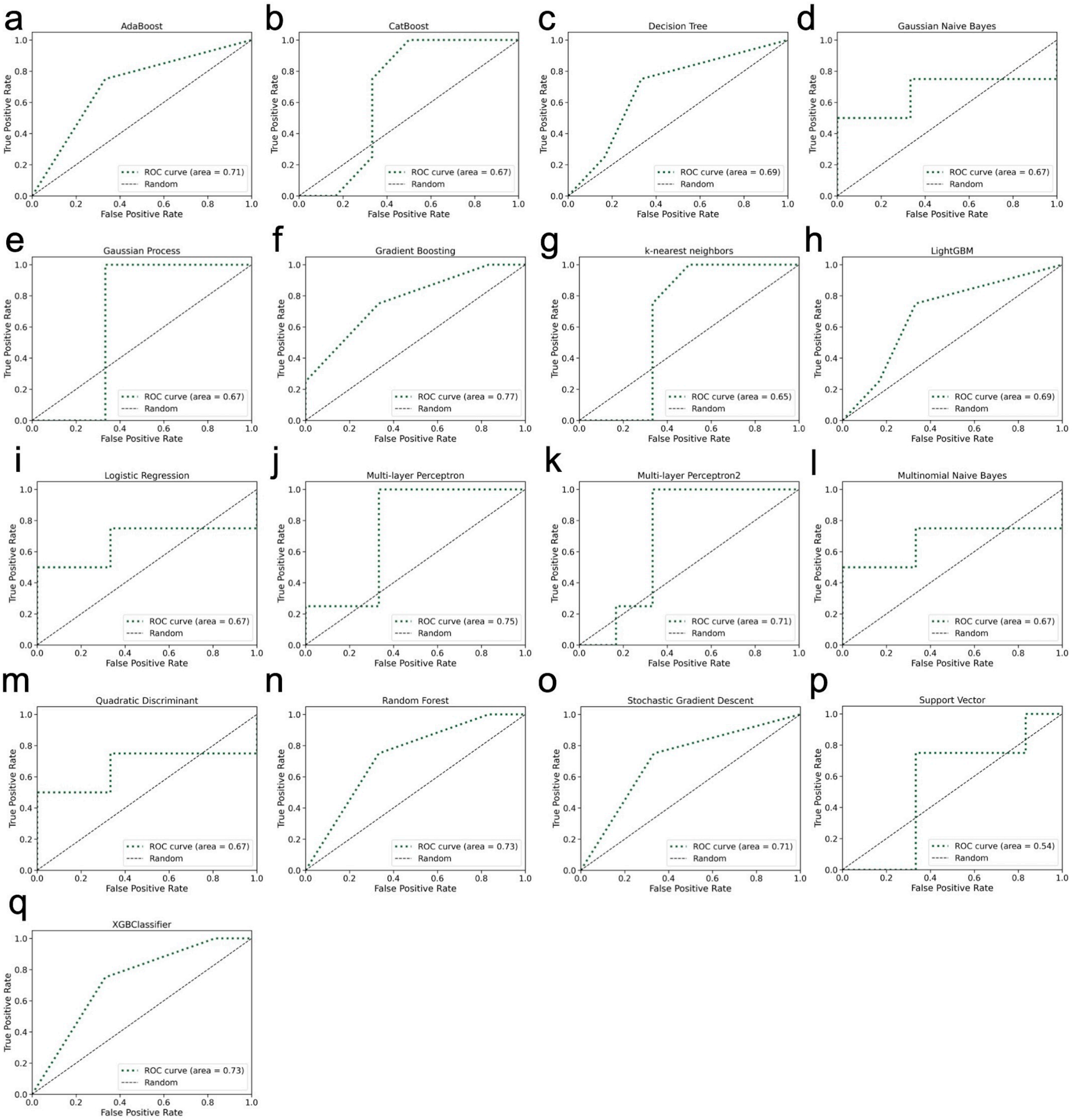

Figure S27. AUC-ROC curves for the optimized ML approaches for COL6A1. a-q, AUC-ROC curves considering the individual effect of COL6A1 biomarker. The (**f**) GB, (**l**) MNB, and (**m**) QD models are the top three classifiers for COL6A1.

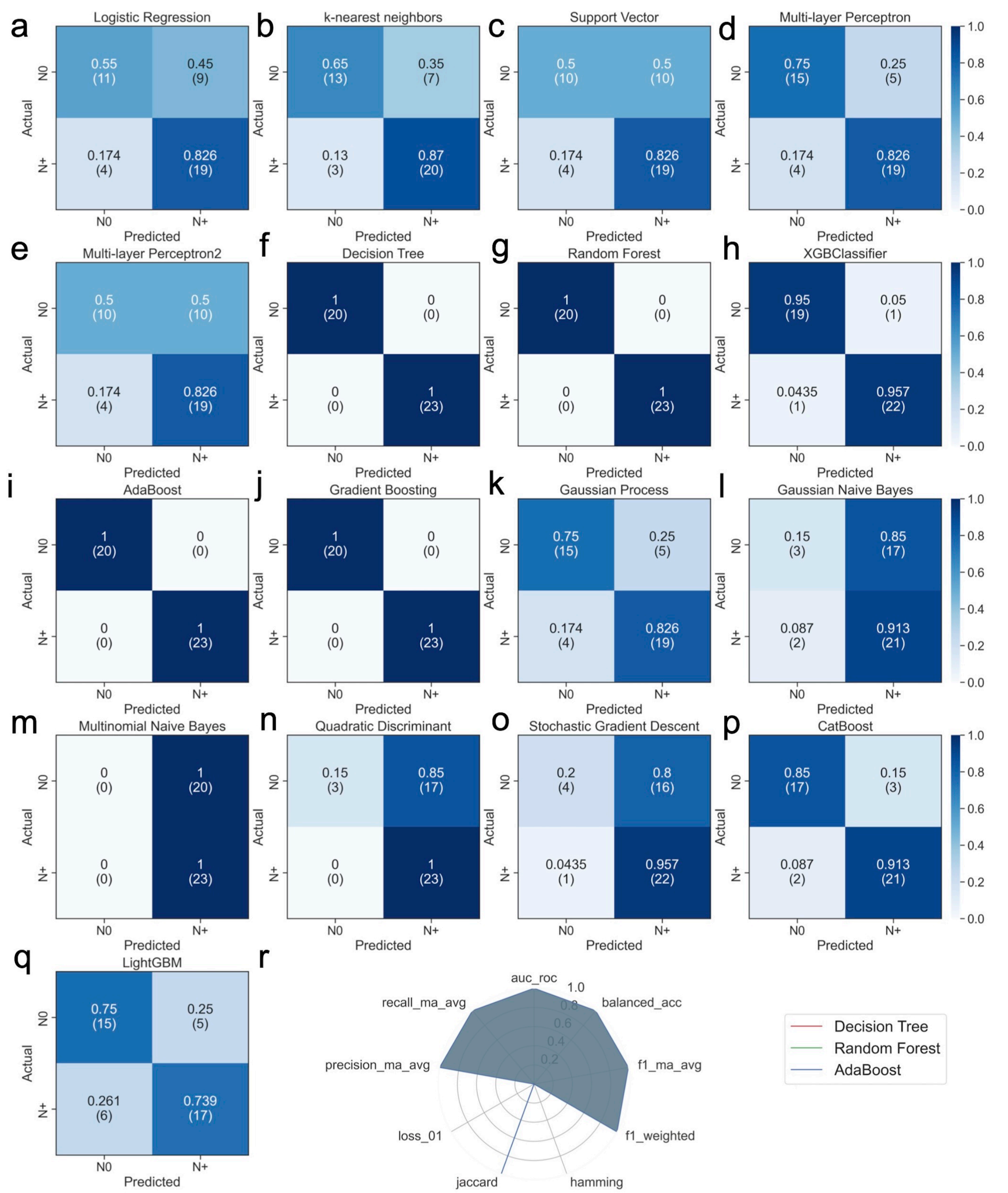

Figure S28. No optimized ML approaches considering the individual effect of CSTB biomarker for the training group. a-q, The confusion matrix was generated to closely examine true and false positives for all detection models. **r,** The best three classifiers are shown in the radar plot.

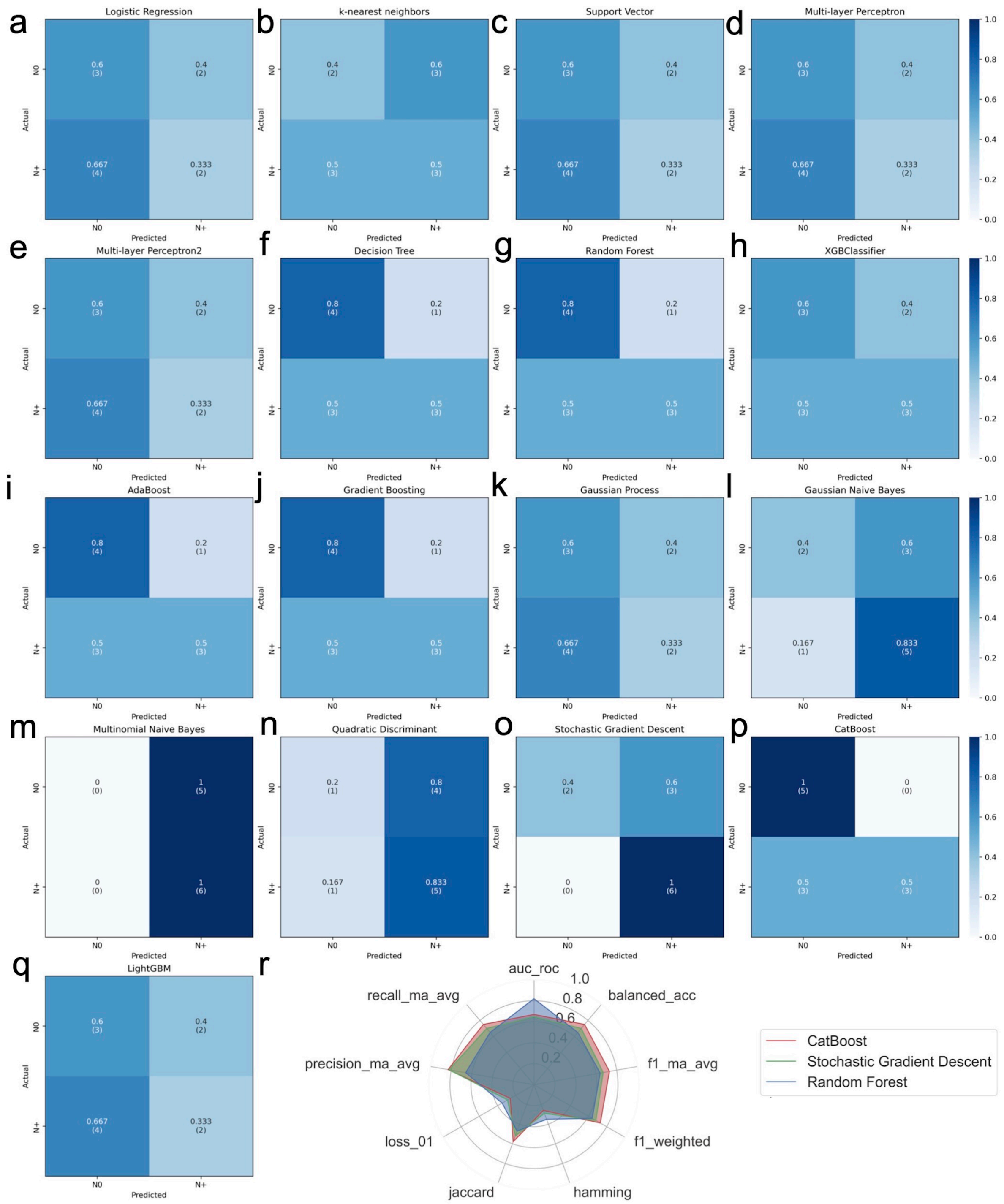

Figure S29. No optimized ML approaches considering the individual effect of CSTB biomarker for the test group. a-q, The confusion matrix was generated to closely examine true and false positives for all detection models. **r,** The best three classifiers are shown in the radar plot.

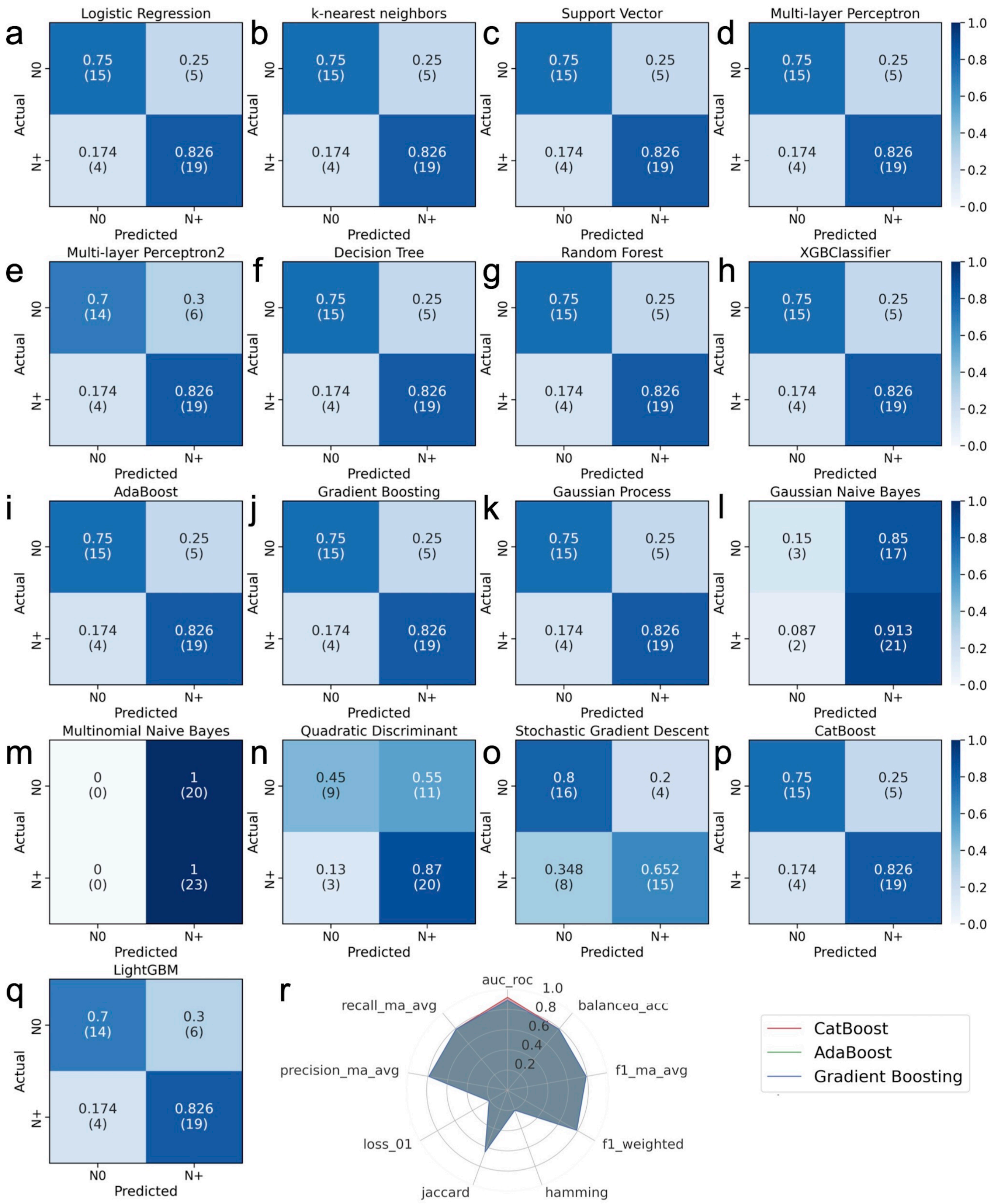

Figure S30. Optimized ML approaches considering the individual effect of CSTB biomarker for the training group. a-q, The confusion matrix was generated to closely examine true and false positives for all detection models. **r,** The best three classifiers are shown in the radar plot.

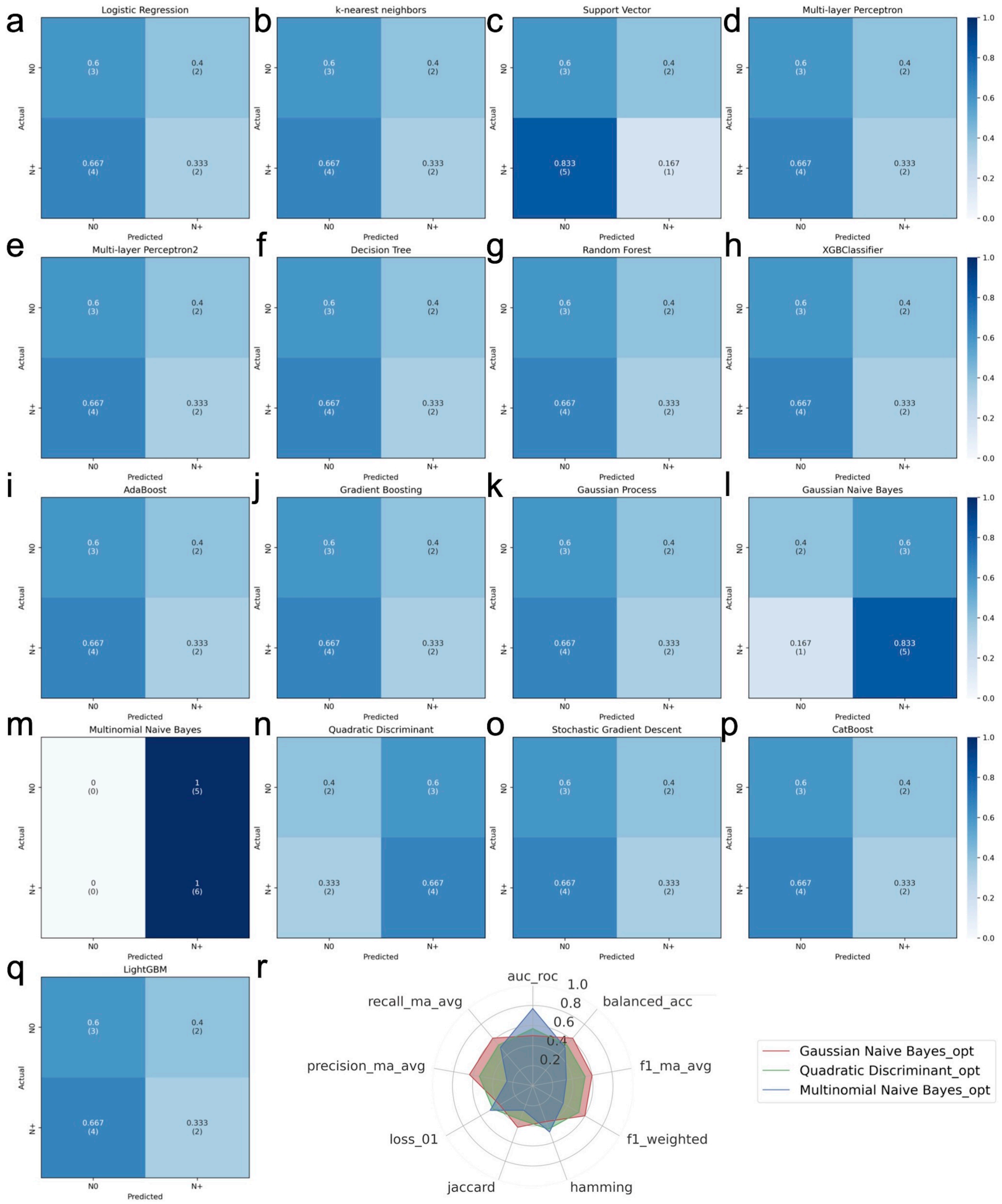

Figure S31. Optimized ML approaches considering the individual effect of CSTB biomarker for the test group. a-q, The confusion matrix was generated to closely examine true and false positives for all detection models. **r,** The best three classifiers are shown in the radar plot.

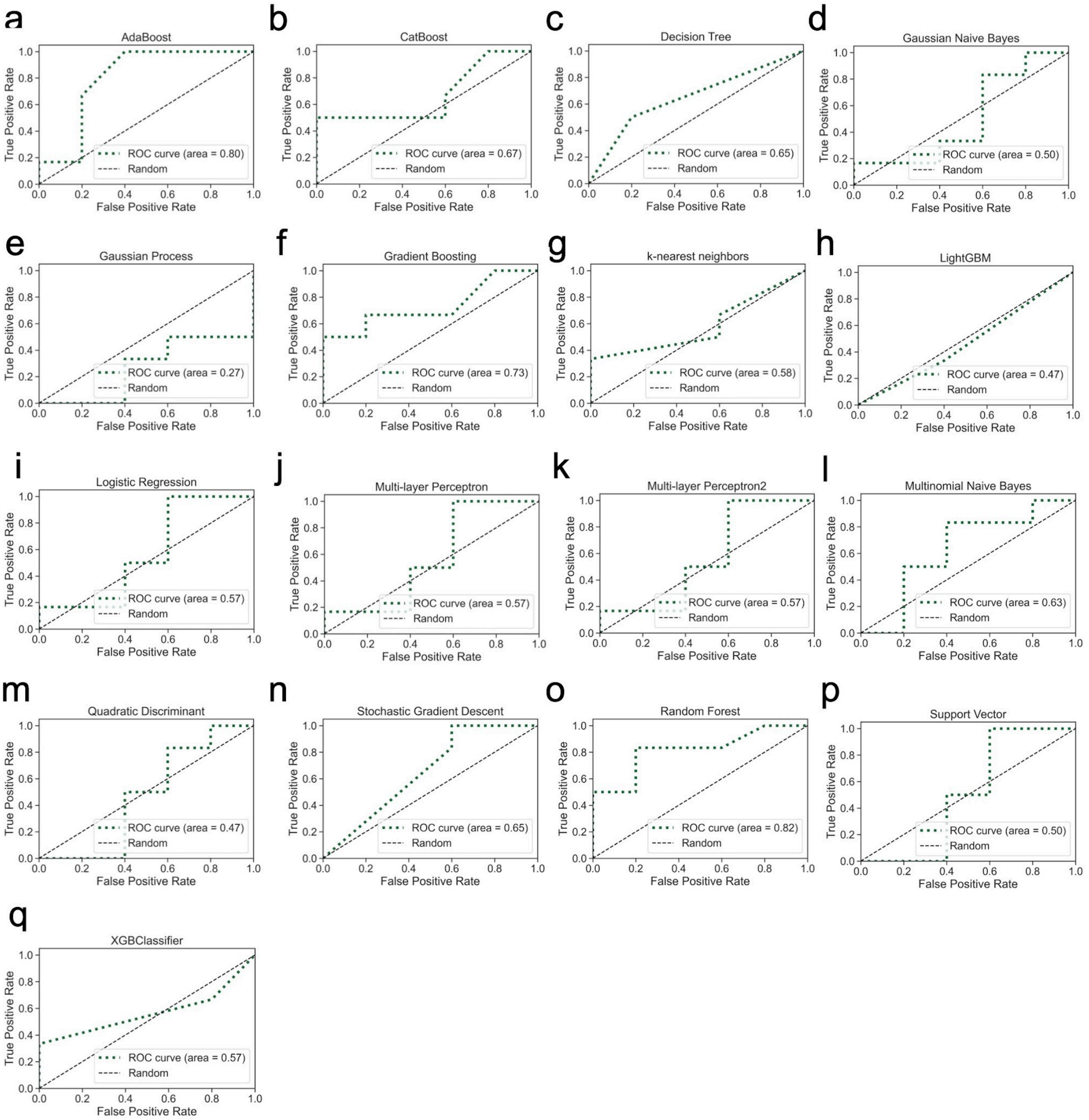

Figure S32. AUC-ROC curves for the non-optimized ML approaches for CSTB. a-q, AUC-ROC curves considering the individual effect of CSTB biomarker. The (**b**) CB, (**n**) SGD and (**o**) RF models are the top three classifiers for CSTB.

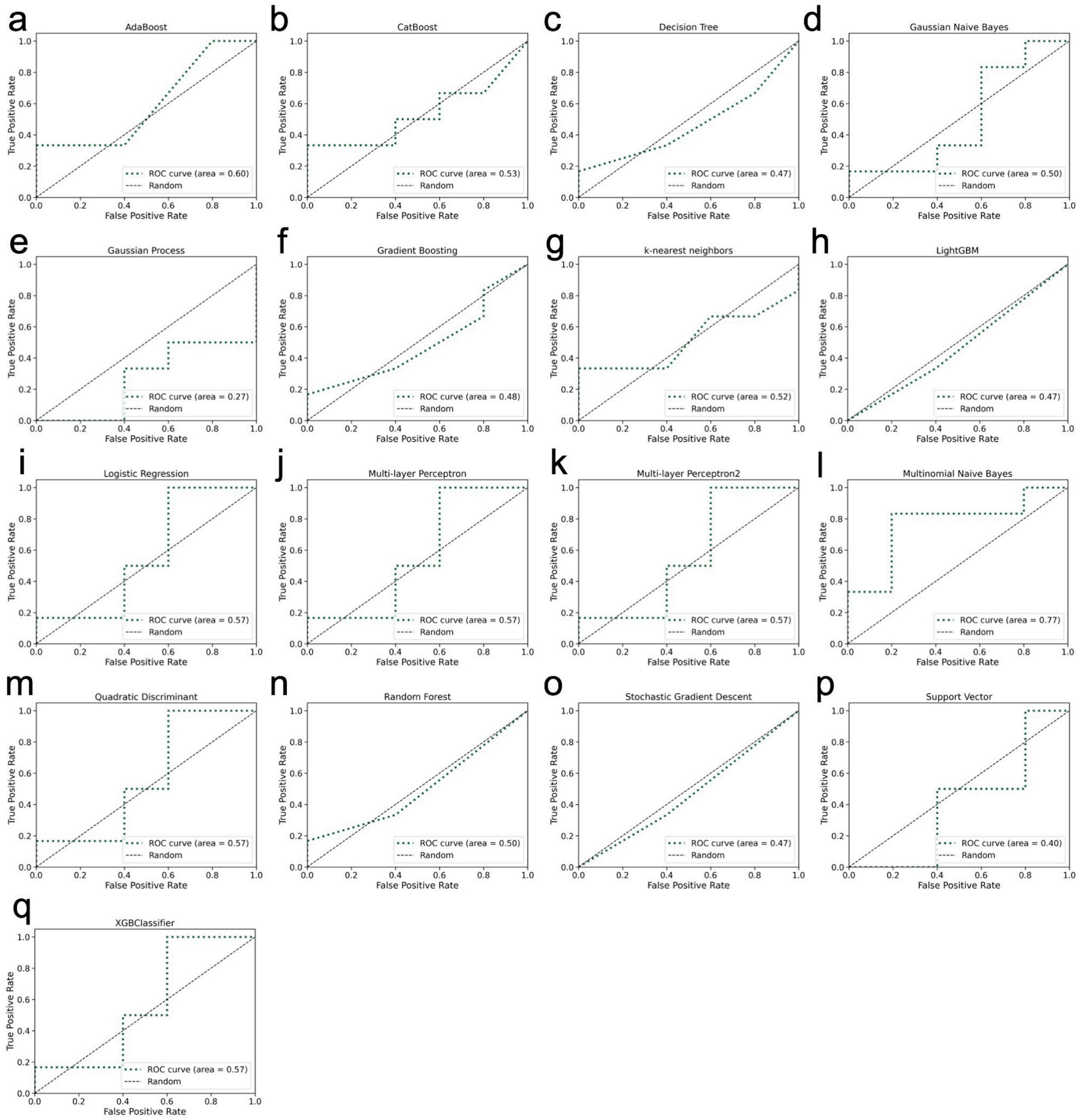

Figure S33. AUC-ROC curves for the optimized ML approaches for CSTB. a-q, AUC-ROC curves considering the individual effect of CSTB biomarker.

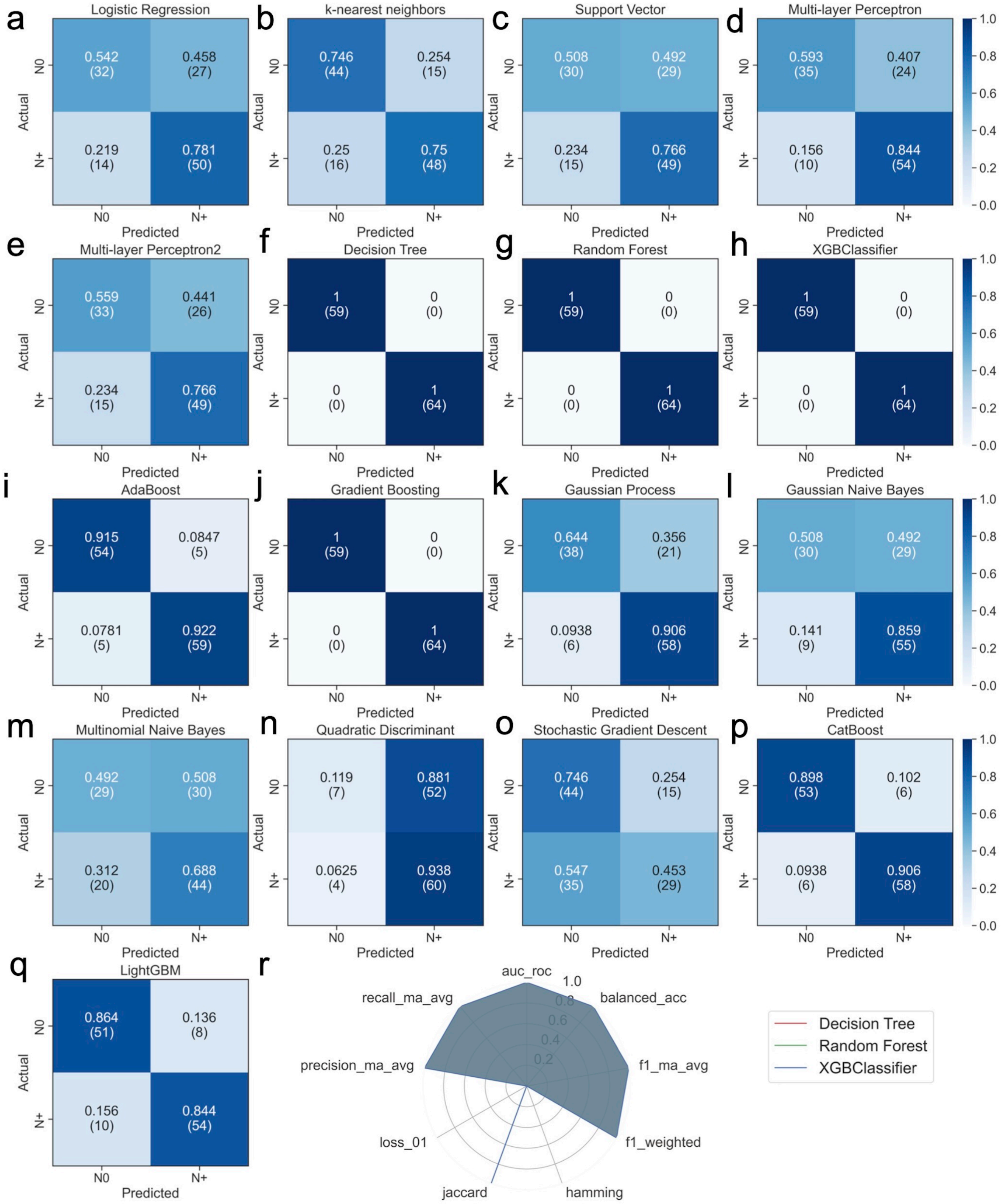

Figure S34. No optimized ML approaches considering the combined effect of the biomarkers for the training group. a-q, The confusion matrix was generated to closely examine true and false positives for all detection models. **r,** The best three classifiers are shown in the radar plot.

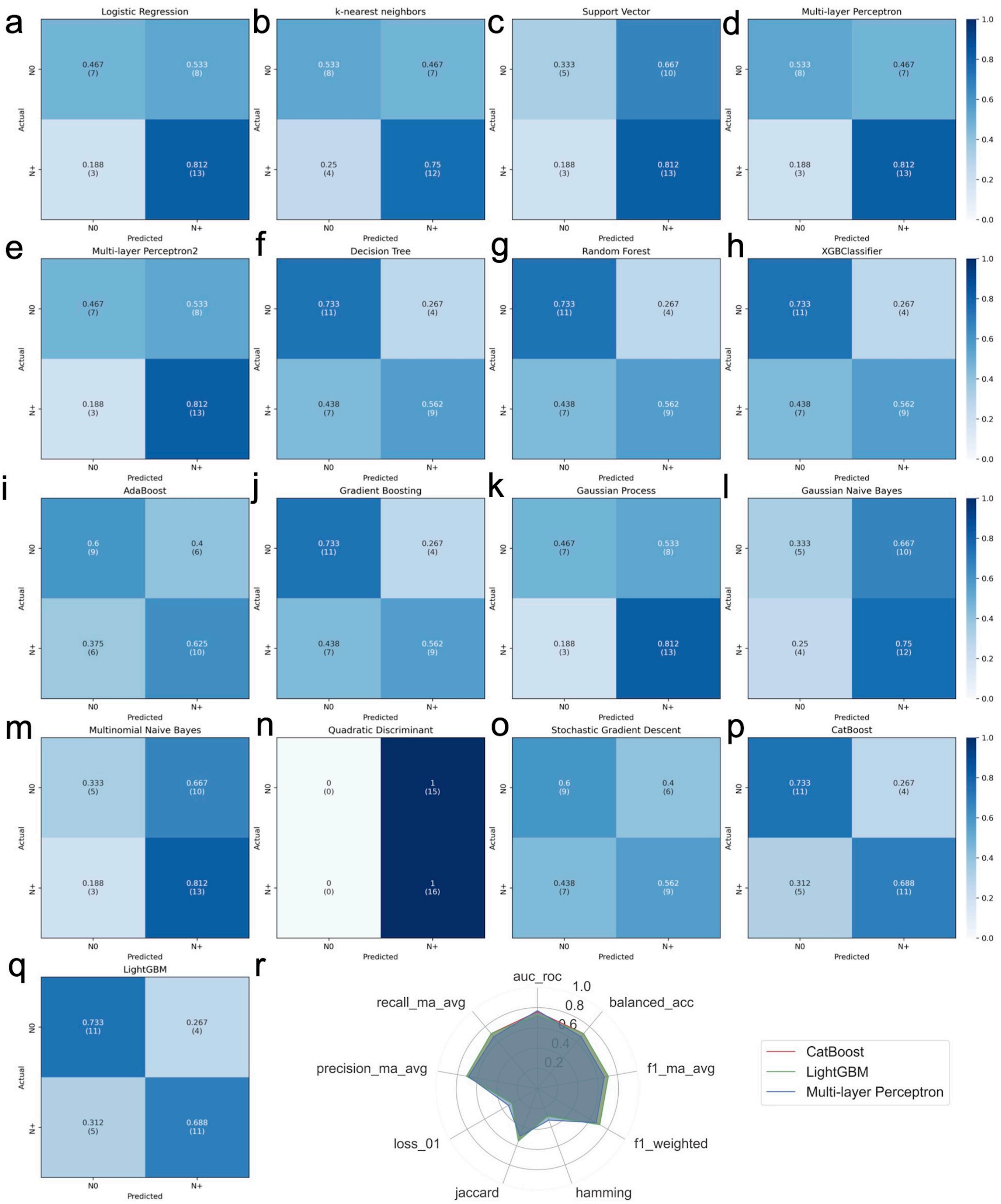

Figure S35. No optimized ML approaches considering the combined effect of the biomarkers for the test group. a-q, The confusion matrix was generated to closely examine true and false positives for all detection models. **r,** The best three classifiers are shown in the radar plot.

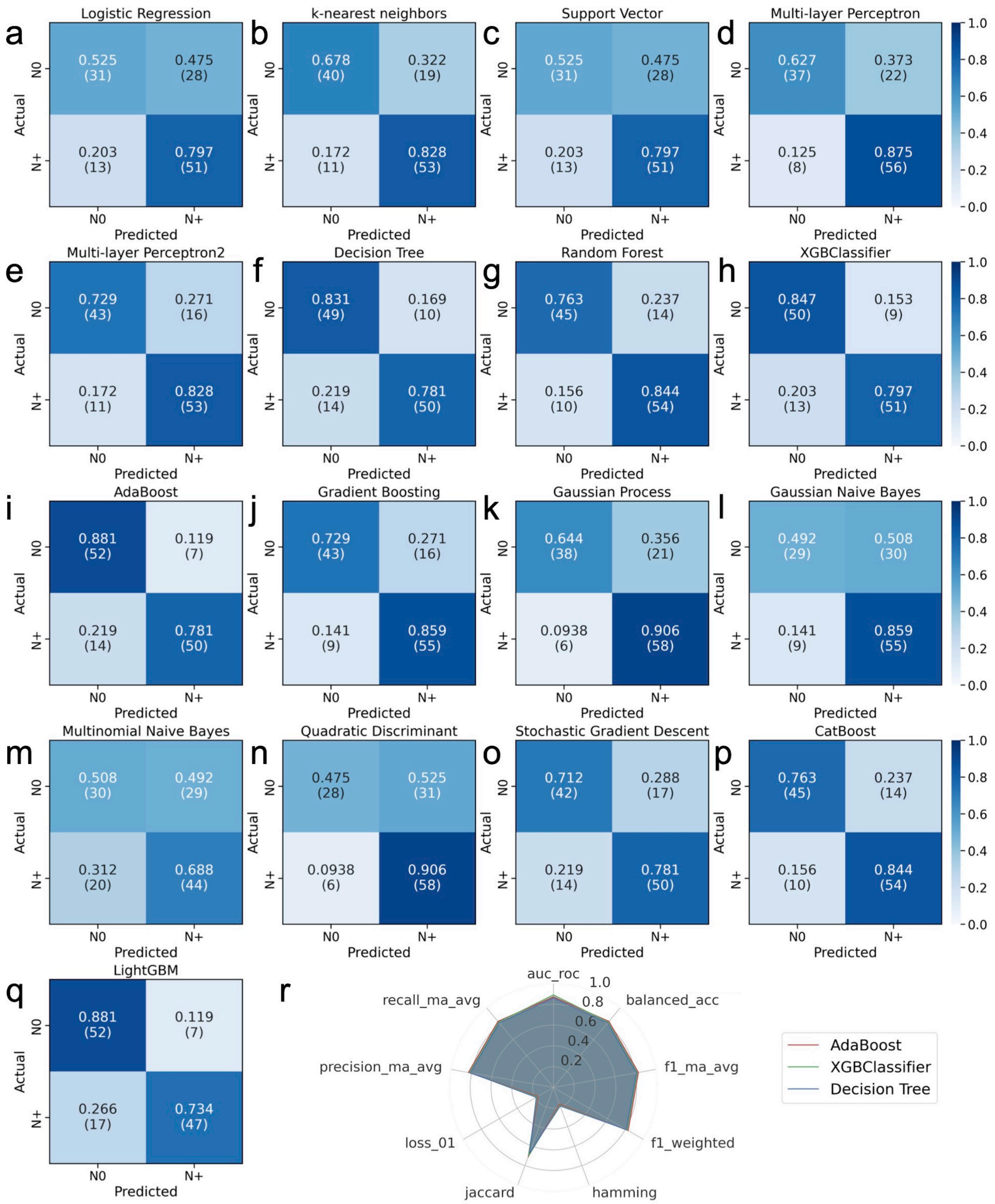

Figure S36. Optimized ML approaches considering the combined effect of the biomarkers for the training group. a-q, The confusion matrix was generated to closely examine true and false positives for all detection models. **r,** The best three classifiers are shown in the radar plot.

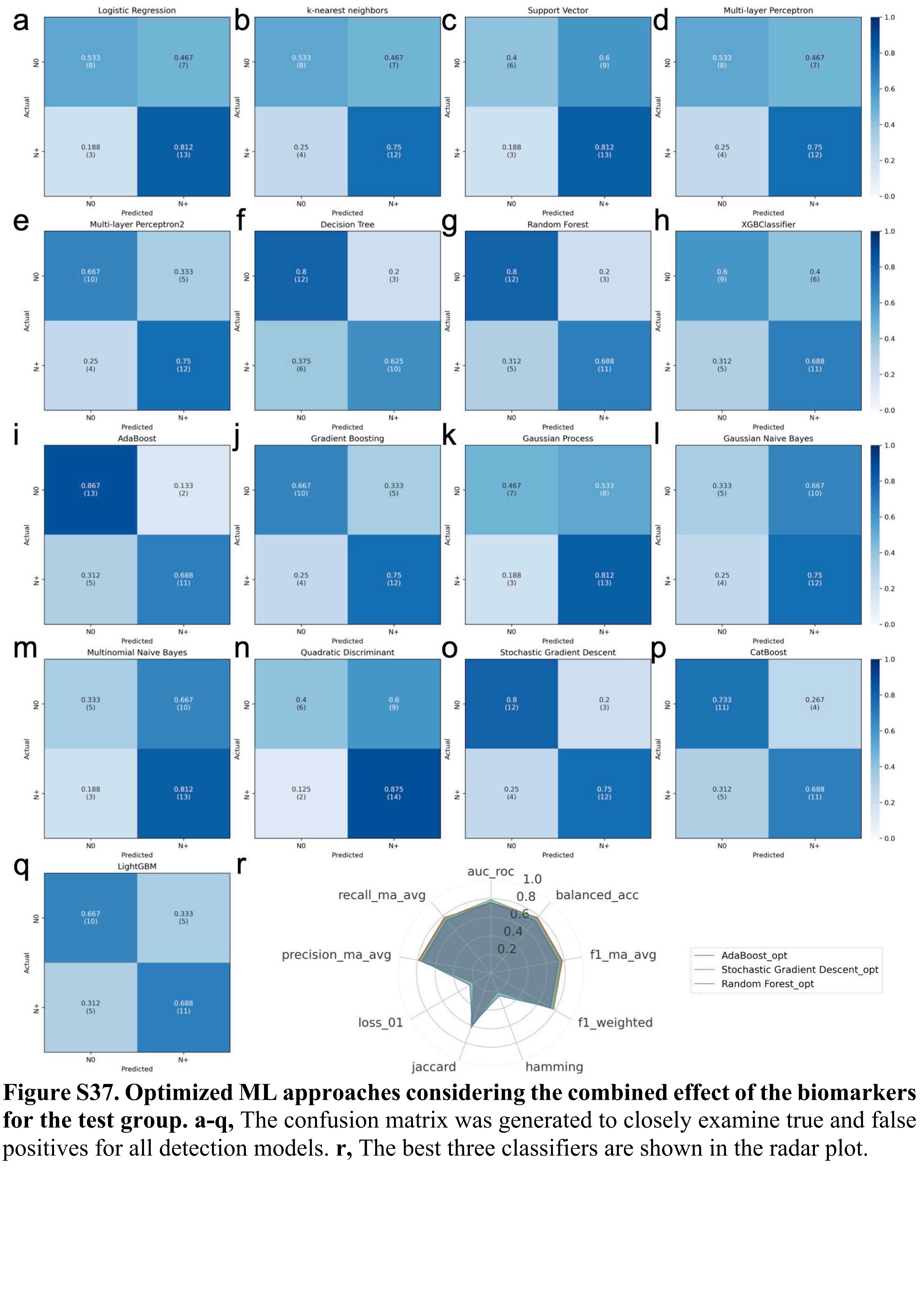

Figure S37. Optimized ML approaches considering the combined effect of the biomarkers for the test group. a-q, The confusion matrix was generated to closely examine true and false positives for all detection models. **r,** The best three classifiers are shown in the radar plot.

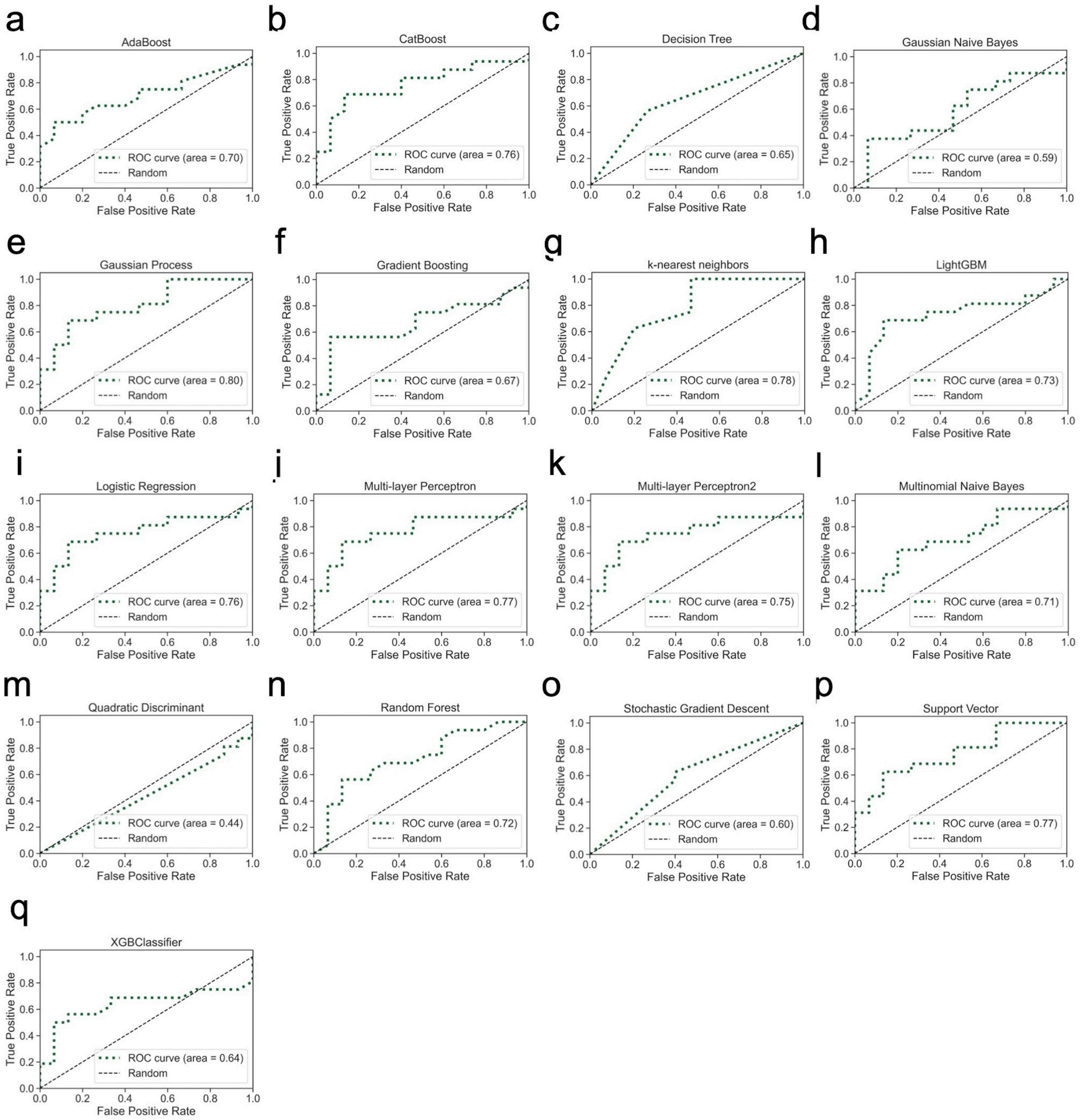

Figure S38. AUC-ROC curves for the non-optimized ML approaches for all the biomarkers. a-q, AUC-ROC curves considering the combined effect of the biomarkers.

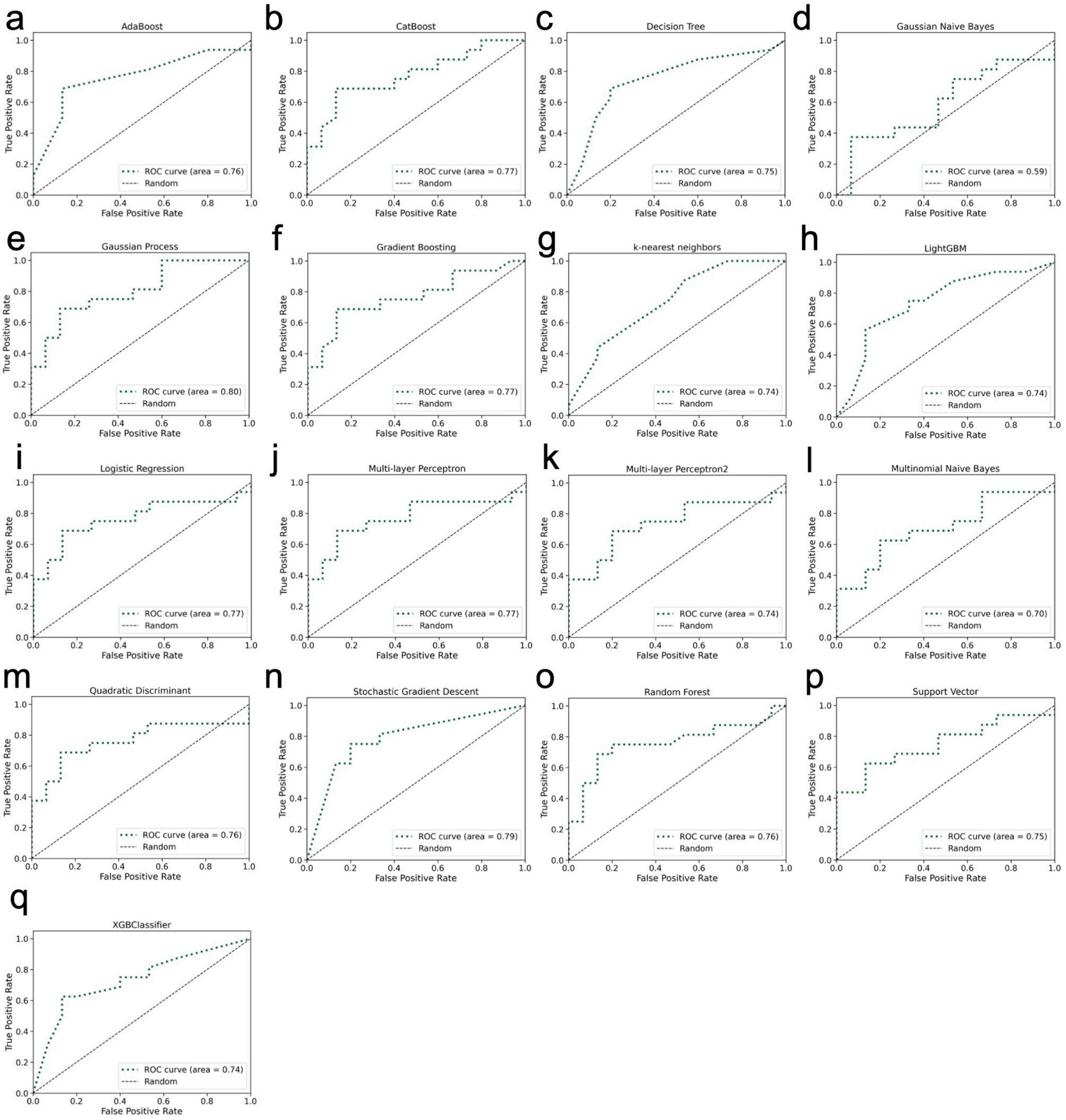

Figure S39. AUC-ROC curves for the optimized ML approaches for all the biomarkers. a-q, AUC-ROC curves considering the combined effect of the biomarkers. The (**a**) AB, (**n**) SGD and (**o**) RF models are the top three classifiers for the combined effect of LTA4H, COL6A1, and CSTB.

Table S2. Clinical and demographic data of OSCC patient samples included in the analysis.

Prognosis	Age of diagnosis	Gender	Tumor diagnosis	TNM Classification	Exposure to tobacco	Average number of cigarettes consumed per day	Years of exposure to tobacco	Exposure to alcohol	Average volume of alcohol consumed per day (mL)	Years of exposure to alcohol
	65	Male	Gum	T4aN0M0	Ex-smoker	20	11	Ex-alcoholic	500	11
	62	Female	Floor of the mouth	T4aN0M0	Ex-smoker	5	42	Never drank	-	-
	59	Male	Retromolar area	T4N0M0	Ex-smoker	20	45	Ex-alcoholic	1000	44
	51	Male	Buccal mucosa	T3N0M0	Never smoked	-	-	Ex-alcoholic	150	10
	65	Male	Buccal mucosa	T2N0M0	NA	NA	NA	NA	NA	NA
	67	Male	Floor of the mouth	T4aN0M0	NA	NA	NA	NA	NA	NA
	77	Female	Retromolar area	T4aN0M0	NA	NA	NA	NA	NA	NA
	53	Male	Palate	T2N0M0*	NA	NA	NA	NA	NA	NA
	59	Male	Tongue	T3N0M0	Current smoker	40	51	Ex-alcoholic	2500	25
	59	Male	Tongue	T2N0M0	Never smoked	-	-	NA	NA	NA
	91	Male	Tongue	T3N0M0	NA	NA	NA	NA	NA	NA
	74	Male	Tongue	T2N0M0*	NA	NA	NA	NA	NA	NA
	44	Male	Retromolar area	T4N0Mx*	Ex-smoker	20	39	NA	NA	NA
	55	Male	Tongue	T1N0M0	Ex-smoker	20	30	Ex-alcoholic	-	-
	80	Male	Alveolar ridge	T4aN0M0	NA	NA	NA	NA	NA	NA
	58	Male	Tongue	T1N0M0	NA	NA	NA	NA	NA	NA
N0	66	Male	Buccal mucosa	T1N0M0	Ex-smoker	23	58	NA	NA	NA
	39	Female	Tongue	T4aN0M0	Never smoked	-	-	Never drank	-	-
	74	Female	Tongue	T1N0M0	NA	NA	NA	NA	NA	NA
	72	Male	Floor of the mouth	T2N0M0	NA	NA	NA	NA	NA	NA
	61	Male	Tongue	T4aN0M0	Ex-smoker	5	43	Ex-alcoholic	570	43
	71	Female	Alveolar ridge	T4aN0M0	Ex-smoker	20	45	Ex-alcoholic	500	30
	39	Female	Tongue	T2N0M0	Ex-smoker	5	20	Ex-alcoholic	-	-
	62	Male	Floor of the mouth	T2N0M0	Ex-smoker	80	NA	Ex-alcoholic	500	10
	59	Male	Floor of the mouth	T2N0M0	NA	NA	NA	NA	NA	NA
	69	Male	Floor of the mouth	T4N0M0*	Current smoker	20	59	Ex-alcoholic	1500	52
	64	Male	Amygdala	T2N0M0*	NA	NA	NA	NA	NA	NA
	55	Male	Floor of the mouth	T2N0Mx	Ex-smoker	30	40	Ex-alcoholic	2000	16
	71	Male	Tongue	T3N0M0	NA	NA	NA	NA	NA	NA
	81	Male	Retromolar area	T4N0M0*	Ex-smoker	15	28	NA	NA	NA
	67	Male	Palate	T4N2bM0	Ex-smoker	30	41	NA	NA	NA
	59	Male	Tongue	T4N3M0	Current smoker	20	45	Ex-alcoholic	1000	40
	54	Male	Floor of the mouth	T4aN2bM0	Ex-smoker	20	39	NA	NA	NA
	58	Male	Tongue	T4aN2bM0	Ex-smoker	40	46	Ex-alcoholic	1000	42
	76	Male	Tongue	T3N1M0	Ex-smoker	10	60	NA	NA	NA
	58	Male	Floor of the mouth	T4aN2cM0	NA	NA	NA	NA	NA	NA
	51	Female	Retromolar area	T3N3M0	Ex-smoker	10	36	Ex-alcoholic	2000	25
	72	Male	Floor of the mouth	T3N2cM0*	NA	NA	NA	NA	NA	NA
	59	Female	Retromolar area	T2N1M0	NA	NA	NA	NA	NA	NA
	59	Male	Tongue	T3N2bM0	NA	NA	NA	NA	NA	NA
	64	Male	Palate	T4aN3bM0	Ex-smoker	10	20	Never drank	-	-
	62	Female	Tongue	T3N2bM0	Never smoked	-	-	NA	NA	NA
	56	Male	Tongue	T4aN2bM0	Ex-smoker	20	45	Ex-alcoholic	500	27
	49	Male	Floor of the mouth	T3N3bM0	NA	NA	NA	NA	NA	NA
	57	Male	Oral cavity	T3N1M0*	NA	NA	NA	NA	NA	NA
N+	49	Female	Tongue	T3N1M0*	NA	NA	NA	NA	NA	NA
	61	Male	Floor of the mouth	T1N2M0	Ex-smoker	20	45	Ex-alcoholic	3000	44
	55	Male	Floor of the mouth	T1N3bN0	NA	NA	NA	NA	NA	NA
	61	Male	Tongue	T4aN2cM0*	NA	NA	NA	NA	NA	NA
	66	Female	Tongue	T4N3M0*	NA	NA	NA	NA	NA	NA
	66	Male	Tongue	T2N1Mx*	Ex-smoker	20	12	Ex-alcoholic	3000	33
	20	Male	Tongue	T4aN2bM0	Never smoked	-	-	Never drank	-	-
	70	Male	Alveolar ridge	T3N2bM0	NA	NA	NA	NA	NA	NA
	66	Male	Tongue	T4N1M0*	NA	NA	NA	NA	NA	NA
	61	Male	Tongue	T4N1Mx*	NA	NA	NA	NA	NA	NA
	54	Male	Floor of the mouth	T2N1M0	NA	NA	NA	NA	NA	NA
	69	Male	Floor of the mouth	T4aN1M0	Ex-smoker	30	52	Ex-alcoholic	4000	49
	77	Male	Tongue	T3N2M0*	Ex-smoker	30	22	Ex-alcoholic	1000	52
	68	Male	Tongue	T4aN3bM0	NA	NA	NA	NA	NA	NA
	61	Male	Floor of the mouth	T4aN2aM0	Current smoker	80	NA	NA	NA	NA

* clinical TNM
NA: no answer

Table S3. Quantitative performance metrics for the individual effect of LTA4H.

Models	auc_roc	balanced_acc	f1_ma_avg	f1_weighted	hamming	jaccard	loss_01	precision_ma_avg	recall_ma_avg	mean_gap
Stochastic Gradient Descent_opt	1.0	0.9	0.9	0.9	0.1	0.82	0.1	0.92	0.9	0.207
Quadratic Discriminant	1.0	0.9	0.9	0.9	0.1	0.82	0.1	0.92	0.9	0.223
CatBoost_opt	0.97	0.9	0.9	0.9	0.1	0.82	0.1	0.92	0.9	0.063
CatBoost	0.9	0.9	0.9	0.9	0.1	0.82	0.1	0.92	0.9	0.043
Gradient Boosting_opt	0.9	0.9	0.9	0.9	0.1	0.82	0.1	0.92	0.9	0.057
XGBClassifier_opt	0.9	0.9	0.9	0.9	0.1	0.82	0.1	0.92	0.9	0.08
LightGBM	0.9	0.9	0.9	0.9	0.1	0.82	0.1	0.92	0.9	0.097
LightGBM_opt	0.9	0.9	0.9	0.9	0.1	0.82	0.1	0.92	0.9	0.1
k-nearest neighbors_opt	0.9	0.9	0.9	0.9	0.1	0.82	0.1	0.92	0.9	0.107
Stochastic Gradient Descent	0.9	0.9	0.9	0.9	0.1	0.82	0.1	0.92	0.9	0.193
Random Forest_opt	0.88	0.9	0.9	0.9	0.1	0.82	0.1	0.92	0.9	0.067
AdaBoost_opt	0.88	0.9	0.9	0.9	0.1	0.82	0.1	0.92	0.9	0.067
k-nearest neighbors	0.88	0.9	0.9	0.9	0.1	0.82	0.1	0.92	0.9	0.103
Decision Tree_opt	0.87	0.9	0.9	0.9	0.1	0.82	0.1	0.92	0.9	0.057
Multi-layer Perceptron2_opt	0.8	0.9	0.9	0.9	0.1	0.82	0.1	0.92	0.9	0.063
Multi-layer Perceptron_opt	0.8	0.9	0.9	0.9	0.1	0.82	0.1	0.92	0.9	0.08
Logistic Regression	0.8	0.9	0.9	0.9	0.1	0.82	0.1	0.92	0.9	0.143
Multi-layer Perceptron2	0.8	0.9	0.9	0.9	0.1	0.82	0.1	0.92	0.9	0.143
Logistic Regression_opt	0.8	0.9	0.9	0.9	0.1	0.82	0.1	0.92	0.9	0.143
Support Vector_opt	0.8	0.9	0.9	0.9	0.1	0.82	0.1	0.92	0.9	0.15
Gaussian Process	0.8	0.9	0.9	0.9	0.1	0.82	0.1	0.92	0.9	0.153
Gaussian Process_opt	0.8	0.9	0.9	0.9	0.1	0.82	0.1	0.92	0.9	0.153
Support Vector	0.8	0.9	0.9	0.9	0.1	0.82	0.1	0.92	0.9	0.157
Multi-layer Perceptron	0.8	0.9	0.9	0.9	0.1	0.82	0.1	0.92	0.9	0.167
Quadratic Discriminant_opt	0.8	0.9	0.9	0.9	0.1	0.82	0.1	0.92	0.9	0.177
Gaussian Naive Bayes	0.8	0.9	0.9	0.9	0.1	0.82	0.1	0.92	0.9	0.183
Gaussian Naive Bayes_opt	0.8	0.9	0.9	0.9	0.1	0.82	0.1	0.92	0.9	0.183
Random Forest	0.82	0.72	0.71	0.71	0.28	0.55	0.28	0.73	0.72	0.25
XGBClassifier	0.8	0.62	0.6	0.6	0.38	0.43	0.38	0.64	0.62	0.28
AdaBoost	0.8	0.62	0.6	0.6	0.38	0.43	0.38	0.64	0.62	0.327
Gradient Boosting	0.8	0.62	0.6	0.6	0.38	0.43	0.38	0.64	0.62	0.327
Decision Tree	0.62	0.62	0.6	0.6	0.38	0.43	0.38	0.64	0.62	0.387
Multinomial Naive Bayes_opt	0.4	0.6	0.52	0.52	0.4	0.38	0.4	0.78	0.6	0.073
Multinomial Naive Bayes	0.4	0.5	0.33	0.33	0.5	0.25	0.5	0.25	0.5	0.023

Table S4. Quantitative performance metrics for the individual effect of COL6A1.

Models	auc_roc	balanced_acc	f1_ma_avg	f1_weighted	hamming	jaccard	loss_01	precision_ma_avg	recall_ma_avg	mean_gap
Multinomial Naive Bayes_opt	0.67	0.75	0.74	0.75	0.24	0.6	0.24	0.84	0.75	0.157
Quadratic Discriminant	0.79	0.71	0.71	0.71	0.29	0.55	0.29	0.71	0.71	0.113
Gradient Boosting_opt	0.77	0.71	0.71	0.71	0.29	0.55	0.29	0.71	0.71	0.14
CatBoost	0.75	0.71	0.71	0.71	0.29	0.55	0.29	0.71	0.71	0.217
Random Forest_opt	0.73	0.71	0.71	0.71	0.29	0.55	0.29	0.71	0.71	0.127
XGBClassifier_opt	0.73	0.71	0.71	0.71	0.29	0.55	0.29	0.71	0.71	0.147
AdaBoost	0.73	0.71	0.71	0.71	0.29	0.55	0.29	0.71	0.71	0.283
Stochastic Gradient Descent_opt	0.71	0.71	0.71	0.71	0.29	0.55	0.29	0.71	0.71	0.02
Stochastic Gradient Descent	0.71	0.71	0.71	0.71	0.29	0.55	0.29	0.71	0.71	0.07
LightGBM	0.71	0.71	0.71	0.71	0.29	0.55	0.29	0.71	0.71	0.093
AdaBoost_opt	0.71	0.71	0.71	0.71	0.29	0.55	0.29	0.71	0.71	0.143
Decision Tree	0.71	0.71	0.71	0.71	0.29	0.55	0.29	0.71	0.71	0.29
Decision Tree_opt	0.69	0.71	0.71	0.71	0.29	0.55	0.29	0.71	0.71	0.16
LightGBM_opt	0.69	0.71	0.71	0.71	0.29	0.55	0.29	0.71	0.71	0.16
Random Forest	0.69	0.71	0.71	0.71	0.29	0.55	0.29	0.71	0.71	0.297
Multi-layer Perceptron_opt	0.67	0.71	0.71	0.71	0.29	0.55	0.29	0.71	0.71	0.137
CatBoost_opt	0.67	0.71	0.71	0.71	0.29	0.55	0.29	0.71	0.71	0.18
Gaussian Process	0.67	0.71	0.71	0.71	0.29	0.55	0.29	0.71	0.71	0.183
Gaussian Process_opt	0.67	0.71	0.71	0.71	0.29	0.55	0.29	0.71	0.71	0.183
Multi-layer Perceptron2_opt	0.67	0.71	0.71	0.71	0.29	0.55	0.29	0.71	0.71	0.193
k-nearest neighbors	0.65	0.71	0.71	0.71	0.29	0.55	0.29	0.71	0.71	0.167
k-nearest neighbors_opt	0.65	0.71	0.71	0.71	0.29	0.55	0.29	0.71	0.71	0.173
XGBClassifier	0.65	0.71	0.71	0.71	0.29	0.55	0.29	0.71	0.71	0.267
Gradient Boosting	0.65	0.71	0.71	0.71	0.29	0.55	0.29	0.71	0.71	0.31
Logistic Regression	0.62	0.71	0.71	0.71	0.29	0.55	0.29	0.71	0.71	0.153
Multi-layer Perceptron	0.58	0.71	0.71	0.71	0.29	0.55	0.29	0.71	0.71	0.173
Support Vector_opt	0.54	0.71	0.71	0.71	0.29	0.55	0.29	0.71	0.71	0.19
Gaussian Naive Bayes	0.67	0.67	0.66	0.67	0.32	0.5	0.32	0.69	0.67	0.08
Gaussian Naive Bayes_opt	0.67	0.67	0.66	0.67	0.32	0.5	0.32	0.69	0.67	0.08
Multinomial Naive Bayes	0.67	0.62	0.57	0.58	0.36	0.43	0.36	0.8	0.62	0.057
Multi-layer Perceptron2	0.67	0.58	0.58	0.58	0.41	0.41	0.41	0.59	0.58	0.137
Support Vector	0.67	0.58	0.58	0.58	0.41	0.41	0.41	0.59	0.58	0.173
Logistic Regression_opt	0.67	0.58	0.58	0.58	0.41	0.41	0.41	0.59	0.58	0.183
Quadratic Discriminant_opt	0.67	0.58	0.58	0.58	0.41	0.41	0.41	0.59	0.58	0.203

Table S5. Quantitative performance metrics for the individual effect of CSTB.

Models	auc_roc	balanced_acc	f1_ma_avg	f1_weighted	hamming	jaccard	loss_01	precision_ma_avg	recall_ma_avg	mean_gap
CatBoost	0.67	0.75	0.73	0.73	0.26	0.58	0.26	0.83	0.75	0.193
Stochastic Gradient Descent	0.65	0.7	0.67	0.68	0.29	0.52	0.29	0.82	0.7	0.12
Random Forest	0.82	0.65	0.64	0.64	0.35	0.47	0.35	0.66	0.65	0.297
AdaBoost	0.8	0.65	0.64	0.64	0.35	0.47	0.35	0.66	0.65	0.303
Gradient Boosting	0.73	0.65	0.64	0.64	0.35	0.47	0.35	0.66	0.65	0.327
Decision Tree	0.65	0.65	0.64	0.64	0.35	0.47	0.35	0.66	0.65	0.353
Gaussian Naive Bayes	0.5	0.62	0.6	0.6	0.38	0.44	0.38	0.64	0.62	0.147
Gaussian Naive Bayes_opt	0.5	0.62	0.6	0.6	0.38	0.44	0.38	0.64	0.62	0.147
XGBClassifier	0.57	0.55	0.55	0.55	0.45	0.38	0.45	0.55	0.55	0.403
Quadratic Discriminant_opt	0.57	0.53	0.53	0.53	0.46	0.36	0.46	0.54	0.53	0.143
Quadratic Discriminant	0.47	0.52	0.47	0.47	0.48	0.32	0.48	0.53	0.52	0.117
Multinomial Naive Bayes_opt	0.77	0.5	0.34	0.35	0.49	0.26	0.49	0.26	0.5	0.073
Multinomial Naive Bayes	0.63	0.5	0.34	0.35	0.49	0.26	0.49	0.26	0.5	0.09
AdaBoost_opt	0.6	0.47	0.46	0.45	0.54	0.3	0.54	0.46	0.47	0.317
Multi-layer Perceptron2	0.57	0.47	0.46	0.45	0.54	0.3	0.54	0.46	0.47	0.193
Logistic Regression	0.57	0.47	0.46	0.45	0.54	0.3	0.54	0.46	0.47	0.213
Multi-layer Perceptron	0.57	0.47	0.46	0.45	0.54	0.3	0.54	0.46	0.47	0.283
Logistic Regression_opt	0.57	0.47	0.46	0.45	0.54	0.3	0.54	0.46	0.47	0.283
Multi-layer Perceptron_opt	0.57	0.47	0.46	0.45	0.54	0.3	0.54	0.46	0.47	0.283
XGBClassifier_opt	0.57	0.47	0.46	0.45	0.54	0.3	0.54	0.46	0.47	0.283
CatBoost_opt	0.53	0.47	0.46	0.45	0.54	0.3	0.54	0.46	0.47	0.35
k-nearest neighbors_opt	0.52	0.47	0.46	0.45	0.54	0.3	0.54	0.46	0.47	0.3
Support Vector	0.5	0.47	0.46	0.45	0.54	0.3	0.54	0.46	0.47	0.217
Random Forest_opt	0.5	0.47	0.46	0.45	0.54	0.3	0.54	0.46	0.47	0.32
Multi-layer Perceptron2_opt	0.5	0.47	0.46	0.45	0.54	0.3	0.54	0.46	0.47	0.333
Gradient Boosting_opt	0.48	0.47	0.46	0.45	0.54	0.3	0.54	0.46	0.47	0.357
Stochastic Gradient Descent_opt	0.47	0.47	0.46	0.45	0.54	0.3	0.54	0.46	0.47	0.263
LightGBM	0.47	0.47	0.46	0.45	0.54	0.3	0.54	0.46	0.47	0.28
LightGBM_opt	0.47	0.47	0.46	0.45	0.54	0.3	0.54	0.46	0.47	0.303
Decision Tree_opt	0.47	0.47	0.46	0.45	0.54	0.3	0.54	0.46	0.47	0.343
Gaussian Process	0.27	0.47	0.46	0.45	0.54	0.3	0.54	0.46	0.47	0.38
Gaussian Process_opt	0.27	0.47	0.46	0.45	0.54	0.3	0.54	0.46	0.47	0.38
k-nearest neighbors	0.58	0.45	0.45	0.45	0.55	0.29	0.55	0.45	0.45	0.303
Support Vector_opt	0.4	0.38	0.35	0.35	0.62	0.22	0.62	0.36	0.38	0.39

Table S6. Quantitative performance metrics for the combined effect of LTA4H, COL6A1, and CSTB.

Models	auc_roc	balanced_acc	f1_ma_avg	f1_weighted	hamming	jaccard	loss_01	precision_ma_avg	recall_ma_avg	mean_gap
AdaBoost_opt	0.76	0.78	0.78	0.78	0.22	0.63	0.22	0.79	0.78	0.07
Stochastic Gradient Descent_opt	0.79	0.77	0.77	0.78	0.22	0.63	0.22	0.78	0.77	0.027
Multi-layer Perceptron2_opt	0.77	0.74	0.74	0.74	0.26	0.59	0.26	0.74	0.74	0.017
Random Forest_opt	0.76	0.74	0.74	0.74	0.26	0.59	0.26	0.75	0.74	0.08
Gradient Boosting_opt	0.77	0.71	0.71	0.71	0.29	0.55	0.29	0.71	0.71	0.093
CatBoost_opt	0.77	0.71	0.71	0.71	0.29	0.55	0.29	0.71	0.71	0.097
CatBoost	0.76	0.71	0.71	0.71	0.29	0.55	0.29	0.71	0.71	0.193
Decision Tree_opt	0.75	0.71	0.71	0.71	0.29	0.55	0.29	0.72	0.71	0.103
LightGBM	0.73	0.71	0.71	0.71	0.29	0.55	0.29	0.71	0.71	0.157
LightGBM_opt	0.74	0.68	0.68	0.68	0.32	0.51	0.32	0.68	0.68	0.127
Logistic Regression_opt	0.77	0.67	0.67	0.67	0.33	0.5	0.33	0.69	0.67	0.013
Multi-layer Perceptron	0.77	0.67	0.67	0.67	0.33	0.5	0.33	0.69	0.67	0.037
Random Forest	0.72	0.65	0.65	0.65	0.35	0.48	0.35	0.65	0.65	0.327
Gradient Boosting	0.67	0.65	0.65	0.65	0.35	0.48	0.35	0.65	0.65	0.343
Decision Tree	0.65	0.65	0.65	0.65	0.35	0.48	0.35	0.65	0.65	0.35
XGBClassifier	0.64	0.65	0.65	0.65	0.35	0.48	0.35	0.65	0.65	0.353
Gaussian Process	0.8	0.64	0.63	0.63	0.36	0.46	0.36	0.66	0.64	0.097
Gaussian Process_opt	0.8	0.64	0.63	0.63	0.36	0.46	0.36	0.66	0.64	0.097
k-nearest neighbors	0.78	0.64	0.64	0.64	0.36	0.47	0.36	0.65	0.64	0.097
Multi-layer Perceptron_opt	0.77	0.64	0.64	0.64	0.36	0.47	0.36	0.65	0.64	0.09
Logistic Regression	0.76	0.64	0.63	0.63	0.36	0.46	0.36	0.66	0.64	0.023
Quadratic Discriminant_opt	0.76	0.64	0.61	0.61	0.36	0.45	0.36	0.68	0.64	0.043
Multi-layer Perceptron2	0.75	0.64	0.63	0.63	0.36	0.46	0.36	0.66	0.64	0.02
k-nearest neighbors_opt	0.74	0.64	0.64	0.64	0.36	0.47	0.36	0.65	0.64	0.097
XGBClassifier_opt	0.74	0.64	0.64	0.64	0.36	0.47	0.36	0.64	0.64	0.17
Support Vector_opt	0.75	0.61	0.59	0.59	0.4	0.42	0.4	0.63	0.61	0.037
AdaBoost	0.7	0.61	0.61	0.61	0.39	0.44	0.39	0.61	0.61	0.3
Stochastic Gradient Descent	0.6	0.58	0.58	0.58	0.42	0.41	0.42	0.58	0.58	0.017
Support Vector	0.77	0.57	0.55	0.54	0.43	0.38	0.43	0.59	0.57	0.063
Multinomial Naive Bayes	0.71	0.57	0.55	0.54	0.43	0.38	0.43	0.59	0.57	0.047
Multinomial Naive Bayes_opt	0.7	0.57	0.55	0.54	0.43	0.38	0.43	0.59	0.57	0.04
Gaussian Naive Bayes_opt	0.59	0.54	0.52	0.52	0.46	0.36	0.46	0.55	0.54	0.123
Gaussian Naive Bayes	0.59	0.54	0.52	0.52	0.46	0.36	0.46	0.55	0.54	0.127
Quadratic Discriminant	0.44	0.5	0.33	0.33	0.5	0.25	0.5	0.25	0.5	0.08

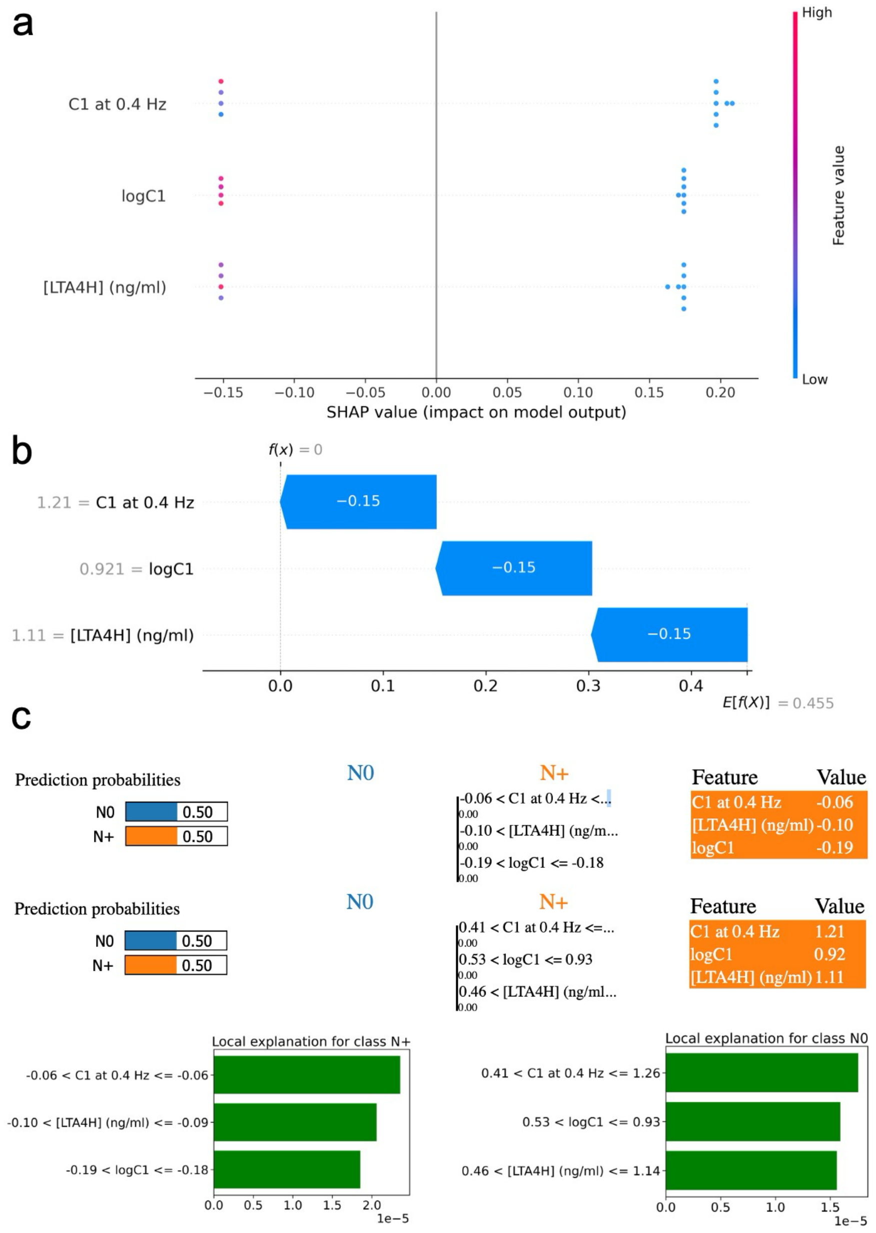

Figure S40. Interpretability of ML models considering the individual effect of LTA4H. a, SHAP (Shapley Additive Explanations) values show the impact of each feature on model output. **b,** Waterfall plot illustrating how SHAP values for each feature shift the model output from the baseline prediction (based on the background data distribution) to the final prediction, considering all feature contributions. In this case, all features showed a negative contribution. **c,** LIME (Local Interpretable Model-Agnostic Explanations) highlights the features that led to the correct prediction of an N+ and N0 sample.

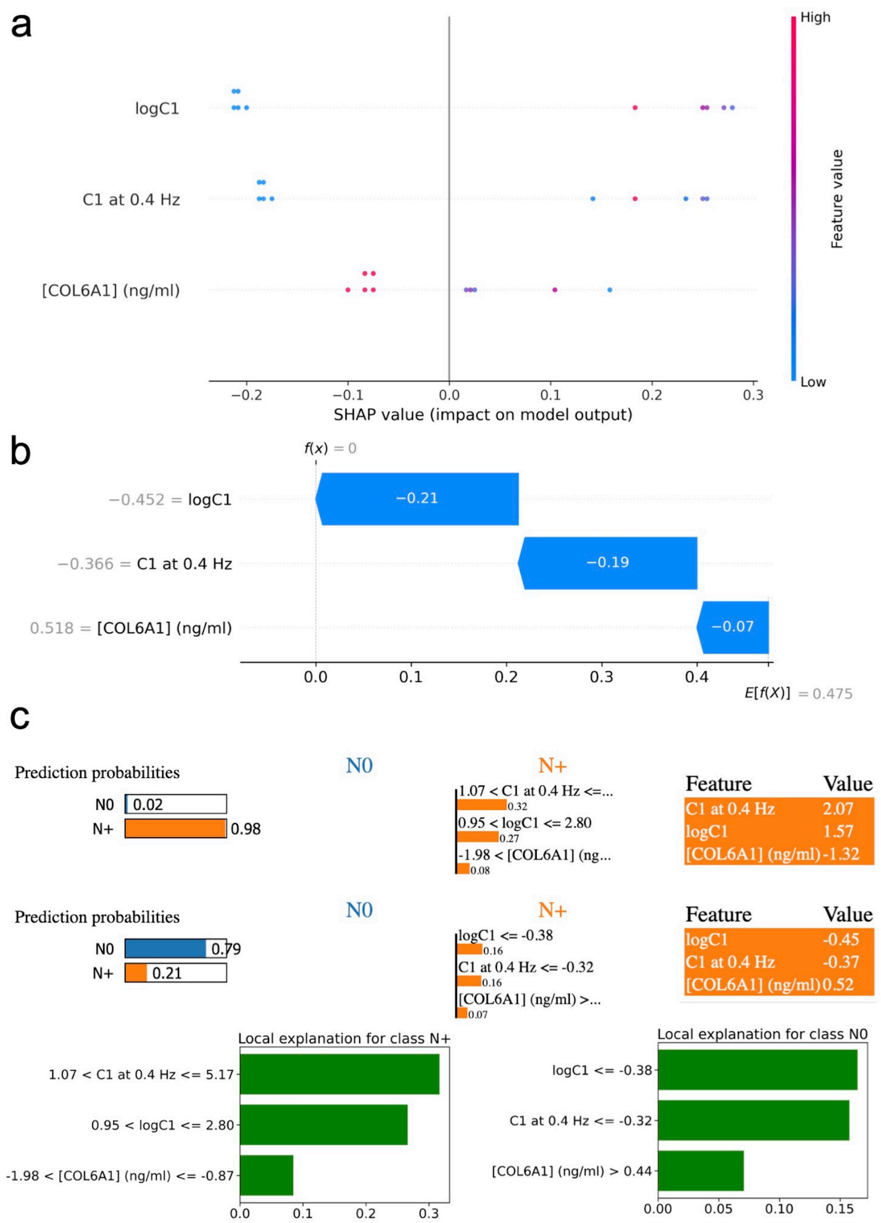

Figure S41. Interpretability of ML models considering the individual effect of COL6A1. a, SHAP values show the impact of each feature on model output. **b,** Waterfall plot illustrating how SHAP values for each feature shift the model output from the baseline prediction (based on the background data distribution) to the final prediction, considering all feature contributions. In this case, all features showed a negative contribution. **c,** LIME highlights the features that led to the correct prediction of an N+ and N0 sample.

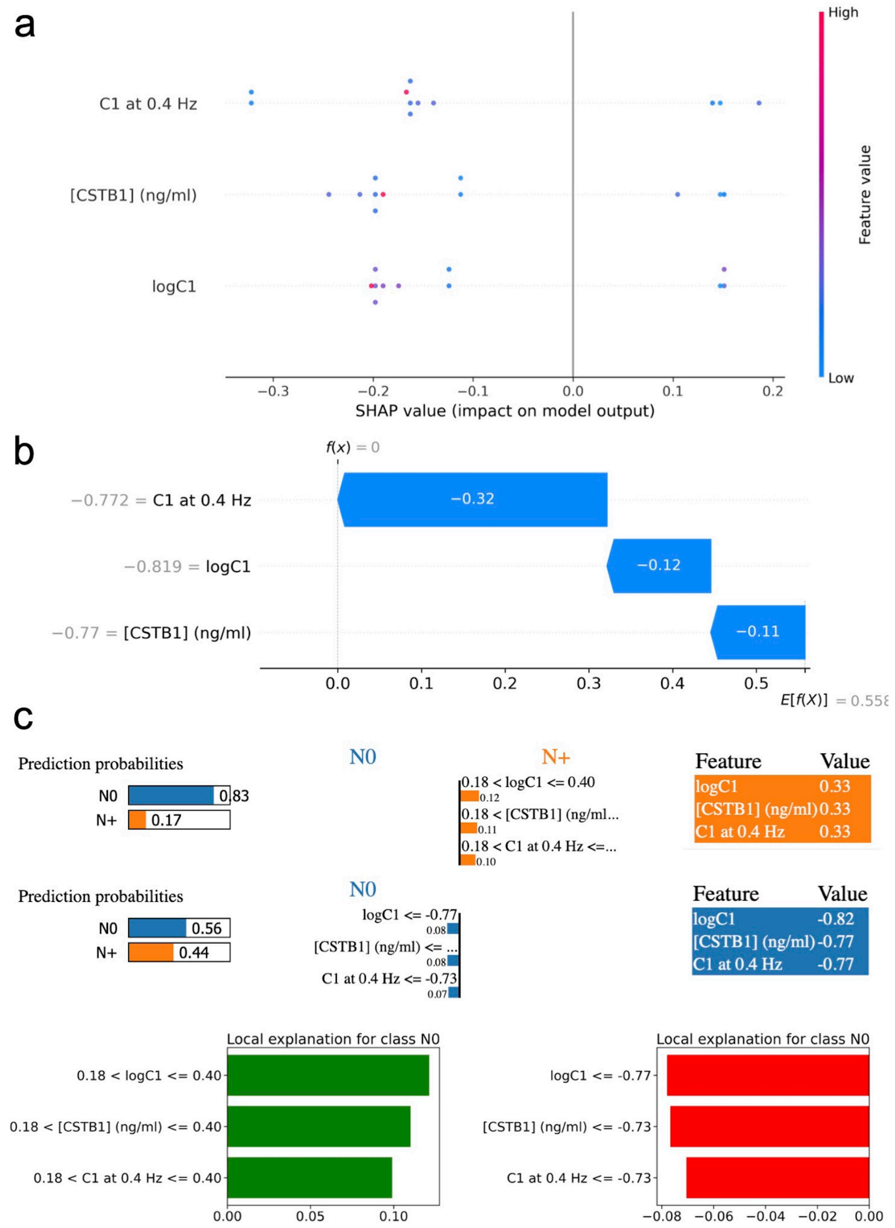

Figure S42. Interpretability of ML models considering the individual effect of CSTB. a, SHAP values show the impact of each feature on model output. **b,** Waterfall plot illustrating how SHAP values for each feature shift the model output from the baseline prediction (based on the background data distribution) to the final prediction, considering all feature contributions. In this case, all features showed a negative contribution. **c,** LIME highlights the features that led to the correct prediction of an N+ and N0 sample.

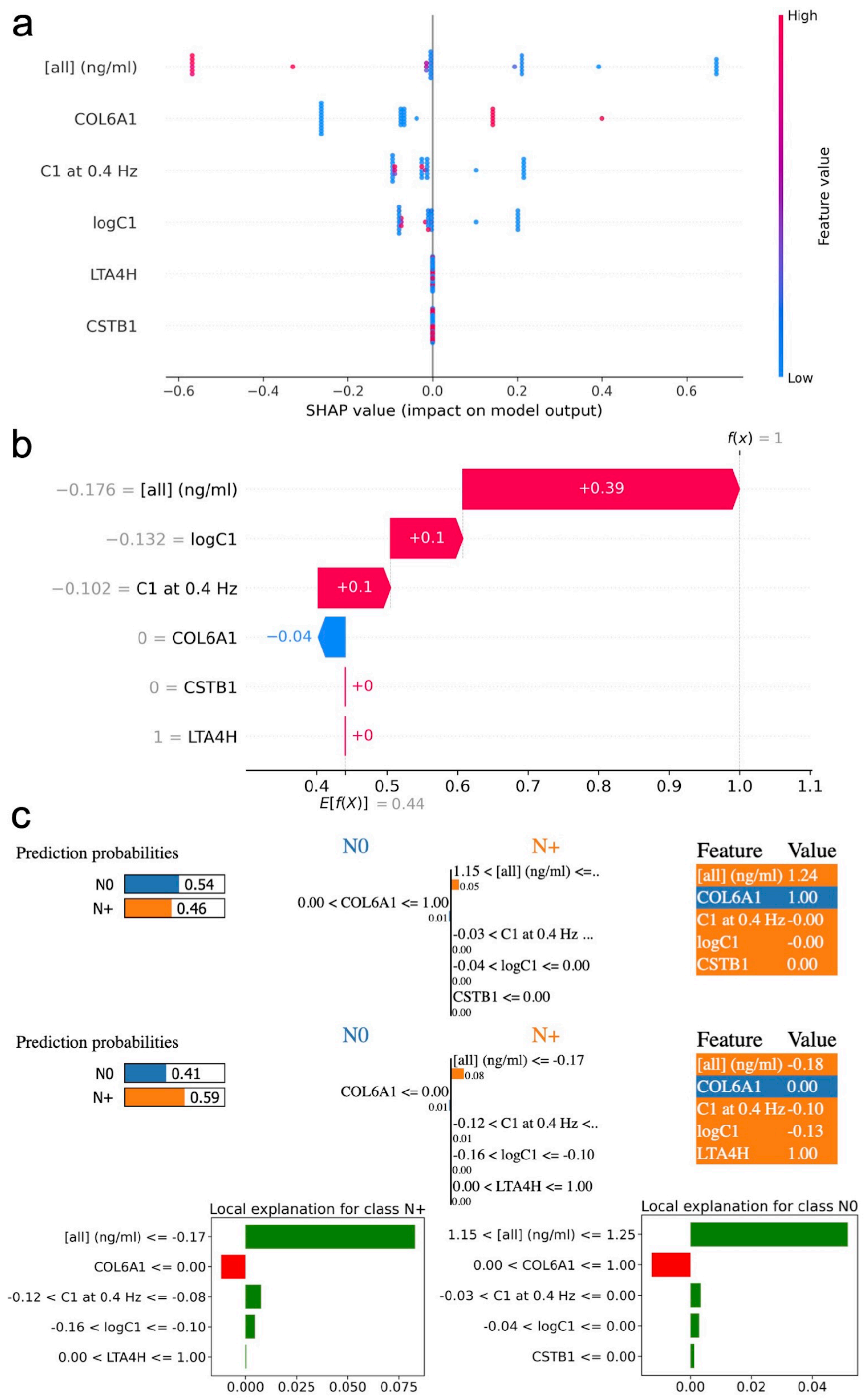

Figure S43. Interpretability of ML models considering the combined effect of LTA4H, COL6A1, and CSTB biomarkers. a, SHAP values show the impact of each feature on model output. **b,** Waterfall plot illustrating how SHAP values for each feature shift the model output from the baseline prediction (based on the background data distribution) to the final prediction, considering all feature contributions. In this case, most features showed a positive contribution. **c,** LIME highlights the features that led to the correct prediction of an N+ and N0 sample.
